# Synthesis of unsymmetrically substituted biaryls via sequential lithiation of dibromobiaryls using integrated microflow systems

**DOI:** 10.3762/bjoc.5.16

**Published:** 2009-04-29

**Authors:** Aiichiro Nagaki, Naofumi Takabayashi, Yutaka Tomida, Jun-ichi Yoshida

**Affiliations:** 1 Department of Synthetic Chemistry and Biological Chemistry, Graduate School of Engineering, Kyoto University, Kyotodaigakukatsura, Nishikyo-ku, Kyoto, 615-8510, Japan

**Keywords:** dibromobiaryls, fast mixing, integrated microflow system, selective lithiation, unsymmetrically substituted biaryls

## Abstract

A microflow system consisting of micromixers and microtube reactors provides an effective method for the introduction of two electrophiles onto dibromobiaryls. Selective monolithiation of dibromobiaryls, such as 2,2′-dibromobiphenyl, 4,4′-dibromobiphenyl, 2,7-dibromo-9,9-dioctylfluorene, 2,2′-dibromo-1,1′-binaphthyl, and 2,2′-dibromobibenzyl with 1 equiv of *n*-butyllithium followed by the reaction with electrophiles was achieved using a microflow system by virtue of fast micromixing and precise temperature control. Sequential introduction of two different electrophiles was achieved using an integrated microflow system composed of four micromixers and four microtube reactors to obtain unsymmetrically substituted biaryl compounds.

## Introduction

Unsymmetrical biaryls have received significant research interest because of the frequent occurrence of such structures in natural products, pharmaceuticals, agrochemicals, and functional organic materials [1]. Transition metal-catalyzed cross-coupling of arylmetal compounds with aryl halides or triflates serves as a useful method for preparation of such unsymmetrical biaryls [2–9]. Selective monolithiation of dihalobiaryls also seems to be useful for synthesis of unsymmetrically substituted biaryls because the remaining halogen atom can be utilized for further transformations [10–12]. However, halogen-lithium exchange reactions of dihalobiaryls usually give a mixture of mono- and dilithiated compounds in a conventional macrobatch reactor, even when one equivalent of butyllithium is used.

Recent investigations revealed that microflow systems [13–26] serve as useful method for improving product selectivity in fast competitive consecutive reactions. If a reaction is faster than mixing, the reaction takes place before the homogeneity of the solution is achieved. This often happens in macrobatch reactors such as flasks. In such cases, arguments based on kinetics do not work, and product selectivity is determined by the manner of mixing (disguised chemical selectivity) [27–28]. To obtain a predictable selectivity close to kinetically based one, extremely fast mixing is necessary. Micromixing based on short diffusion paths proved to be quite effective for this purpose [29–33]. Fast heat transfer by virtue of high surface-to-volume ratios in microspaces is also important for conducting fast reactions, because fast reactions are often highly exothermic. Fast reactions often involve unstable short-lived intermediates. In such cases residence time control in microflow system is effective for conducting reactions without decomposing such intermediates [34–40]. Moreover, microflow systems serve as effective ways of integrating chemical reactions, in which an initial product is used for a subsequent transformation [41–51]. For example, sequential introduction of two electrophiles to *p*-, *m*-, and *o*-dibromobenzenes based on Br-Li exchange reactions has been accomplished using an integrated microflow system at much higher temperatures than those for conventional macrobatch systems by virtue of residence time control and temperature control [52–53].

**Scheme 1:**
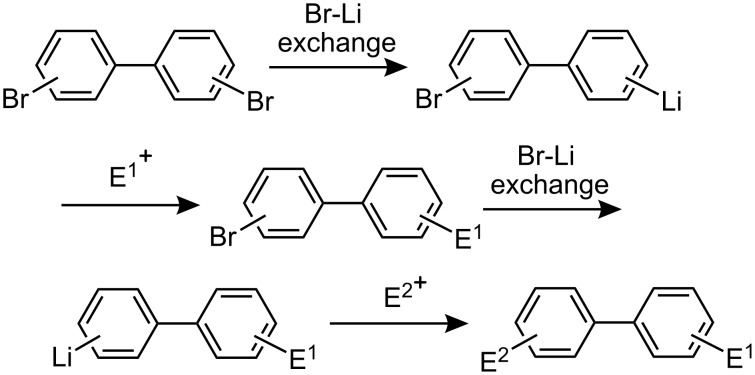
Sequential introduction of two electrophiles onto dibromobiaryls using Br-Li exchange reactions.

Recently, we reported that selective monolithiation can be achieved by extremely fast 1:1 micromixing of dibromobiaryls and *n*-butyllithium using microflow systems [54]. The successful results prompted us to perform a study on the synthesis of unsymmetrically substituted biaryls via sequential lithiation of dibromobiaryls using an integrated microflow system. These observations may open a new aspect of the synthesis of unsymmetrically substituted biaryls (Scheme 1), and herein we report full details of this study.

## Results and Discussion

### Br-Li Exchange Reaction of 2,2′-Dibromobiphenyl

**Scheme 2:**
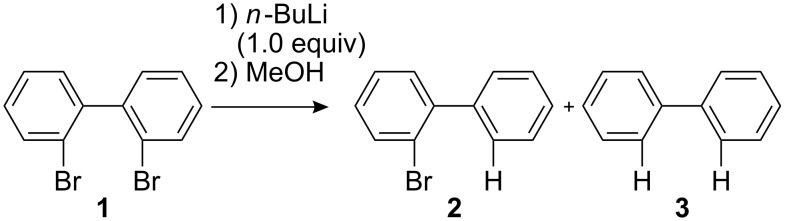
Br-Li exchange reaction of 2,2′-dibromobiphenyl (**1**) with *n*-BuLi using a conventional macrobatch reactor.

First, we focused on Br-Li exchange reaction of 2,2′-dibromobiphenyl (**1**) to generate (2-bromobiphenyl-2′-yl)lithium. It is known that Br-Li exchange reaction of 2,2′-dibromobiphenyl (**1**) gives a significant amount of dilithiated product in a conventional macrobatch system [55–56]. In order to confirm this, we reexamined the Br-Li exchange reaction of **1** with 1 equiv of *n*-BuLi in a conventional macrobatch reactor (Scheme 2). A hexane solution of *n*-BuLi was added dropwise (1 min) to a THF solution of **1** in a 20 mL round-bottomed flask at *T* °C (*T* = −78, −48, −27, 0, and 24) to generate (2-bromobiphenyl-2′-yl)lithium. After stirring methanol was added, and the mixture was stirred for 10 min. Then, the solution was analyzed by gas chromatography (GC) to determine the yields of 2-bromobiphenyl (**2**, product derived from monolithiation) and biphenyl (**3**, product derived from dilithiation).

As shown in Table 1, **2** was obtained with high selectivity at −78 °C, but the yield was not very high. Moreover, the requirement of such low temperatures like −78 °C causes severe limitations in the industrial application. The selectivity decreased with an increase in the temperature, and a significant amount of **3** was produced at higher temperatures. The major side product was 2-bromo-2′-butylbiphenyl (**4**), which seemed to be produced by the reaction of (2-bromobiphenyl-2′-yl)lithium with 1-bromobutane that was produced by Br-Li exchange reaction.

**Table 1:** Br-Li exchange reaction of 2,2′-dibromobiphenyl (**1**) using a conventional macrobatch system.*^a^*

temperature (°C)	reaction time (min)	**1** conversion (%)*^b^*	**2** yield (%)*^b^*	**3** yield (%)*^b^*	**4** yield (%)*^b^*

−78	60	94	76	4	0
−48	10	86	69	4	0
−27	10	81	48	18	0
0	10	75	36	25	2
24	10	66	14	34	3

*^a^*A solution of **1** (0.10 M, 6.0 mL) in THF was stirred in a flask (20 mL). A solution of *n*-BuLi (0.50 M, 1.2 mL) in hexane was added dropwise for 1.0 min. After stirring, methanol (neat, 3.0 mL) was added dropwise for 1.0 min. After stirring for 10 min, the mixture was analyzed. *^b^*Determined by GC.

**Figure 1:**
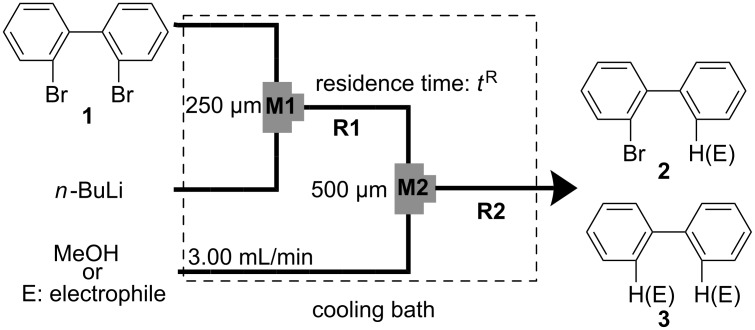
Microflow system for Br-Li exchange reaction of 2,2′-dibromobiphenyl (**1**). T-shaped micromixer: **M1** (ø = 250 μm) and **M2** (ø = 500 μm), microtube reactor: **R1** and **R2** (ø = 1000 μm, *l* = 50 cm), a solution of 2,2′-dibromobiphenyl (**1**): 0.10 M in THF, a solution of *n*-BuLi: 0.50 M in hexane, a solution of electrophile: 0.30 M in THF.

In the next step, the same reaction was examined using a microflow system composed of two T-shaped micromixers (**M1** and **M2**) and two microtube reactors (**R1** and **R2**) shown in Figure 1. A solution of 2,2′-dibromobiphenyl (**1**) (0.10 M) in THF (flow rate: 6.00 ml·min^−1^, 0.60 mmol min^−1^) and a solution of *n*-BuLi (0.50 M) in hexane (flow rate: 1.20 ml·min^−1^, 0.60 mmol min^−1^) were introduced to **M1** (ø = 250 μm) by syringe pumping. The mixture was passed through **R1** (residence time = *t*^R^ s) and was introduced to **M2** (ø = 500 μm), where methanol (neat, flow rate: 3.00 ml·min^−1^) was introduced. The resulting mixture was passed through **R2** (ø = 1000 μm, *l* = 50 cm, *t*^R^ = 2.3 s). The temperature for the Br-Li exchange reaction and that for the quenching with methanol were controlled by adjusting the temperature of a cooling bath. The residence time (*t*^R^) was adjusted by changing the length of the microtube reactor **R1** with a fixed flow rate. After a steady state was reached, an aliquot of the product solution was taken for 60 s. The amount of 2-bromobiphenyl (**2**) and that of biphenyl (**3**) were determined by GC.

**Figure 2:**
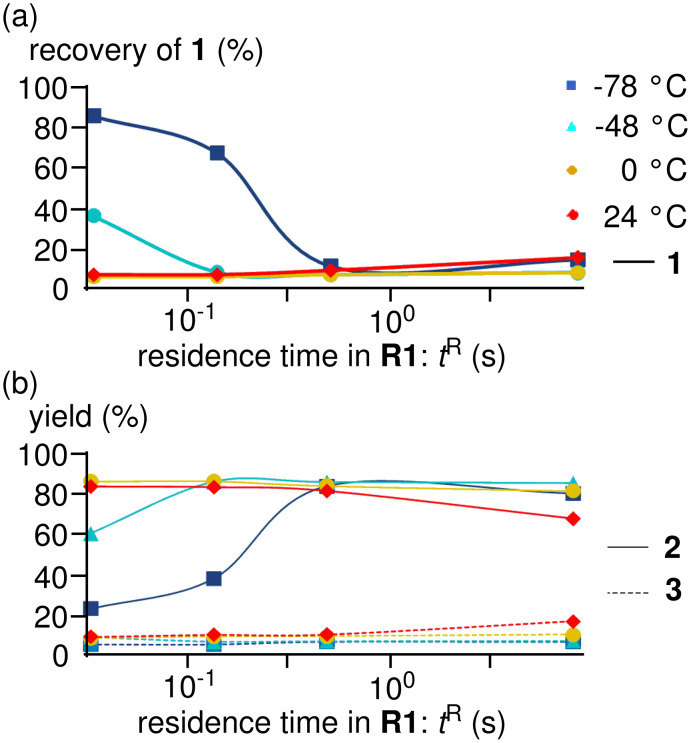
Effect of temperature and residence time in Br-Li exchange reaction of 2,2′-dibromobiphenyl (**1**) using the microflow system; (a) plots of recovery of **1** against the residence time, (b) plots of yields of **2** and **3** against the residence time. Flow rate of a solution of 2,2′-dibromobiphenyl (**1**): 6.00 ml·min^−1^, flow rate of *n*-BuLi: 1.20 ml·min^−1^, flow rate of methanol: 3.00 ml·min^−1^. Yields of **1**, **2** and **3** were determined by GC.

The results obtained with varying temperature (−78 to 24 °C) and residence time (*t*^R^) in **R1** (0.057–13 s) are shown in Figure 2 (see the Supporting Information for details). High yields and high selectivities were obtained even at 0 °C (*t*^R^ = 0.057 s: **2** (88%) and **3** (3%)), and 24 °C (*t*^R^ = 0.057 s: **2** (85%) and **3** (4%)), demonstrating a significant advantage of the microflow system. Extremely fast heat transfer of the microflow system seems to be responsible for these results. In other words, Br-Li exchange reaction can be inherently conducted at 0 °C and 24 °C, while the reaction in macrobatch reactors suffers from insufficient heat removal, and therefore, over-cooling is necessary. At −48 °C and −78 °C, the Br-Li exchange reaction was not complete within 0.1 s. At higher temperatures, however, the Br-Li exchange reaction was complete within 0.1 s.

As shown in Table 2, the selectivity increased with a decrease in the diameter of **M1**, presumably because faster mixing can be achieved with a mixer of a smaller diameter. The selectivity also increased with an increase in the flow rate. It is known that the mixing rate increases with an increase in the flow rate for other types of micromixers [57]. These observations indicate that extremely fast 1:1 mixing in the microflow system is responsible for selective monolithiation at much higher temperatures such as 0 °C and 24 °C than those required for a conventional macrobatch processes.

**Table 2:** Effect of flow rate and inner diameter of **M1** at 0 °C.*^a^*

flow rate of **1**/THF (mL/min)	flow rate of *n*-BuLi/hexane (mL/min)	inner diameter of **M1** (μm)	**1** conversion (%)*^b^*	**2** yield (%)*^b^*	**3** yield (%)*^b^*

6.00	1.20	250	97	88	3
3.00	0.600	250	90	80	7
1.50	0.300	250	76	57	15
0.600	0.120	250	69	41	19
6.00	1.20	500	93	77	7
6.00	1.20	800	79	62	9

*^a^***R1**: ø = 500 μm, *l* = 3.5 cm, flow rate of methanol: 3.00 ml·min^−1^. *^b^* Determined by GC.

Under the optimized reaction conditions (reaction temperature: 0 °C, *t*^R^: 0.057 s), reactions using other electrophiles (0.30 M in THF) were examined. In all cases, the conversion of **1** was higher than 95%. As summarized in Table 3, the reaction with iodomethane gave 2-bromo-2′-methylbiphenyl (**5**) in 89% yield with high selectivity. Chlorotrimethylsilane, benzaldehyde, and benzophenone were also effective as electrophiles, and the corresponding products derived from the monolithiated species were obtained selectively in high yields. These results show that the microflow system serves as an useful method for selective monolithiation of 2,2′-dibromobiphenyl (**1**) followed by the reaction with electrophiles.

**Table 3:** Br-Li exchange reaction of 2,2′-dibromobiphenyl (**1**) followed by reaction with an electrophile using the microflow system.*^a^*

electrophile	yield (%)*^c^*	
	monosubstituted product	major byproduct

MeI*^b^*	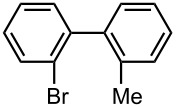 **5**	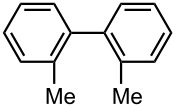 **6**
	89	Trace
Me_3_SiCl*^b^*	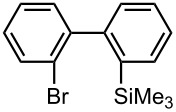 **7**	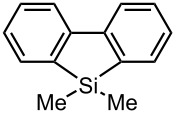 **8**
	80	3
PhCHO	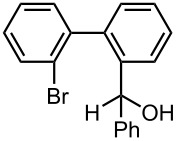 **9**	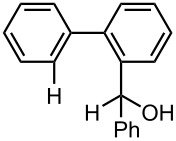 **10**
	90	Trace
PhCOPh	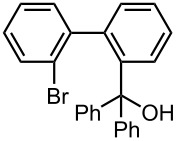 **11**	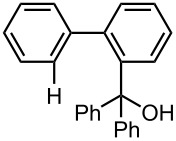 **12**
	93	2

*^a^*Flow rate of a solution of **1**: 6.00 ml·min^−1^, flow rate of *n*-BuLi/hexane: 1.20 ml·min^−1^, **R1**: ø = 500 μm, *l* = 3.5 cm (*t*^R^ = 0.057 s). The conversion was higher than 95% in all cases. *^b^* **R2**: ø = 1000 μm, *l* = 200 cm, flow rate of a solution of an electrophile: 4.00 ml·min^−1^. *^c^*Determined by GC.

### Synthesis of Unsymmetrically Substituted Biaryls from 2,2′-Dibromobiphenyl (**1**) by Sequential Introduction of Two Electrophiles

**Figure 3:**
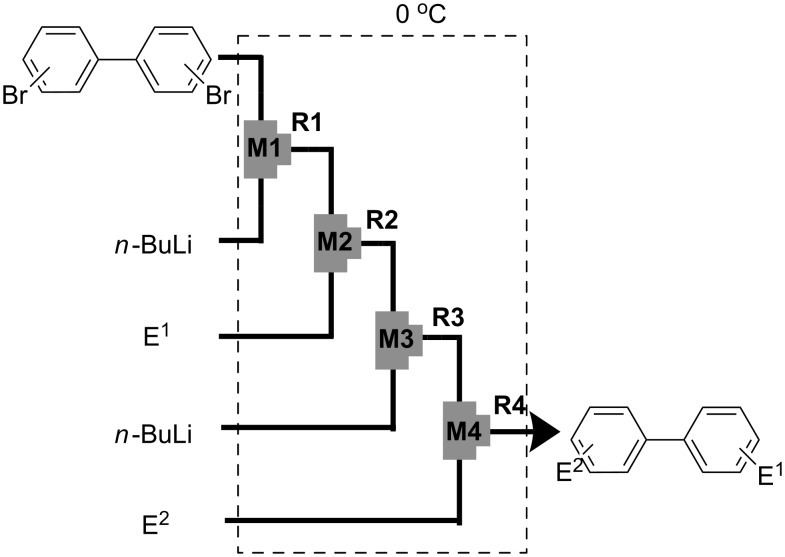
A microflow system for sequential introduction of two electrophiles. T-shaped micromixer: **M1** (ø = 250 μm), **M2** (ø = 500 μm), **M3** (ø = 500 μm), and **M4** (ø = 500 μm), microtube reactor: **R1** (ø = 500 μm, *l* = 3.5 cm (*t*^R^: 0.057 s)), **R2** (ø = 1000 μm, *l* = 200 cm (*t*^R^: 9.8 s)), **R3** (ø = 1000 μm, *l* = 200 cm (*t*^R^: 8.5 s)), and **R4** (ø = 1000 μm, *l* = 50 cm (*t*^R^: 1.8 s)). Flow rate of a solution of a dibromobiaryl (0.10 M in THF): 6.00 ml·min^−1^, flow rate of *n*-BuLi (0.50 M in hexane): 1.20 ml·min^−1^, flow rate of a solution of a first electrophile (E^1^) (0.50 M in THF): 2.40 ml·min^−1^. flow rate of *n*-BuLi (1.00 M in hexane): 1.44 ml·min^−1^, flow rate of a solution of a second electrophile (E^2^) (0.90 M in THF): 2.00 ml·min^−1^.

With successful monolithiation of 2,2′-dibromobiphenyl (**1**) followed by the reaction with an electrophile using the microflow system in hand, sequential introduction of two electrophiles (E^1^ and E^2^) onto 2,2′-dibromobiphenyl (**1**) was examined using an integrated microflow system composed of four T-shaped micromixers (**M1**, **M2**, **M3** and **M4**) and four microtube reactors (**R1**, **R2**, **R3** and **R4**) shown in Figure 3. In this case, T-shaped micromixers **M3** and **M4** having the inside diameter of 500 μm were used to suppress the pressure increase because an increase in the numbers of micromixers and microtube reactors in the system causes a signficant pressure increase.

A solution of 2,2′-dibromobiphenyl (**1**) (0.10 M) in THF (flow rate: 6.00 ml·min^−1^, 0.60 mmol min^−1^) and a solution of *n*-BuLi (0.50 M) in hexane (flow rate: 1.20 ml·min^−1^, 0.60 mmol·min^−1^) were introduced to **M1** (ø = 250 μm) by syringe pumping. The mixture was passed through **R1** (ø = 500 μm, *l* = 3.5 cm, *t*^R^ = 0.057 s) and the resulting solution was introduced to **M2** (ø = 500 μm), where a solution of a first electrophile (E^1^) (0.50 M) in THF (flow rate: 2.40 ml·min^−1^, 1.20 mmol·min^−1^) was introduced. The mixture was passed through **R2** (ø = 1000 μm, *l* = 200 cm, *t*^R^ = 9.8 s) and the resulting solution containing a monobromo compound was introduced to **M3** (ø = 500 μm), where a solution of *n*-BuLi (1.0 M) in hexane (flow rate: 1.44 ml·min^−1^, 1.44 mmol·min^−1^) was introduced. The mixture was passed through **R3** (ø = 1000 μm, *l* = 200 cm, *t*^R^ = 8.5 s) and the resulting solution containing a second aryllithium intermediate was introduced to **M4** (ø = 500 μm), where a solution of a second electrophile (E^2^) (0.90 M) in THF (flow rate: 2.00 mL·min^−1^, 1.80 mmol·min^−1^) was introduced. Themixture was passed through **R4** (ø = 1000 μm, *l* = 50 cm, *t*^R^ = 1.8 s). The all processes were conducted at 0 °C.

As summarized in Table 4, the sequential introductions of two electrophiles were achieved successfully with various combinations of electrophiles without isolation of monobromo biaryl compound. It is interesting to note that the use of an aldehyde (E^1^) and methyl chlorocarbonate (E^2^) as electrophiles led to effective formation of a seven-membered ring lactone. This integrated microflow synthesis serves as a straightforward and powerful method for synthesizing unsymmetrically substituted biaryls from 2,2′-dibromobiphenyl (**1**) and two electrophiles.

**Table 4:** Synthesis of unsymmetrically substituted biaryls from 2,2′-dibromobiphenyl (**1**) by sequential introduction of two electrophiles.*^a^*

dibromobiaryl	electrophile	yield (%)

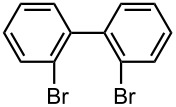 **1**	E^1^: MeI E^2^: PhCHO	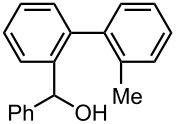 **13**
		70 *^b,e^*
	E^1^: MeI E^2^: Me_3_SiCl	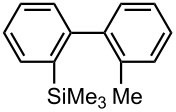 **14**
		82 *^c,f^*
	E^1^: MeI E^2^: MeOCOCl	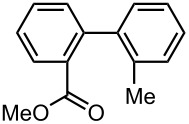 **15**
		76 *^f^*
	E^1^: PhCHO E^2^: MeOCOCl	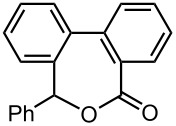 **16**
		75 *^d,f^*

*^a^*Reactions were conducted in the microflow system shown in Figure 3 unless otherwise stated. *^b^*2-Methylbiphenyl was also produced as a byproduct. *^c^***R4**: ø = 1000 μm, *l* = 200 cm *^ d^***R2**: ø = 1000 μm, *l* = 50 cm, benzaldehyde (0.30 M), methyl chlorocarbonate (0.30 M) *^e^*Isolated yield. *^f^*Determined by GC.

### Br-Li Exchange Reaction of 4,4′-Dibromobiphenyl

**Scheme 3:**
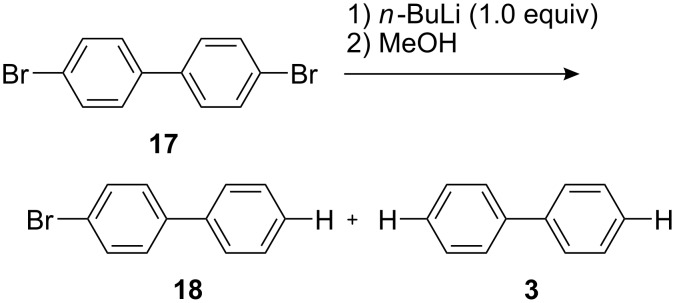
Br-Li exchange reaction of 4,4′-dibromobiphenyl (**17**) with *n*-BuLi using a conventional macrobatch reactor.

Next, we examined Br-Li exchange reaction of 4,4′-dibromobiphenyl (**17**) with *n*-BuLi. The temperature effect for a macrobatch reaction was studied (Scheme 3) and the results are summarized in Table 5.

4-Bromobiphenyl (**18**) was obtained with high yield and selectivity at −78 °C. However, the yield and selectivity decreased with an increase in the temperature. At higher temperatures 4-bromo-4′-butylbiphenyl (**19**) was produced presumably by the reaction of (4-bromobiphenyl-4′-yl)lithium with 1-bromobutane that was produced by Br-Li exchange reaction.

**Table 5:** Br-Li exchange reaction of 4,4′-dibromobiphenyl (**17**) using a conventional macrobatch system.*^a^*

temperature (°C)	reaction time (min)	**17** conversion (%)*^b^*	**18** yield (%)*^b^*	**3** yield (%)*^b^*	**19** yield (%)*^b^*

−78	60	95	87	5	0
−48	10	90	49	5	0
−27	10	81	56	5	2
0	10	86	47	6	13
24	10	87	25	2	26

*^a^*A solution of **17** (0.10 M, 6.0 mL) in THF was stirred in a flask (20 mL). A solution of *n*-BuLi (0.50 M, 1.2 mL) in hexane was added dropwise for 1.0 min. After stirring, methanol (neat, 3.0 mL) was added dropwise for 1.0 min. After stirring for 10 min, the mixture was analyzed. *^b^*Determined by GC.

**Figure 4:**
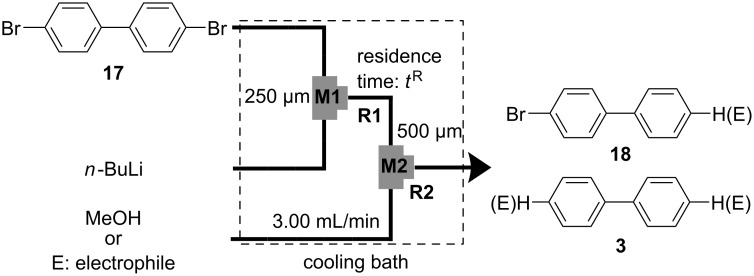
Microflow system for Br-Li exchange reaction of 4,4′-dibromobiphenyl (**17**). T-shaped micromixer: **M1** (ø = 250 μm) and **M2** (ø = 500 μm), microtube reactor: **R1** and **R2** (ø = 1000 μm, *l* = 50 cm), a solution of 4,4′-dibromobiphenyl (**17**): 0.10 M in THF, a solution of *n*-BuLi: 0.50 M in hexane, a solution of an electrophile: 0.30 M in THF.

In the next step, the reaction was carried out using a microflow system composed of two T-shaped micromixers (**M1** and **M2**) and two microtube reactors (**R1** and **R2**) shown in Figure 4. A solution of 4,4′-dibromobiphenyl (**17**) (0.10 M) in THF (flow rate: 6.00 mL·min^−1^, 0.60 mmol·min^−1^) and a solution of *n*-BuLi (0.50 M) in hexane (flow rate: 1.20 mL·min^−1^, 0.60 mmol·min^−1^) were introduced to **M1** (ø = 250 μm) by syringe pumping. The mixture was passed through **R1** (residence time = *t*^R^ sec) and was introduced to **M2** (ø = 500 μm), where methanol (neat, flow rate: 3.00 ml·min^-1^) was introduced. The resulting mixture was passed through **R2** (ø = 1000 μm, *l* = 50 cm, *t*^R^ = 2.3 s). The temperature for the Br-Li exchange reaction and the quenching with methanol was controlled by adjusting the temperature of a cooling bath. The residence time was adjusted by changing the length of the microtube reactor **R1** with a fixed flow rate. After a steady state was reached, an aliquot of the product solution was taken for 60 s. The amount of 4-bromobiphenyl (**18**, product derived from monolithiation) and biphenyl (**3**, product derived from dilithiation) was determined by GC.

**Figure 5:**
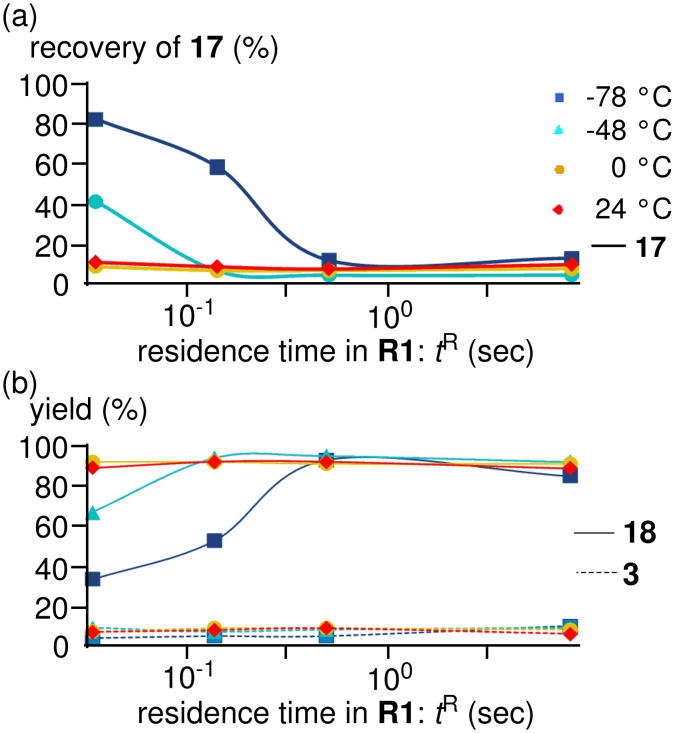
Effect of temperature and residence time in Br-Li exchange reaction of 4,4′-dibromobiphenyl (**17**) using the microflow system; (a) Plots of recovery of **17** against residence time, (b) Plots of yields of **18** and **3** against residence time. Flow rate of a solution of 4,4′-dibromobiphenyl (**17**): 6.00 mL·min^−1^, flow rate of *n*-BuLi/hexane: 1.20 mL·min^−1^, flow rate of methanol: 3.00 mL·min^−1^. Yields of **3**, **17** and **18** were determined by GC.

The results obtained with varying temperature (−78 to 24 °C) and residence time in **R1** (0.057–13 s) are shown in Figure 5 (see the Supporting Information for details). High yields and high selectivities were obtained even at 0 °C (residence time of **R1** = 0.057 s: **18** (88%) and **3** (4%)) and 24 °C (residence time of **R1** = 0.057 s: **18** (85%) and **3** (4%)). At −48 °C and −78 °C, the Br-Li exchange reaction was not complete within 0.1 s. At higher temperatures, however, the Br-Li exchange reaction was complete within 0.1 s. These tendencies are quite similar to those for the 2,2′-dibromobiphenyl (**1**) case. Therefore, it is reasonable to consider that (2-bromobiphenyl-2′-yl)lithium and (4-bromobiphenyl-4′-yl)lithium have similar thermal stability.

Under the optimized reaction conditions (reaction temperature: 0 °C, *t*^R^: 0.057 s), reactions using other electrophiles (0.30 M in THF) such as iodomethane, benzaldehyde, and benzophenone were examined to obtain the corresponding products in good yields at 0 °C as shown in Table 6. These results show that microflow system serves as an useful method for selective monolithiation of 4,4′-dibromobiphenyl (**17**) followed by the reaction with electrophiles.

**Table 6:** Br-Li exchange reaction of 4,4′-dibromobiphenyl (**17**) followed by the reaction with an electrophile using the microflow system.*^a^*

electrophile	yield (%)	
	monosubstituted product	disubstituted product

MeI*^b^*	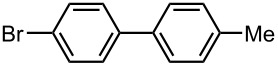 **20**	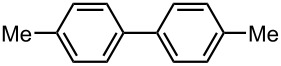 **21**
	85 *^c^*	4 *^c^*
PhCHO	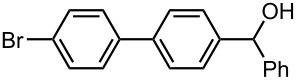 **22**	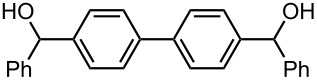 **23**
	83 *^d^*	6 *^d^*
PhCOPh	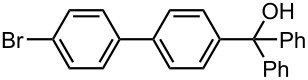 **24**	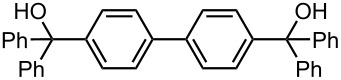 **25**
	84 *^d^*	5 *^d^*

*^a^* Flow rate of a solution of **17**: 6.00 mL·min^−1^, flow rate of *n*-BuLi/hexane: 1.20 mL·min, **R1**: ø = 500 μm, *l* = 3.5 cm (*t*^R^ = 0.057 s). *^b^* **R2**: ø = 1000 μm, *l* = 200 cm, flow rate of a solution of an electrophile: 4.00 mL·min^−1^. *^c^*Determined by GC. *^d^*Isolated yield.

### Synthesis of Unsymmetrically Substituted Biaryls from 4,4′-Dibromobiphenyl (**17**) by Sequential Introduction of Two Electrophiles

With successful monolithiation of 4,4′-dibromobiphenyl (**17**) followed by the reaction with an electrophile using the microflow system in hand, sequential introduction of two electrophiles (E^1^ and E^2^) onto 4,4′-dibromobiphenyl (**17**) was carried out using an integrated microflow system composed of four T-shaped micromixers and four microtube reactors shown in Figure 3. As summarized in Table 7, the sequential introductions of two electrophiles were achieved successfully without isolation of a monobromo biaryl compound.

**Table 7:** Synthesis of unsymmetrically substituted biaryls from 4,4′-diboromobiphenyl (**17**).*^a^*

dibromobiaryl	electrophile	yield (%)*^b^*

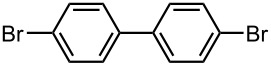 **17**	E^1^: MeI E^2^: Me_3_SiCl	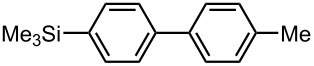 **26**
		71 *^c^*
	E^1^: Me_3_SiCl E^2^: MeI	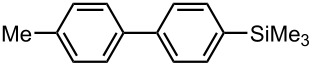 **26**
		75 *^d^*
	E^1^: MeI E^2^: MeOCOCl	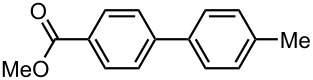 **27**
		56

*^a^*Reactions were conducted in the microflow system shown in Figure 3 unless otherwise stated. *^b^*Determined by GC. *^c^***R4**: ø = 1000 μm, *l* = 200 cm. *^d^***R4**: ø = 1000 μm, *l* = 200 cm, flow rate of a solution of chlorotrimethylsilane (0.30 M): 2.00 mL·min^−1^, flow rate of a solution of iodomethane (0.50 M): 2.40 mL·min^−1^.

### Br-Li Exchange Reaction of Other Dibromobiaryls and 2,2′-Dibromobibenzyl (**43**)

We next examined the reactions of other dibromobiaryls such as 2,7-dibromo-9,9-dioctylfluorene (**28**), and 2,2′-dibromo-1,1′-binaphthyl (**36**) [58] using the microflow system. As summarized in Table 8, monolithiation was achieved with high selectivity even at 0 °C and 24 °C. Such high selectivity is difficult to obtain with macrobatch reactors at similar temperatures such as 0 °C. It is also noteworthy that monolithiation of 2,2′-dibromobibenzyl (**43**) can be achieved with high selectivity even at 0 °C and 24 °C. The resulting organolithium intermediate reacted with various electrophiles to give the corresponding products in high yields with high selectivity.

**Table 8:** Br-Li exchange reaction of other dibromobiaryls (**28**, **36**) and 2,2′-dibromobibenzyl (**43**) followed by reactions with electrophiles.

dibromo compound	reaction system*^a^*	temp. (°C)	electrophile		yield (%)	
					monosubstituted product	disubstituted product

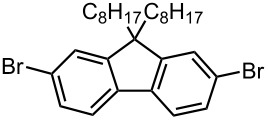 **28**					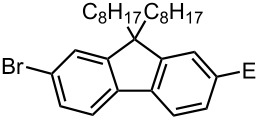	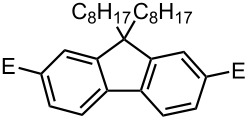
	macrobatch*^b,c^*	−78	MeOH	E: -H	89	6
					(**29**)	(**30**)
	macrobatch*^c,d^*	0	MeOH	E: -H	54*^f^*	5
	microflow	0	MeOH	E: -H	95	4
	microflow	24	MeOH	E: -H	92	5
	microflow *^e^*	0	MeI	E: -Me	93	3
					(**32**)	(**33**)
	microflow	0	PhCHO	E:	90*^g^*	2*^g^*
				-CH(OH)Ph	(**34**)	(**35**)
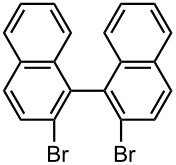 **36**					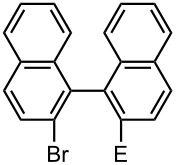	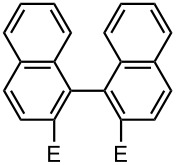
	macrobatch*^b,c^*	−78	MeOH	E: -H	90	10
					(**37**)	(**38**)
	macrobatch*^c,d^*	0	MeOH	E: -H	86	13
	microflow	0	MeOH	E: -H	93	1
	microflow	24	MeOH	E: -H	92	2
	microflow*^e^*	0	MeI	E: -Me	85	Trace
					(**39**)	(**40**)
	microflow	0	PhCHO	E:	82*^g^*	trace*^g^*
				-CH(OH)Ph	(**41**)	(**42**)
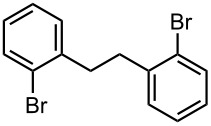 **43**					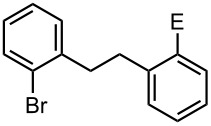	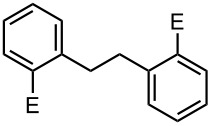
	macrobatch*^b,c^*	−78	MeOH	E: -H	85	10
					(**44**)	(**45**)
	macrobatch*^c,d^*	0	MeOH	E: -H	27	15
	microflow	0	MeOH	E: -H	80	10
	microflow	24	MeOH	E: -H	77	11
	microflow*^e^*	0	Mel	E: -Me	81	4
					(**46**)	(**47**)
	microflow	0	PhCHO	E:	66*^g^*	7*^g^*
				-CH(OH)Ph	(**48**)	(**49**)

*^a^*Microflow reactions were carried out under the following conditions unless otherwise stated. **R1**: ø = 500 μm, *l* = 3.5 cm, **R2**: ø = 1000 μm, *l* = 50 cm, flow rate of a solution of a dibromobiaryl compound: 6.00 mL·min^−1^, flow rate of *n*-BuLi/hexane: 1.20 mL·min^−1^, flow rate of a solution of an electrophile: 3.00 mL·min^−1^. Yields were determined by GC otherwise stated. *^b^*Reaction time: 60 min. *^c^*A solution of dibromo compound (0.10 M, 6.0 mL) in THF was stirred in a flask (20 mL). A solution of *n*-BuLi (0.50 M, 1.2 mL) in hexane was added dropwise for 1.0 min. After stirring, methanol (neat, 3.0 mL) was added dropwise for 1.0 min. After stirring for 10 min, the mixture was analyzed. *^d^*Reaction time: 10 min. *^e^***R2**: ø = 1000 μm, *l* = 200 cm, flow rate of a solution of an electrophile: 4.00 mL·min^−1^. *^f^* 2-Bromo-7-butyl-9,9-dioctylfluorene (**31**) was also produced as a byproduct. *^g^*Isolated yield.

### Synthesis of Unsymmetrically Substituted Biaryls by Sequential Introduction of Two Electrophiles

With successful monolithiation of dibromobiaryls such as 2,7-dibromo-9,9-dioctylfluorene (**28**) and 2,2′-dibromobinaphthyl (**36**) followed by the reaction with an electrophile using the microflow system in hand, sequential introduction of two electrophiles (E^1^ and E^2^) onto dibromobiaryls was carried out using an integrated microflow system composed of four T-shaped micromixers and four microtube reactors shown in Figure 3.

As summarized in Table 9, the sequential introductions of two electrophiles were achieved successfully with various combinations of electrophiles without isolation of monobromo biaryl compounds. This integrated microflow synthesis serves as a straightforward and powerful method for synthesizing unsymmetrically substituted biaryls from dibromobiaryls and two electrophiles.

**Table 9:** Synthesis of unsymmetrially substituted biaryls from dibromobiaryls by sequential introduction of two electrophiles.*^a^*

dibromobiaryl	electrophile	yield (%)

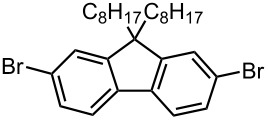 **28**	E^1^: MeI E^2^: MeOCOCl	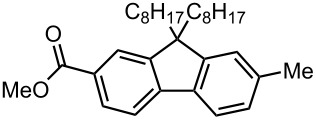 **50**
		51 *^b^*
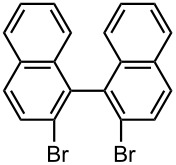 **36**	E^1^: MeI E^2^: PhCHO	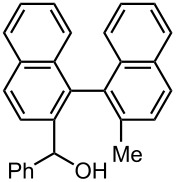 **51**
		71 *^c^*
	E^1^: MeI E^2^: PhCOPh	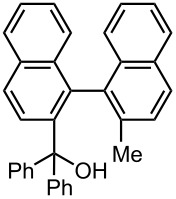 **52**
		78 *^c^*

*^a^*Reactions were conducted in the microflow system shown in Figure 3 unless otherwise stated. *^b^*Determined by GC. *^c^*Isolated yield.

In conclusion, we have developed an efficient method for selective monolithiation of dibromobiaryls at 0 °C by virtue of fast mixing in microflow systems. Electrophiles was introduced on one of the aromatic rings with high selectivity. Sequential introduction of two electrophiles by repeating the sequence have also been achieved using the integrated microflow systems. Unsymmetrically substituted biaryls were obtained in high selectivity. The results obtained in this study speak well for the potential of integrated microflow systems in multi-step synthesis in flash chemistry [59–61], and the method adds a new dimension to the synthesis of unsymmetrically substituted biaryls.

## Supporting Information

Experimental procedures and full spectroscopic data for all new compounds are provided as supporting information.

File 1 Experimental procedures for compounds **1**–**52**.

File 2 ^1^H NMR spectra and ^13^C NMR spectra of compounds **4**, **5**, **9**–**16**, **19**, **22**–**26**, **31**–**35**, **39**.

File 3 ^1^H NMR spectra and ^13^C NMR spectra of compounds **41**, **42**, **46**, **48**–**52**.
