# Supplementary material for: Synthesis of unsymmetrically substituted biaryls via sequential lithiation of dibromobiaryls using integrated microflow systems
Source: Beilstein J Org Chem. 2009 Apr 29;5:16. doi: 10.3762/bjoc.5.16 (PMC2686311; doi:10.3762/bjoc.5.16)
Supplement: File 1 — Experimental procedures for compounds 1–52. [file Beilstein_J_Org_Chem-05-16-s001.pdf]

Synthesis of unsymmetrically substituted biaryls via sequential lithiation of dibromobiaryls using integrated microflow systems

Aiichiro Nagaki, Naofumi Takabayashi, Yutaka Tomida, and Jun-ichi Yoshida*

Address: Department of Synthetic Chemistry and Biological Chemistry, Graduate School of Engineering, Kyoto University, Kyotodaigakukatsura, Nishikyo-ku, Kyoto, 615-8510, Japan

Email: Jun-ichi Yoshida - yoshida@sbchem.kyoto-u.ac.jp

* Corresponding author

Supporting Information

Experimental procedures for compounds 1–52

General

GC analysis was performed on a SHIMADZU GC-2014 gas chromatograph equipped with a flame ionization detector using a fused silica capillary column (column, CBP1; 0.25 mm x 25 m; initial oven temperature, 50 °C; rate of temperature increase, 10 °C /min). ^{1}H and ^{13}C NMR spectra were recorded on Varian MERCURYplus-400 (^{1}H 400 MHz, ^{13}C 100 MHz) spectrometer or JEOLECA-600 (^{13}C 150 MHz) spectrometer with Me_4Si or $CHCl_3$ as a standard in $CDCl_3$ unless otherwise noted. EI mass spectra were recorded on JMS-SX102A spectrometer. FAB mass spectra were recorded on JMX-HX110A spectrometer. Gel permeation chromatography was carried out with Japan Analytical Industry LC-9201. 2,2'-Dibromobiphenyl, 2-bromobiphenyl, biphenyl, 4,4'-dibromobiphenyl, 4-bromobiphenyl, 2,7-dibromo-9,9-dioctylfluorene, 2-bromo-9,9-dioctylfluorene, 9,9-dioctylfluorene, 2,2'-dibromo-1,1'-binaphthyl, 2-bromo-1,1'-binaphthyl, 1,1'-binaphthyl, bibenzyl, iodomethane, chlorotrimethylsilane, benzaldehyde, benzophenone, and methyl chlorocarbonate were commercially available. Hexane was purchased from Wako, distilled before use, and stored over molecular sieves (4 Å). THF was purchased from Kanto as a dry solvent. Stainless steel (SUS304) T-shaped micromixers having inner diameter of 250, 500 and 800 μm were manufactured by Sanko Seiki Co., Inc. Stainless steel (SUS316) microtube reactors having inner diameter of 500 and 1000 μm were purchased from GL Sciences. The micromixers and microtube reactors were connected with stainless steel fittings (GL Sciences, 1/16 OUW). The microflow system was dipped in a cooling bath to control the temperature. Harvard Model 11, equipped with gastight syringes purchased from SGE was used for introducing the solution to a microflow system.

Typical Procedure for the Br-Li Exchange Reaction of Dibromobiaryls Followed by the Reaction with Methanol in a Macrobatch System.

A solution of dibromobiaryls (0.10 M, 6.0 mL) in THF was stirred in a flask (20 mL round bottom glass flask with a magnetic stirrer). A solution of *n*-BuLi (0.50 M, 1.2 mL) in hexane was added dropwise for 1.0 min. After stirring, methanol (neat, 3.0 mL) was added dropwise for 1.0 min. After stirring for 10 min, a cooling bath was removed. The mixture was analyzed by GC.

Typical Procedure for the Br-Li Exchange Reaction of Dibromobiaryls Followed by the Reaction with Electrophiles in a Microflow System.

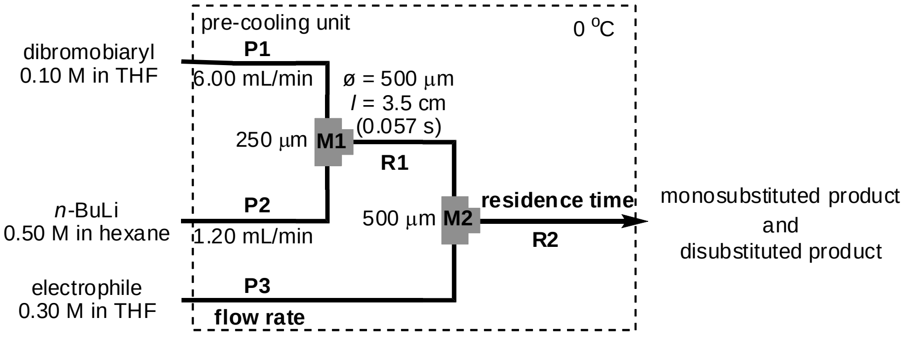

A microflow system consisting of two T-shaped micromixers (**M1** and **M2**), two microtube reactors (**R1** and **R2**) and three microtube pre-cooling units [**P1** (inner diameter ø = 1000 µm, length *l* = 100 cm), **P2** (ø = 1000 µm, *l* = 50 cm) and **P3** (ø = 1000 µm, *l* = 100 cm)] was used (0 °C). A solution of a dibromobiaryl (0.10 M) in THF (flow rate = 6.00 mL·min-1) and a solution of *n*-BuLi (0.50 M) in hexane (flow rate = 1.20 mL·min^{-1}) were introduced to **M1** (ø = 250 µm). The resulting solution was passed through **R1** (ø = 500 µm, *l* = 3.5 cm) and was mixed with a solution of an electrophile (0.30 M) in THF in **M2** (ø = 500 µm). The resulting solution was passed through **R2**. After a steady state was reached, the product solution was collected for 60 s and was treated with H_2O to quench the reaction. 2,2'-Dibromobiphenyl (**1**), 4,4'-dibromobiphenyl (**17**), 2,7-dibromo-9,9-dioctylfluorene (**28**),

2,2'-dibromo-1,1'-binaphthyl (**36**), and 2,2'-dibromobibenzyl (**43**) were used as dibromobiaryls. Methanol, iodomethane, chlorotrimethylsilane, benzaldehyde, and benzophenone were used as electrophiles.

The reactions of dibromobiaryls with iodomethane and chlorotrimethylsilane were carried out under the following conditions.

R2: ø = 1000 μm, *l* = 200 cm (8.4 s), flow rate of a solution of an electrophile: 4.00 mL·min-1

The reactions of dibromobiaryls with methanol, benzaldehyde, and benzophenone were carried out under the following conditions. When methanol was used as a electrohpile, reaction temperature of 24 °C was also examined.

R2: ø = 1000 μm, *l* = 50 cm (2.3 s), flow rate of a solution of an electrophile: 3.00 mL·min^{-1}

The Br-Li Exchange Reaction of 2,2'-Dibromobiphenyl (1) Followed by the Reaction with Methanol in a Macrobatch System.

The results are summarized in Table S-1.

Table S-1: The Br-Li exchange reaction of 2,2'-dibromobiphenyl (**1**) followed by the reaction with methanol in a macrobatch system.

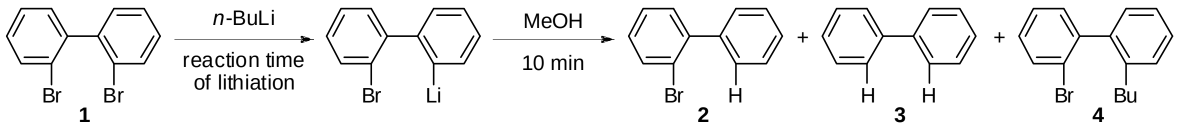

reaction temperature (°C)	reaction time of lithiation (min)	**1** conv. (%)	**2** yield (%)	**3** yield (%)	**4** yield (%)
−78	60	94	76	4	0
−48	10	86	69	4	0
−27	10	81	48	18	0
0	10	75	36	25	2
24	10	66	14	34	3

2-Bromo-2'-butylbiphenyl (4): ^{1}H NMR (400 MHz, $CDCl_3$) δ 0.76 (t, *J* = 7.2 Hz, 3H), 1.18 (sext., *J* = 7.4 Hz, 2H), 1.32–1.53 (m, 2H), 2.27–2.52 (m, 2H), 7.10 (dd, *J* = 7.6, 1.2 Hz, 1H), 7.16–7.36 (m, 6H), 7.62–7.68 ppm (m, 1H); ^{13}C NMR (100 MHz, $CDCl_3$, some signals overlapped) δ 13.8, 22.4, 32.8, 124.0, 125.3, 126.9, 127.9, 128.6, 128.9, 129.6, 131.2, 132.5, 140.6, 140.7, 142.5 ppm; HRMS (EI) *m/z* calcd for $C_{16}H_{17}Br$: 288.0514, found: 288.0513.

Typical Procedure for Effects of the Residence Time and Temperature in the Br-Li Exchange Reaction of Dibromobiaryls Followed by the Reaction with Methanol in the Microflow System.

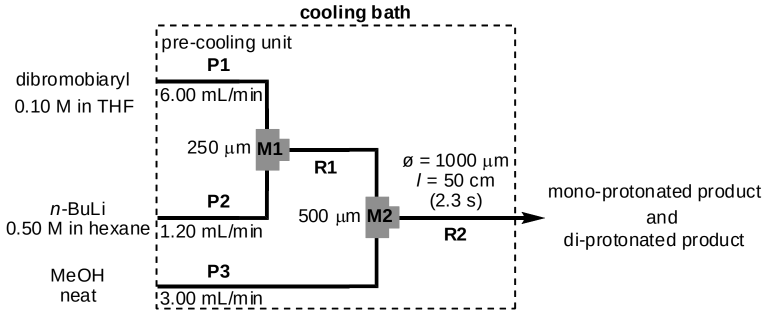

A microflow system consisting of two T-shaped micromixers (**M1** and **M2**), two microtube reactors (**R1** and **R2**) and three microtube pre-cooling units [**P1** (inner diameter ø = 1000 μm, length *l* = 100 cm), **P2** (ø = 1000 μm, *l* = 50 cm) and **P3** (ø = 1000 μm, *l* = 100 cm)] was used. A solution of dibromobiaryl (0.10 M) in THF (flow rate = 6.00 mL·min^{-1}) and a solution of *n*-BuLi (0.50 M) in hexane (flow rate = 1.20 mL·min^{-1}) were introduced to **M1** (ø = 250 μm). The resulting solution was passed through **R1** and was mixed with methanol (neat, flow rate = 3.00 mL·min^{-1}) in **M2** (ø = 500 μm). The resulting solution was passed through **R2** (ø = 1000 μm, *l* = 50 cm). After a steady state was reached, the product solution was collected for 60 s. The mixture was analyzed by GC. The results obtained with varying the residence time in **R1**, and bath temperature in the cooling bath is summarized in Table S-2. The residence time was controlled by changing the inner diameter (ø) and the length (*l*) of **R1** with the fixed flow rate. 2,2'-Dibromobiphenyl (**1**), and 4,4'-dibromobiphenyl (**17**) were used as dibromobiaryls.

Table S-2. Effects on the residence time and bath temperature in the Br-Li exchange reaction of 2,2'-dibromobiphenyl followed by the reaction with methanol in the microflow system.

residence time of **R1** (s)	inner diameter of **R1** (μm)	length of **R1** (cm)	bath temperature (°C)	**1** conv. (%)	**2** yield (%)	**3** yield (%)
0.057	500	3.5	−78	23	19	0
			−48	69	60	4
			0	97	88	3
			24	96	85	4
0.23	1000	3.5	−78	40	36	0
			−48	95	88	1
			0	97	88	4
			24	96	85	5
0.82		12.5	−78	92	86	1
			−48	96	88	2
			0	96	86	4
			24	94	83	5
13		200	−78	89	82	1
			−48	95	87	2
			0	95	83	5
			24	88	68	12

Effects of the Flow Rate and the Inner Diameter of M1 in the Br-Li Exchange Reaction of 2,2'-Dibromobiphenyl (1) Followed by the Reaction with Methanol in the Microflow System.

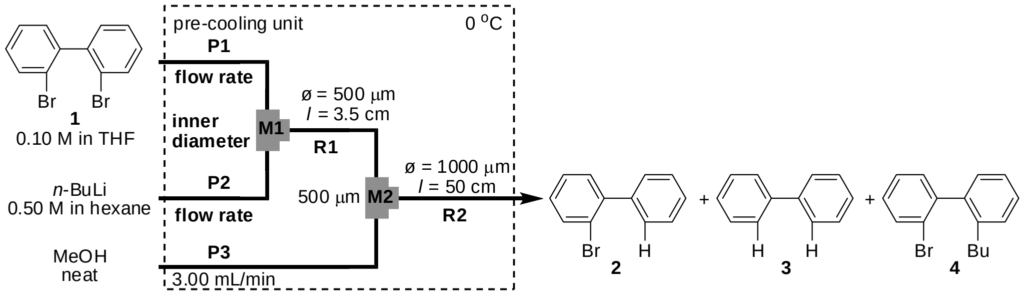

A microflow system consisting of two T-shaped micromixers (**M1** and **M2**), two microtube reactors (**R1** and **R2**) and three microtube pre-cooling units [**P1** (inner diameter ø = 1000 µm, length l = 100 cm), **P2** (ø = 1000 µm, l = 50 cm) and **P3** (ø = 1000 µm, l = 100 cm)] was used (0 °C). A solution of 2,2'-dibromobiphenyl (**1**) (0.10 M) in THF and a solution of *n*-BuLi (0.50 M) in hexane were introduced to **M1**. The resulting solution was passed through **R1** and was mixed with methanol (neat, flow rate = 3.00 mL·min^{-1}) in **M2** (ø = 500 µm). The resulting solution was passed through **R2** (ø = 1000 µm, l = 50 cm). After a steady state was reached, the product solution was collected for 60 s. The mixture was analyzed by GC. The results obtained with varying the inner diameter in **M1**, the flow rate of a solution of 2,2'-dibromobiphenyl (**1**), and the flow rate of *n*-BuLi in hexane are summarized in Table S-3.

Table S-3. Effects of the flow rate and the inner diameter of **M1** in the Br-Li exchange reaction of 2,2'-dibromobiphenyl (**1**) followed by the reaction with methanol at 0 °C in the microflow system.

flow rate of a solution of **1** (mL/min)	flow rate of *n*-BuLi/hexane (mL/min)	inner diameter of **M1** (μm)	**1** conv. (%)	**2** yield (%)	**3** yield (%)
6.00	1.20	250	97	88	3
3.00	0.600	250	90	80	7
1.50	0.300	250	76	57	15
0.600	0.120	250	69	41	19
6.00	1.20	500	93	77	7
6.00	1.20	800	79	62	9

The Br-Li Exchange Reaction of 2,2'-Dibromobiphenyl (1) Followed by the Reaction with Iodomethane in the Microflow System.

GC analysis of the reaction mixture indicated that 2-bromo-2'-methylbiphenyl (**5**) (GC t_R 19.9 min) was produced in 89% yield, and 2,2'-dimethylbiphenyl (**6**) (GC t_R 17.6 min) was produced in a trace amount (95% conv.). **2-Bromo-2'-methylbiphenyl (5)**: ^{1}H NMR (400 MHz, $CDCl_3$) δ 2.09 (s, 3H), 7.08 (d, J = 7.6 Hz, 1H), 7.12–7.32 (m, 6H), 7.59–7.64 ppm (m, 1H); ^{13}C NMR (100 MHz, $CDCl_3$) δ 20.0, 123.6, 125.3, 127.0, 127.7, 128.5, 129.0, 129.6, 130.7, 132.3, 135.7, 140.9, 142.4 ppm; HRMS (EI) *m/z* calcd for $C_{13}H_{11}Br$: 246.0044, found: 246.0040. The spectral data of 2,2'-dimethylbiphenyl (**6**) were identical to those reported in the literature.[1]

The Br-Li Exchange Reaction of 2,2'-Dibromobiphenyl (1) Followed by the Reaction with Chlorotrimethylsilane in the Microflow System.

GC analysis of the reaction mixture indicated that 2-bromo-2'-trimethylsilylbiphenyl (**7**) (GC t_R 22.3 min) was produced in 80% yield and 9,9-dimethyl-9-silafluorene (**8**) (GC t_R 20.4 min) was produced in 3% yield (97% conv.). The spectral data of 2-bromo-2'-trimethylsilylbiphenyl (**7**) and 9,9-dimethyl-9-silafluorene (**8**) were identical to those reported in the literature.[2]

The Br-Li Exchange Reaction of 2,2'-Dibromobiphenyl (1) Followed by the Reaction with Benzaldehyde in a Microflow System.

GC analysis of the reaction mixture indicated that 2-bromo-2'-[hydroxyl(phenyl)methyl]biphenyl (**9**) (GC t_R 29.0 min) was produced in 90% yield as a mixture of two diastereomers (diastereomer ratio = 52/48 determined by NMR), and 2-[hydroxyl(phenyl)methyl]biphenyl (**10**) (GC t_R 26.9 min) was produced in a trace amount (98% conv.). **2-Bromo-2'-[hydroxyl(phenyl)methyl]biphenyl (9)**: ^{1}H NMR (400 MHz, $CDCl_3$) δ 2.05–2.18 (m) and 2.30–2.42 (m) (total 1H, two diastereomers), 5.64 (s) and 5.67 (s) (total 1H, two diastereomers), 6.92–7.71 ppm (m, 13H); ^{13}C NMR (100 MHz, $CDCl_3$, a mixture of two diasteromers, some signals overlapped) δ 72.7, 72.8, 123.5, 124.2, 126.46, 126.54, 126.6, 126.99, 127.02, 127.1, 127.18, 127.21, 127.23, 127.4, 128.1, 128.4, 128.6, 129.1, 129.3, 130.1, 131.2, 131.7, 132.3, 132.7, 139.5, 140.3, 141.1, 141.2, 141.4, 141.8, 142.7, 143.2 ppm; HRMS (EI) *m/z* calcd for $C_{19}H_{15}BrO$: 338.0306, found: 338.0303. **2-[hydroxyl(phenyl)methyl]biphenyl (10)**: ^{1}H NMR (400 MHz, $CDCl_3$) δ 2.13-2.24 (m, 1H), 5.90 (s, 1H), 7.10–7.56 ppm (m, 14H); ^{13}C NMR (100 MHz, $CDCl_3$, some signals overlapped) δ 72.3, 126.6, 127.15, 127.16, 127.4, 127.8, 128.1, 128.2, 129.3, 130.0, 140.8, 141.0, 141.3, 143.8 ppm; HRMS (EI) *m/z* calcd for $C_{19}H_{16}O$: 260.1201, found: 260.1199.

The Br-Li Exchange Reaction of 2,2'-Dibromobiphenyl (1) Followed by the Reaction with Benzophenone in a Microflow System.

GC analysis of the reaction mixture indicated that 2-bromo-2'-[hydroxyl(diphenyl)methyl]biphenyl (**11**) (GC t_R 33.6 min) was produced in 93% yield and 2-[hydroxyl(diphenyl)methyl]biphenyl (**12**) (GC t_R 31.0 min) was produced in 2% yield (95% conv.). **2-Bromo-2'-[hydroxyl(diphenyl)methyl]biphenyl (11)**: ^{1}H NMR (400 MHz, $CDCl_3$) δ 2.69 (d, J = 4.0 Hz, 1H), 6.32–6.38 (m, 1H), 6.82–6.90 (m, 2H), 6.98–7.32 (m, 14H), 7.56 ppm (dd, J = 8.4, 1.6 Hz, 1H); ^{13}C NMR (100 MHz, $CDCl_3$) δ 83.1, 124.4, 126.0, 126.7, 126.8, 126.9, 127.0, 127.5, 127.56, 127.59, 128.0, 128.3, 130.2, 130.7, 132.1, 132.2, 139.8, 142.9, 144.2, 146.7, 147.1 ppm; HRMS (EI) *m/z* calcd for $C_{25}H_{19}BrO$: 414.0619, found: 414.0604. **2-[hydroxyl(diphenyl)methyl]biphenyl (12)**: ^{1}H NMR (400 MHz, $CDCl_3$) δ 2.95 (s, 1H), 6.72–6.83 (m, 3H), 7.08–7.30 ppm (m, 16H); ^{13}C NMR (100 MHz, $CDCl_3$) δ 83.5, 126.2, 126.7, 127.0, 127.2, 127.6, 127.8, 127.9, 129.3, 129.9, 132.2, 140.7, 141.7, 144.9, 147.3 ppm; HRMS (EI) *m/z* calcd for $C_{25}H_{20}O$: 336.1514, found: 336.1516.

Typical Procedure for Sequential Introduction of Two Electrophiles to Dibromobiaryls.

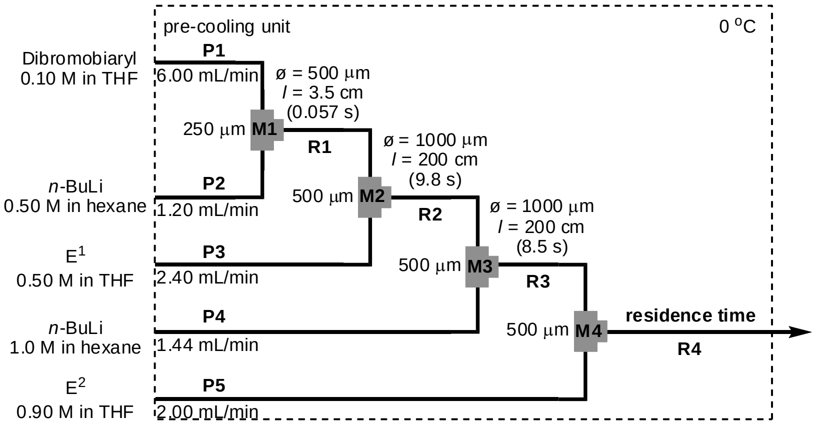

A microflow system consisting of four T-shaped micromixers (**M1, M2, M3** and **M4**), four microtube reactors (**R1**, **R2, R3** and **R4**) and five microtube pre-cooling units [**P1** (inner diameter ø = 1000 μm, length *l* = 100 cm), **P2** (ø = 1000 μm, *l* = 50 cm), **P3** (ø = 1000 μm, *l* = 100 cm), **P4** (ø = 1000 μm, *l* = 50 cm) and **P5** (ø = 1000 μm, *l* = 100 cm)] was used. The whole microflow system was dipped in a cooling bath (0 °C). A solution of a dibromobiaryl (0.10 M) in THF (flow rate = 6.00 mL·min^{-1}) and a solution of *n*-BuLi (0.50 M) in hexane (flow rate = 1.20 mL·min^{-1}) were introduced to **M1** (ø = 250 μm). The resulting solution was passed through **R1** (ø = 500 μm, *l* = 3.5 cm) and was mixed with a solution of a first electrophile (E^1: Electrophile-1) (0.50 M) in THF (flow rate = 2.40 mL·min^{-1}) in **M2** (ø = 500 μm). The resulting solution was passed through **R2** (ø = 1000 μm, *l* = 200 cm) and was introduced to **M3** (ø = 500 μm) where the solution was mixed with a solution of *n*-BuLi (0.50 M) in hexane (flow rate = 1.44 mL·min^{-1}). The resulting solution was passed through **R3** (ø = 1000 μm, *l* = 200 cm) and was introduced to **M4** (ø = 500 μm) where the solution was mixed with a solution of a second electrophile (E^2: Electrophile-2) (0.90 M) in THF (flow rate = 2.00 mL·min^{-1}). The resulting solution was passed through **R4**. After a steady state was reached, the product solution was collected for 60 s and was treated with H_2O to quench the reaction. 2,2'-Dibromobiphenyl (**1**), 4,4'-dibromobiphenyl (**17**), 2,7-dibromo-9,9-dioctylfluorene (**28**), 2,2'-dibromo-1,1'-binaphthyl (**36**) were used as dibromobiaryls. Iodomethane, chlorotrimethylsilane, benzaldehyde, benzophenone, and methyl chlorocarbonate were used as electrophiles.

When iodomethane was used for a first electrophile and benzaldehyde, benzophenone and methyl chlorocarbonate were used for a second electrophile, reactions were carried out under the following conditions.

R4: ø = 1000 μm, L = 50 cm (1.8 s).

When iodomethane was used for a first electrophile and chlorotrimethylsilane were used for

a second electrophile, reactions were carried out under the following conditions.

R4: ø = 1000 μm, L = 200 cm (7.2 s).

Sequential Introduction of Iodomethane (E^1) and Benzaldehyde (E^2) to 2,2'-Dibromobiphenyl (1).

The reaction mixture was washed with H_2O and was extracted with diethyl ether (3 x 20 mL). The combined organic extract was dried over Na_2SO_4, and the solvent was removed. The crude product was purified with silica gel column chromatography (hexane/ethylacetate 20:1) to obtain 2-methyl-2'-[hydroxyl(phenyl)methyl]biphenyl (**13**) [115.8 mg, 70%, diastereomer ratio = 55/45(NMR)]. **2-Methyl-2'-[hydroxyl(phenyl)methyl]biphenyl (13)**: ^{1}H NMR (400 MHz, $CDCl_3$) δ 1.60 (s) and 2.15 (s) (total 3H, two diastereomers), 1.96–2.05 (m, 1H), 5.57–5.61 (m) and 5.70–5.74 (m) (total 1H, two diastereomers), 6.80–7.47 (m, 12H), 7.61–7.73 ppm (m, 1H); ^{13}C NMR (100 MHz, $CDCl_3$, a mixture of two diasteromers, some signals overlapped) δ 19.6, 20.1, 72.9, 73.0, 125.35, 125.37, 126.1, 126.4, 126.5, 126.8, 127.0, 127.16, 127.23, 127.3, 127.46, 127.52, 127.6, 127.8, 128.0, 128.1, 129.3, 129.5, 129.73, 129.74, 130.1, 135.8, 136.6, 140.0, 140.16, 140.19, 140.23, 141.3, 142.9, 143.8 ppm; HRMS (EI) *m/z* calcd for $C_{20}H_{18}O$: 274.1358, found: 274.1353.

Sequential Introduction of Iodomethane (E^1) and Chlorotrimethylsilane (E^2) to 2,2'-Dibromobiphenyl (1).

GC analysis of the reaction mixture indicated that 2-methyl-2'-trimethylsilylbiphenyl (**14**) (GC t_R 21.1 min) was produced in 82% yield. **2-Methyl-2'-trimethylsilylbiphenyl (14)**: ^{1}H NMR (400 MHz, $CDCl_3$) δ −0.05 (s, 9H), 2.05 (s, 3H), 7.09–7.40 (m, 7H), 7.61–7.65 ppm (m, 1H); ^{13}C NMR (100 MHz, $CDCl_3$) δ 0.0, 20.4, 124.8, 126.1, 127.4, 128.4, 129.4, 130.2, 134.7,

136.1, 138.4, 143.4, 148.1 ppm; HRMS (EI) *m/z* calcd for $C_{16}H_{20}Si$: 240.1334, found: 240.1338.

Sequential Introduction of Iodomethane (E^1) and Methyl Chlorocarbonate (E^2) to 2,2'-Dibromobiphenyl (1).

GC analysis of the reaction mixture indicated that 2-methyl-2'-methoxycarbonylbiphenyl (**15**) (GC t_R 21.5 min) was produced in 76% yield. **2-Methyl-2'-methoxycarbonylbiphenyl (15)**: ^{1}H NMR (400 MHz, $CDCl_3$) δ 2,07 (s, 3H), 3.60 (s, 3H), 7.07–7.10 (m, 1H), 7.16–7.28 (m, 4H), 7.41 (td, J = 7.6, 1.6 Hz, 1H), 7.52 (td, J = 7.6, 1.6 Hz, 1H), 7.93–7.98 ppm (m, 1H); ^{13}C NMR (100 MHz, $CDCl_3$) δ19.9, 51.8, 125.2, 127.1, 127.2, 128.4, 129.4, 129.9, 130.3, 130.9, 131.5, 135.2, 141.4, 142.8, 167.8 ppm; HRMS (EI) *m/z* calcd for $C_{15}H_{14}O_2$: 226.0994, found: 226.0993.

Sequential Introduction of Benzaldehyde (E^1) and Methyl Chlorocarbonate (E^2) to 2,2'-Dibromobiphenyl (1).

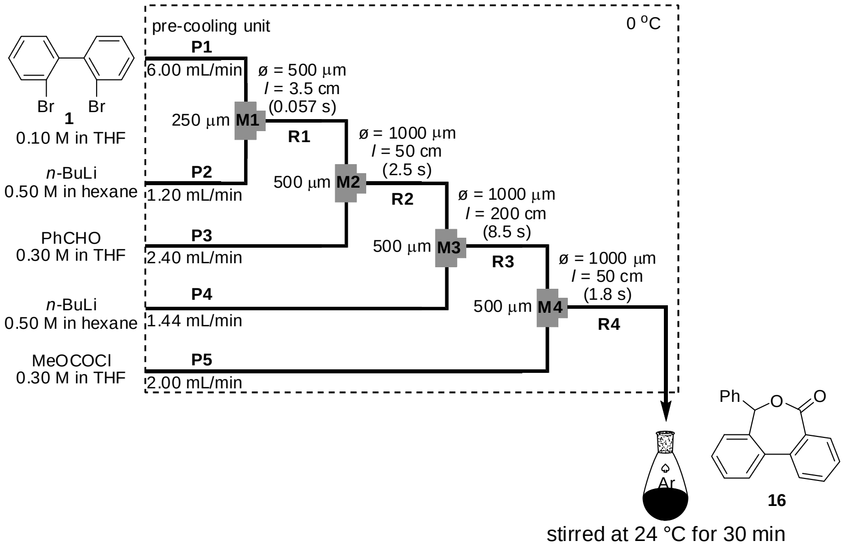

A microflow system consisting of four T-shaped micromixers (**M1, M2, M3** and **M4**), four microtube reactors (**R1**, **R2, R3** and **R4**) and five microtube pre-cooling units [**P1** (inner

diameter ø = 1000 μm, length l = 100 cm), **P2** (ø = 1000 μm, l = 50 cm), **P3** (ø = 1000 μm, l = 100 cm), **P4** (ø = 1000 μm, l = 50 cm) and **P5** (ø = 1000 μm, l = 100 cm)] was used. The whole microflow system was dipped in a cooling bath (0 °C). A solution of 2,2'-dibromobiphenyl (0.10 M) in THF (flow rate = 6.00 mL·min^{-1}) and a solution of *n*-BuLi (0.50 M) in hexane (flow rate = 1.20 mL·min^{-1}) were introduced to **M1** (ø = 250 μm). The resulting solution was passed through **R1** (ø = 500 μm, l = 3.5 cm) and was mixed with a solution of benzaldehyde (0.30 M) in THF (flow rate = 2.40 mL·min^{-1}) in **M2** (ø = 500 μm). The resulting solution was passed through **R2** (ø = 1000 μm, l = 50 cm) and was introduced to **M3** (ø = 500 μm) where the solution was mixed with a solution of *n*-BuLi (0.50 M) in hexane (flow rate = 1.44 mL·min^{-1}). The resulting solution was passed through **R3** (ø = 1000 μm, l = 200 cm) and was introduced to **M4** (ø = 500 μm) where the solution was mixed with a solution of methyl chlorocarbonate (0.30 M) in THF (flow rate = 2.00 mL·min^{-1}). The resulting solution was passed through **R4** (ø = 1000 μm, l = 50 cm). After a steady state was reached, the product solution was collected for 60 s and was stirred for 30 min. The reaction mixture was treated with H_2O to quench the reaction and was analyzed by GC. GC analysis of the reaction mixture indicated that 7-phenyl-5,7-dihydrodibenz[c,e]oxepin-5-one (**16**) (GC t_R 31.2 min) was produced in 75% yield. **7-Phenyl-5,7-dihydrodibenz[c,e]oxepin-5-one (16)**: ^{1}H NMR (400 MHz, $CDCl_3$) δ 6.24 (s, 1H), 6.79 (d, J = 8.0 Hz, 1H), 7.22–7.78 (m, 11H), 8.02 ppm (d, J = 7.6 Hz, 1H); ^{13}C NMR (100 MHz, $CDCl_3$) δ 79.0, 127.0, 127.4, 128.40, 128.45, 128.51, 128.54, 128.8, 128.9, 129.5, 130.7, 131.4, 132.7, 135.7, 137.3, 138.45, 138.55, 169.4 ppm; HRMS (EI) *m/z* calcd for $C_{20}H_{14}O_2$: 286.0994, found: 286.0990.

The Br-Li Exchange Reaction of 4,4'-Dibromobiphenyl (17) Followed by the Reaction with Methanol in a Macrobatch System.

The results are summarized in Table S-4.

Table S-4. The Br-Li exchange reaction of 4,4'-dibromobiphenyl (**17**) followed by the reaction with methanol in a macrobatch system.

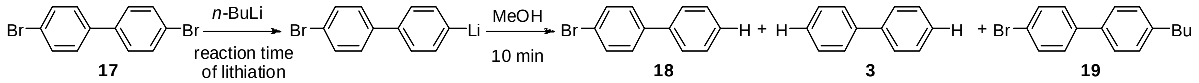

reaction temperature (°C)	reaction time of lithiation (min)	**17** conv. (%)	**18** yield (%)	**3** yield (%)	**19** yield (%)
−78	60	95	87	5	0
−48	10	90	49	5	0
−27	10	81	56	5	2
0	10	86	47	6	13
24	10	87	25	2	26

4-Bromo-4'-butylbiphenyl (19): ^{1}H NMR (400 MHz, $CDCl_3$) δ 0.94 (td, *J* = 7.4, 2.4 Hz, 3H), 1.38 (sext., *J* = 7.4 Hz, 2H), 1.63 (quin., *J* = 7.6 Hz, 2H), 2.64 (t., *J* = 7.6 Hz, 2H), 7.21–7.28 (m, 2H), 7.40–7.49 (m, 4H), 7.50–7.56 ppm (m, 2H); ^{13}C NMR (100 MHz, $CDCl_3$) δ 14.0, 22.4, 33.6, 35.3, 121.1, 126.7, 128.5, 129.0, 131.8, 137.3, 140.1, 142.5 ppm; HRMS (EI) *m/z* calcd for $C_{16}H_{17}Br$: 288.0514, found: 288.0513.

Effects of the Residence Time and Temperature in the Br-Li Exchange Reaction of 4,4'-Dibromobiphenyl (17) Followed by the Reaction with Methanol in the Microflow System.

The results are summarized in Table S-5.

Table S-5. Effects on the residence time and bath temperature in the Br-Li exchange reaction of 4,4'-dibromobiphenyl (**17**) followed by the reaction with methanol in the microflow system.

residence time of **R1** (s)	inner diameter of **R1** (μm)	length of **R1** (cm)	bath temperature (°C)	**17** conv. (%)	**18** yield (%)	**3** yield (%)
0.057	500	3.5	−78	30	29	1
			−48	66	63	0
			0	96	88	4
			24	94	85	4
0.23	1000	3.5	−78	50	48	2
			−48	98	90	4
			0	98	88	6
			24	96	88	5
0.82		12.5	−78	93	89	2
			−48	100	91	5
			0	98	87	6
			24	97	88	6
13		200	−78	92	81	7
			−48	100	88	6
			0	97	87	5
			24	95	85	3

The Br-Li Exchange Reaction of 4,4'-Dibromobiphenyl (17) Followed by the Reaction with Iodomethane in a Microflow System.

GC analysis of the reaction mixture indicated that 4-bromo-4'-methylbiphenyl (**20**) (GC t_R 22.3 min) was produced in 85% yield and 4,4'-dimethylbiphenyl (**21**) (GC t_R 20.0 min) was

produced in 4% yield (93% conv.). The spectral data of 4-bromo-4'-methylbiphenyl (**20**) and 4,4'-dimethylbiphenyl (**21**) were identical to those reported in the literature.[1,3]

The Br-Li Exchange Reaction of 4,4'-Dibromobiphenyl (17) Followed by the Reaction with Benzaldehyde in a Microflow System.

The reaction mixture was washed with H_2O and was extracted with diethyl ether (3 x 20 mL). The combined organic extract was dried over Na_2SO_4, and the solvent was removed. The crude product was purified with silica gel column chromatography (hexane/ethylacetate 5:1 to 3:1) to obtain 4-bromo-4'-[hydroxyl(phenyl)methyl]biphenyl (**22**) (169.1 mg, 83%) and 4,4'-di[hydroxyl(phenyl)methyl]biphenyl (**23**) (12.1 mg, 6%). **4-Bromo-4'-[hydroxyl(phenyl)methyl]biphenyl** (**22**): ^{1}H NMR (400 MHz, $CDCl_3$) δ 2.24 (dd, *J* = 3.2, 0.4 Hz, 1H), 5.88 (d, *J* = 3.2 Hz, 1H), 7.23–7.55 ppm (m, 13H); ^{13}C NMR (100 MHz, $CDCl_3$) δ 75.9, 121.4, 126.4, 126.85, 126.91, 127.6, 128.4, 128.5, 131.7, 139.0, 139.5, 143.0, 143.5 ppm; HRMS (EI) *m/z* calcd for $C_{19}H_{15}BrO$: 338.0306, found: 338.0305. **4,4'-Di[hydroxyl(phenyl)methyl]biphenyl** (**23**): ^{1}H NMR (400 MHz, $CDCl_3$) δ 2.21 (d, *J* = 3.6 Hz, 2H), 5.88 (d, *J* = 3.6 Hz, 2H), 7.20–7.45 (m, 14H), 7.50–7.55 ppm (m, 4H); ^{13}C NMR (100 MHz, $CDCl_3$) δ 76.0, 126.4, 126.8, 127.1, 127.5, 128.4, 139.9, 142.7, 143.5 ppm; HRMS (EI) *m/z* calcd for $C_{26}H_{22}O_2$: 366.1620, found: 366.1622.

The Br-Li Exchange Reaction of 4,4'-Dibromobiphenyl (17) Followed by the Reaction with Benzophenone in a Microflow System.

The reaction mixture was washed with H_2O, and was extracted with diethyl ether (3 x 20 mL). The combined organic extract was dried over Na_2SO_4, and the solvent was removed. The crude product was purified with silica gel column chromatography (hexane/ethylacetate 20:1

to 10:1 to 5:1) to obtain 4-bromo-4'-[hydroxyl(diphenyl)methyl]biphenyl (**24**) (209.3 mg, 84%) and 4,4'-bis[hydroxyl(diphenyl)methyl]biphenyl (**25**) (16.1 mg, 5%). **4-Bromo-4'-[hydroxyl(diphenyl)methyl]biphenyl (24)**: ^{1}H NMR (400 MHz, $CDCl_3$) δ 2.80 (s, 1H), 7.23-7.38 (m, 12H), 7.41–7.56 ppm (m, 6H); ^{13}C NMR (100 MHz, $CDCl_3$) δ 81.8, 121.5, 126.2, 127.2, 127.7, 127.8, 128.3, 128.5, 131.7, 138.5, 139.3, 146.1, 146.5 ppm; HRMS (EI) *m/z* calcd for $C_{25}H_{19}OBr$: 414.0619, found: 414.0621. **4,4'-Bis[hydroxyl(diphenyl)methyl]biphenyl (25)**: ^{1}H NMR (400 MHz, $CDCl_3$) δ 2.80–2.84 (m, 2H), 7.23–7.34 (m, 24H), 7.47–7.54 ppm (m, 4H); ^{13}C NMR (100 MHz, $CDCl_3$) δ 81.9, 126.4, 127.2, 127.76, 127.83, 128.2, 139.3, 145.8, 146.6 ppm; HRMS (EI) *m/z* calcd for $C_{38}H_{30}O_2$: 518.2246, found: 518.2242.

Sequential Introduction of Iodomethane (E^1) and Chlorotrimethylsilane (E^2) to 4,4'-Dibromobiphenyl (17).

GC analysis of the reaction mixture indicated that 4-methyl-4'-trimethylsilylbiphenyl (**26**) (GC t_R 23.8 min) was produced in 71% yield. **4-Methyl-4'-trimethylsilylbiphenyl (26)**: ^{1}H NMR (400 MHz, $CDCl_3$) δ 0.33 (s, 9H), 2.42 (s, 3H), 7.25–7.31 (m, 2H), 7.50–7.66 ppm (m, 6H);^{13}C NMR (100 MHz, $CDCl_3$) δ −1.1, 21.1, 126.3, 127.0, 129.5, 133.8, 137.1, 138.3, 138.8, 141.5 ppm; HRMS (EI) *m/z* calcd for $C_{16}H_{20}Si$: 240.1334, found: 240.1334.

Sequential Introduction of Chlorotrimethylsilane (E^1) and Iodomethane (E^2) to 4,4'-Dibromobiphenyl (17).

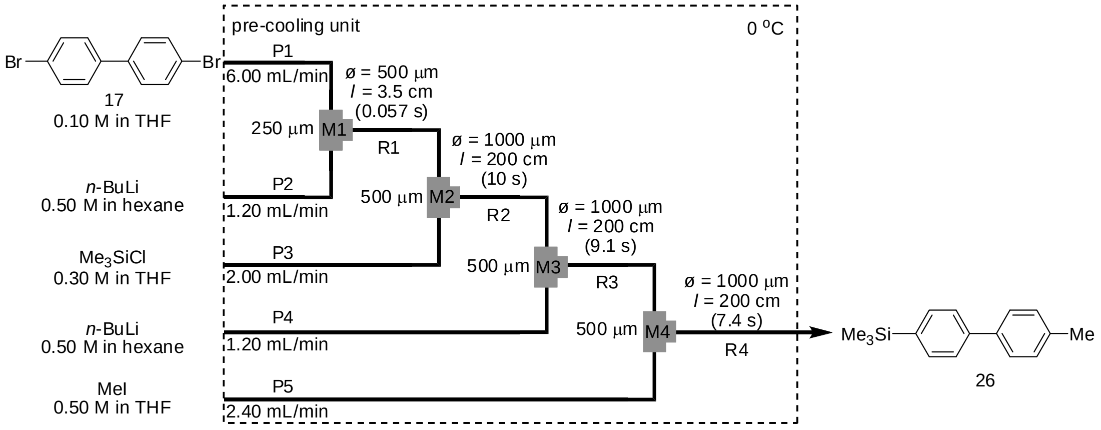

A microflow system consisting of four T-shaped micromixers (**M1, M2, M3** and **M4**), four microtube reactors (**R1**, **R2, R3** and **R4**) and five microtube pre-cooling units [**P1** (inner diameter ø = 1000 µm, length *l* = 100 cm), **P2** (ø = 1000 µm, *l* = 50 cm), **P3** (ø = 1000 µm, *l* = 100 cm), **P4** (ø = 1000 µm, *l* = 50 cm) and **P5** (ø = 1000 µm, *l* = 100 cm)] was used. The whole microflow system was dipped in a cooling bath (0 °C). A solution of a dibromobiaryl (0.10 M) in THF (flow rate = 6.00 mL·min^{-1}) and a solution of *n*-BuLi (0.50 M) in hexane (flow rate = 1.20 mL·min^{-1}) were introduced to **M1** (ø = 250 µm). The resulting solution was passed through **R1** (ø = 500 µm, *l* = 3.5 cm) and was mixed with a solution of chlorotrimethylsilane (0.30 M) in THF (flow rate = 2.00 mL·min^{-1}) in **M2** (ø = 500 µm). The resulting solution was passed through **R2** (ø = 1000 µm, *l* = 200 cm) and was introduced to **M3** (ø = 500 µm) where the solution was mixed with a solution of *n*-BuLi (0.50 M) in hexane (flow rate = 1.20 mL·min^{-1}). The resulting solution was passed through **R3** (ø = 1000 µm, *l* = 200 cm) and was introduced to **M4** (ø = 500 µm) where the solution was mixed with a solution of iodomethane (0.50 M) in THF (flow rate = 2.40 mL·min^{-1}). The resulting solution was passed through **R4** (ø = 1000 µm, *l* = 200 cm). After a steady state was reached, the product solution was collected

for 60 s and was treated with H_2O to quench the reaction. GC analysis of the reaction mixture indicated that 4-methyl-4'-trimethylsilylbiphenyl (**26**) (GC t_R 23.8 min) was produced in 75% yield.

Sequential Introduction of Iodomethane (E^1) and Methyl Chlorocarbonate (E^2) to 4,4'-Dibromobiphenyl (17).

GC analysis of the reaction mixture indicated that 4-methyl-4'-methoxycarbonylbiphenyl (GC t_R 25.0 min) was produced in 56% yield. The spectral data of 4-methyl-4'-methoxycarbonylbiphenyl (**27**) were identical to those reported in the literature.[6]

The Br-Li Exchange Reaction of 2,7-Dibromo-9,9-dioctylfluorene (28) Followed by the Reaction with Methanol in a Macrobatch System.

The results are summarized in Table S-6.

Table S-6. The Br-Li exchange reaction of 2,7-dibromo-9,9-dioctylfluorene (**28**) followed by the reaction with methanol in a macrobatch system.

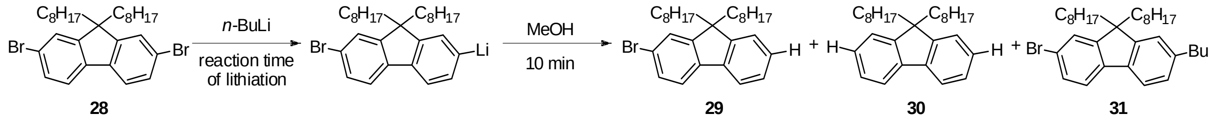

reaction temperature (°C)	reaction time of lithiation (min)	**28** conv. (%)	**29** yield (%)	**30** yield (%)	**31** yield (%)
-78	60	99	89	6	0
0	10	91	54	5	31

2-Bromo-7-butyl-9,9-dioctylfluorene (31): ^{1}H NMR (400 MHz, $CDCl_3$) δ 0.52–0.69 (m, 4H), 0.82 (t, J = 7.0 Hz, 6H), 0.93 (t, J = 7.4 Hz, 3H), 0.98–1.26 (m, 20H), 1.36 (sext., J = 7.4 Hz, 2H), 1.63 (quin., J = 7.6 Hz, 2H), 1.83–1.98 (m, 4H), 7.08–7.15 (m, 2H), 7.38–7.45 (m, 2H), 7.49 (d, J = 7.6 Hz, 1H), 7.55 (d, J = 8.0 Hz, 3H); ^{13}C NMR (100 MHz, $CDCl_3$) δ 14.0, 14.1, 22.3, 22.6, 23.6, 29.1, 29.2, 29.9, 31.8, 33.9, 36.0, 40.2, 55.2, 119.4, 120.3, 120.6, 122.9, 126.0, 127.1, 129.7, 137.6, 140.3, 142.5, 150.4, 152.9 ppm; HRMS (EI) m/z calcd for $C_{16}H_{17}Br$: 288.0514, found: 288.0513.

The Br-Li Exchange Reaction of 2,7-Dibromo-9,9-dioctylfluorene (28) Followed by the Reaction with Methanol in a Microflow System.

GC analysis of the reaction mixture indicated that 2-bromo-9,9-dioctylfluorene (**29**) (GC t_R 35.1 min) and 9,9-dioctylfluorene (**30**) (GC t_R 32.2 min) was produced. The results are summarized in Table S-7.

Table S-7. The Br-Li exchange reaction of 2,7-dibromo-9,9-dioctylfluorene (**28**) followed by the reaction with methanol in the microflow system.

reaction temperature (°C)	**28** conv. (%)	**29** yield (%)	**30** yield (%)
0	99	95	4
24	98	92	5

The Br-Li Exchange Reaction of 2,7-Dibromo-9,9-dioctylfluorene (28) Followed by the Reaction with Iodomethane in a Microflow System.

GC analysis of the reaction mixture indicated that 2-bromo-7-methyl-9,9-dioctylfluorene

(**32**) (GC t_R 35.6 min) was produced in 93% yield and that 2,7-dimethyl-9,9-dioctylfluorene (**33**) (GC t_R 32.6 min) was produced in 3% yield (99% conv.). **2-Bromo-7-methyl-9,9-dioctylfluorene (32)**: ^{1}H NMR (400 MHz, $CDCl_3$) δ 0.48–0.65 (m, 4H), 0.82 (t, J = 7.2 Hz, 6H), 0.92–1.28 (m, 20H), 1.81–2.00 (m, 4H), 2.42 (s, 3H), 7.09–7.15 (m, 2H), 7.38–7.58 ppm (m, 4H); ^{13}C NMR (100 MHz, $CDCl_3$) δ 14.1, 21.9, 22.6, 23.6, 29.16, 29.20, 29.9, 31.8, 40.3, 55.2, 119.4, 120.3, 120.6, 123.5, 126.0, 127.8, 129.7, 137.3, 137.4, 140.2, 150.5, 152.8 ppm; HRMS (EI) *m/z* calcd for $C_{30}H_{43}Br$: 482.2548, found: 482.2549. **2,7-dimethyl-9,9-dioctylfluorene (33)**: ^{1}H NMR (400 MHz, $CDCl_3$) δ 0.56–0.72 (m, 4H), 0.82 (t, J = 7.0 Hz, 6H), 0.96–1.32 (m, 20H), 1.82–2.00 (m, 4H), 2.41 (s, 6H), 7.06–7.15 (m, 4H), 7.48–7.59 ppm (m, 2H); ^{13}C NMR (100 MHz, $CDCl_3$) δ 14.1, 21.8, 22.6, 23.7, 29.2, 29.3, 30.1, 31.8, 40.4, 54.6, 118.9, 123.5, 127.4, 136.1, 138.6, 150.8 ppm; HRMS (EI) *m/z* calcd for $C_{31}H_{46}$: 418.3600, found: 418.3602.

The Br-Li Exchange Reaction of 2,7-Dibromo-9,9-dioctylfluorene (28) Followed by the Reaction with Benzaldehyde in a Microflow System.

The reaction mixture was washed with H_2O and was extracted with diethyl ether (3 x 20 mL). The combined organic extract was dried over Na_2SO_4, and the solvent was removed. The crude product was purified with silica gel column chromatography (hexane/ethylacetate 50:1 to 20:1 {2-bromo-7-[hydroxyl(phenyl)methyl]-9,9-dioctylfluorene (**34**)} and 15:1 to 8:1 {2,7-di[hydroxyl(phenyl)methyl]-9,9-dioctylfluorene (**35**)}) to obtain 2-bromo-7-[(hydroxyl)phenylmethyl]-9,9-dioctylfluorene (**34**) (311.5 mg, 90%) and 2,7-di[(hydroxyl)phenylmethyl]-9,9-dioctylfluorene (**35**) (8.9 mg, 2%). **2-Bromo-7-((hydroxyl)phenylmethyl)-9,9-dioctylfluorene (34)**: ^{1}H NMR (400 MHz, $CDCl_3$) δ 0.56–0.68 (m, 4H), 0.82 (t, J = 7.2 Hz, 3H), 0.83 (t, J = 7.2 Hz, 3H), 0.95-1.38 (m, 20H), 1.78–2.02 (m, 4H), 2.33 (s, 1H), 5.90 (s, 1H), 7.20–7.47 (m, 9H), 7.50 (d, J = 8.0 Hz, 1H),

7.59 ppm (d, J = 8.0 Hz, 1H); ^{13}C NMR (100 MHz, $CDCl_3$, some signals overlapped) δ 14.1, 22.6, 23.68 and 23.71, 29.14, 29.15, 29.85 and 29.88, 31.73 and 31.74, 40.1, 55.4, 76.4, 119.7, 120.95, 120.99, 125.5, 126.1, 126.6, 127.5, 128.4, 129.9, 139.5, 139.7, 143.2, 144.0, 150.6, 153.1 ppm; HRMS (EI) *m/z* calcd for $C_{36}H_{47}BrO$: 574.2810, found: 574.2786. **2,7-Di((hydroxyl)phenylmethyl)-9,9-dioctylfluorene (35)**: ^{1}H NMR (400 MHz, $CDCl_3$) δ 0.45–0.58 (m, 4H), 0.758 (t, J = 7.0 Hz, 3H), 0.761 (t, J = 7.0 Hz, 3H), 0.82-1.30 (m, 20H), 1.78–1.96 (m, 4H), 2.21–2.30 (m, 2H), 5.92 (s, 2H), 7.18-7.42 (m, 14H), 7.58–7.67 ppm (m, 2H); ^{13}C NMR (100 MHz, $CDCl_3$) δ 14.1, 22.6, 23.8, 29.19, 29.21, 29.9, 31.8, 40.1, 55.1, 76.5, 119.6, 121.0, 125.4, 126.6, 127.5, 128.4, 140.3, 142.6, 144.1, 151.3 ppm; HRMS (EI) *m/z* calcd for $C_{43}H_{54}O_2$: 602.4124, found: 602.4128.

The Br-Li Exchange Reaction of 2,2'-Dibromo-1,1'-binaphthyl (36) followed by the Reaction with Methanol in a Macrobatch System.

The results are summarized in Table S-8.

Table S-8. The Br-Li exchange reaction of 2,2'-dibromo-1,1'-binaphthyl (**36**) followed by the reaction with methanol in a macrobatch system.

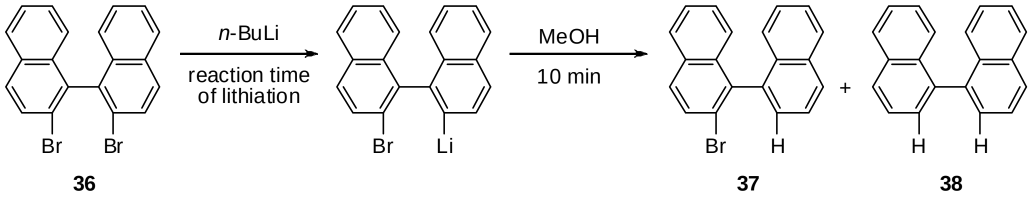

reaction temperature (°C)	reaction time of lithiation (min)	**36** conv. (%)	**37** yield (%)	**38** yield (%)
−78	60	100	90	10
0	10	100	86	13

The Br-Li Exchange Reaction of 2,2'-Dibromo-1,1'-binaphthyl (36) Followed by the Reaction with Methanol in a Microflow System.

GC analysis of the reaction mixture indicated that 2-bromo-1,1'-binaphthyl (**37**) (GC t_R 30.4 min) and that 1,1'-binaphthyl (**38**) (GC t_R 28.5 min) was produced. The results are summarized in Table S-9.

Table S-9. The Br-Li exchange reaction of 2,2'-dibromo-1,1'-binaphthyl (**36**) followed by the reaction with methanol in the microflow system.

reaction temperature (°C)	**36** conv. (%)	**37** yield (%)	**38** yield (%)
0	94	93	1
24	94	92	2

The Br-Li Exchange Reaction of 2,2'-Dibromo-1,1'-binaphthyl (36) Followed by the Reaction with Iodomethane in a Microflow System.

GC analysis of the reaction mixture indicated that 2-bromo-2'-methyl-1,1'-binaphthyl (**39**) (GC t_R 30.3 min) was produced in 85% yield and that 2,2'-dimethyl-1,1'-binaphthyl (**40**) (GC t_R 28.6 min) was produced in a trace amount (95% conv.). **2-Bromo-2'-methyl-1,1'-binaphthyl (39)**: ^{1}H NMR (400 MHz, $CDCl_3$) δ 2.08 (s, 3H), 7.02 (d, J = 8.4 Hz, 1H), 7.09 (d, J = 8.4 Hz, 1H), 7.20–7.30 (m, 2H), 7.37–7.54 (m, 3H), 7.78–7.95 ppm (m, 5H); ^{13}C NMR (100 MHz, $CDCl_3$, some signals overlapped) δ 20.0, 122.8, 125.0, 125.3, 126.1, 126.2, 126.3, 127.1, 128.0, 128.1, 128.6, 129.1, 130.0, 132.0, 132.2, 132.4, 133.7, 134.4, 135.0, 137.3 ppm; HRMS (EI) *m/z* calcd for $C_{21}H_{15}Br$: 346.0357, found: 346.0360. The spectral data of 2,2'-dimethyl-1,1'-binaphthyl (**40**) were identical to those reported in the literature.[4]

The Br-Li Exchange Reaction of 2,2'-Dibromo-1,1'-binaphthyl (36) Followed by the Reaction with Benzaldehyde in a Microflow System.

The reaction mixture washed with H_2O and was extracted with diethyl ether (3 x 20 mL). The combined organic extract was dried over Na_2SO_4, and the solvent was removed. The crude product was purified with silica gel column chromatography (hexane/ethylacetate 20:1) to obtain 2-bromo-2'-[hydroxyl(phenyl)methyl]-1,1'-binaphthyl (**41**) as two diastereomers [216.2 mg, 82%, diastereomer ratio = 60/40 (NMR)], and 2-[hydroxyl(phenyl)methyl]-1,1'-binaphthyl (**42**) was produced in a trace amount as a mixture of two diastereomers [diastereomer ratio = 58/42 (NMR)] (95% conv.). **2-Bromo-2'-[hydroxyl(phenyl)methyl]-1,1'-binaphthyl (41)**: The major diastereomer (128.2 mg, 49%): ^{1}H NMR (400 MHz, $CDCl_3$) δ 1.83–1.92 (m, 1H), 5.43–5.47 (m, 1H), 7.12–7.39 (m, 9H), 7.44–7.57 (m, 2H), 7.66–7.72 (m, 1H), 7.75–7.82 (m, 1H), 7.84–8.03 ppm (m, 4H); ^{13}C NMR (100 MHz, $CDCl_3$) δ 73.1, 124.0, 124.9, 125.8, 126.0, 126.2, 126.5, 126.6, 126.7, 127.2,

127.7, 128.10, 128.13, 128.4, 129.2, 129.7, 130.1, 131.7, 132.4, 133.1, 134.3, 134.9, 135.8, 139.5, 142.5 ppm; HRMS (EI) *m/z* calcd for $C_{27}H_{19}BrO$: 438.0619, found: 438.0618. The minor diastereomer (88.0 mg, 33%): ^{1}H NMR (400 MHz, $CDCl_3$) δ 2.50 (d, *J* = 2.0 Hz, 1H), 5.55 (s, 1H), 6.91 (d, *J* = 8.4 Hz, 1H), 6.94–7.08 (m, 6H), 7.10 (dd, *J* = 7.8, 7.8 Hz, 1H), 7.15-7.25 (m, 1H),7.36–7.45 (m, 2H), 7.75 (d, *J* = 8.8 Hz, 1H), 7.71–7.90 (m, 4H), 7.96 (d, *J* = 8.8 Hz, 1H); ^{13}C NMR (100 MHz, $CDCl_3$, some signals overlapped) δ 73.0, 122.7, 125.0, 126.0, 126.17, 126.18, 126.4, 126.6, 126.7, 126.9, 127.0, 127.9, 128.0, 129.3, 129.79, 129.83, 131.8, 132.3, 133.0, 134.3, 134.7, 136.1, 139.8, 142.4 ppm; HRMS (EI) *m/z* calcd for $C_{27}H_{19}BrO$: 438.0619, found: 438.0618. **2-[Hydroxyl(phenyl)methyl]-1,1'-binaphthyl (42)**: ^{1}H NMR (400 MHz, $CDCl_3$) δ 1.98-2.16 (m, 1H), 5.51–5.55 (m) and 5.63–5.68 (m) (total 1H, two diastereomers), 6.93–8.02 ppm (m, 18H); ^{13}C NMR (100 MHz, $CDCl_3$, a mixture of two diasteromers, some signals overlapped) δ 72.92, 72.94, 124.5, 124.6, 125.3, 125.5, 125.7, 125.9, 126.0, 126.1, 126.2, 126.48, 126.50, 126.6, 126.8, 126.9, 127.07, 127.15, 127.76, 127.79, 127.85, 127.9, 128.09, 128.13, 128.2, 128.4, 128.6, 128.7, 129.0, 132.7, 132.89, 132.94, 133.04, 133.06, 133.10, 133.5, 133.7, 135.4, 135.7, 136.0, 136.2, 139.68, 139.71, 143.0, 143.6 ppm; HRMS (EI) *m/z* calcd for $C_{27}H_{20}O$: 360.1514, found: 360.1514.

The Br-Li Exchange Reaction of 2,2'-Dibromobibenzyl (43) Followed by the Reaction with Methanol in a Macrobatch System.

The results are summarized in Table S-10.

Table S-10. The Br-Li exchange reaction of 2,2'-dibromobibenzyl (**43**) followed by the reaction with methanol in a macrobatch system.

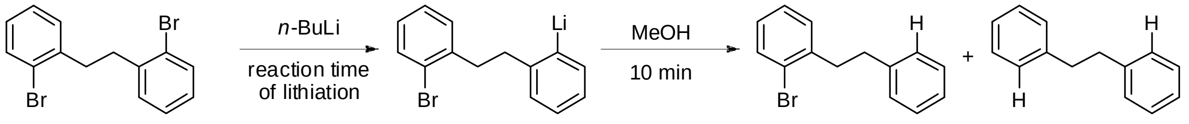

reaction temperature (°C)	reaction time of lithiation (min)	**43** conv. (%)	**44** yield (%)	**45** yield (%)
−78	60	90	85	5
0	10	75	27	15

The Br-Li Exchange Reaction of 2,2'-Dibromobibenzyl (43) followed by the Reaction with Methanol in a Microflow System.

GC analysis of the reaction mixture indicated that 2-bromobibenzyl (**44**) (GC t_R 22.8 min) and that bibenzyl (**45**) (GC t_R 19.5 min) was produced. The results are summarized in Table S-11.

Table S-11. The Br-Li exchange reaction of 2,2'-dibromobibenzyl (**43**) followed by the reaction with methanol in the microflow system.

reaction temperature (°C)	**43** conv. (%)	**44** yield (%)	**45** yield (%)
0	92	80	10
24	90	77	11

The Br-Li Exchange Reaction of 2,2'-Dibromobibenzyl (43) Followed by the Reaction with Iodomethane in a Microflow System.

GC analysis of the reaction mixture indicated that 2-bromo-2'-methylbibenzyl (**46**) (GC t_R 24.0 min) was produced in 81% yield and that 2,2'-dimethylbibenzyl (**47**) (GC t_R 22.1 min) was produced in 4% yield (85% conv.). **2-Bromo-2'-methylbibenzyl (46)**: ^{1}H NMR (400 MHz, $CDCl_3$) δ 2.34 (s, 3H), 2.81–3.04 (m, 4H), 7.02–7.30 (m, 7H), 7.52–7.60 ppm (m, 1H); ^{13}C NMR (100 MHz, $CDCl_3$) δ 19.6, 34.0, 37.5, 124.7, 126.3, 126.5, 127.8, 128.0, 129.3, 130.5, 130.8, 133.1, 136.4, 139.9, 141.4 ppm; HRMS (FAB) *m/z* calcd for $C_{15}H_{15}Br$: 274.0357, found: 274.0355. The spectrum data of 2,2'-dimethylbibenzyl (**47**) were identical to those reported in the literature.[5]

The Br-Li Exchange Reaction of 2,2'-Dibromobibenzyl (43) Followed by the Reaction with Benzaldehyde in a Microflow System.

The reaction mixture was washed with H_2O and was extracted with diethyl ether (3 x 20 mL). The combined organic extract was dried over Na_2SO_4, and the solvent was removed. The crude product was purified with silica gel column chromatography (hexane/ethylacetate 10:1 to 5:1) to obtain 2-bromo-2'-[hydroxyl(phenyl)methyl]bibenzyl (**48**) (144.7 mg, 66%) and 2,2'-di[hydroxyl(phenyl)methyl]bibenzyl (**49**) (17.2 mg, 7%). **2-Bromo-2'-[hydroxyl(phenyl)methyl]bibenzyl (48)**: ^{1}H NMR (400 MHz, $CDCl_3$) δ 2.15 (d, *J* = 3.6 Hz, 1H), 2.79–3.03 (m, 4H), 6.04 (d, *J* = 3.6 Hz, 1H), 6.92–7.06 (m, 2H), 7.11–7.33 (m, 9H), 7.38–7.45 (m, 1H), 7.47–7.54 ppm (m, 1H); ^{13}C NMR (100 MHz, $CDCl_3$, some of the ^{13}C NMR signals were the same places) δ 32.5, 38.2, 72.7, 124.3, 126.5, 126.9, 127.0, 127.36, 127.44, 127.8, 128.4, 129.8, 130.6, 132.8, 138.8, 140.7, 141.1, 143.4 ppm; HRMS (EI) *m/z* calcd for $C_{21}H_{19}OBr$: 366.0619, found: 366.0616. **2,2'-Di[hydroxyl(phenyl)methyl]bibenzyl (49)**: ^{1}H NMR (400 MHz, $CDCl_3$) δ 2.13 (s, 4H), 2.78–2.99 (m, 8H), 5.81 (s, 2H), 5.92 (s, 2H),

7.00–7.38 ppm (m, 36H); ^{13}C NMR (100 MHz, $CDCl_3$) δ 34.1, 34.2, 72.6, 72.7, 126.5, 126.61, 126.62, 126.8, 127.3, 127.4, 127.46, 127.54, 127.8, 127.9, 128.35, 128.39, 129.9, 130.0, 139.1, 139.2, 141.2, 141.5, 143.23, 143.25 ppm; HRMS (EI) *m/z* calcd for $C_{28}H_{26}O_2$ -H: 393.1855, found: 393.1843.

Sequential Introduction of Iodomethane (E^1) and Methyl Chlorocarbonate (E^2) to 2,7-Dibromo-9,9-dioctylfluorene (28).

GC analysis of the reaction mixture indicated that 2-methyl-7-methoxycarbonyl-9,9-dioctylfluorene (**50**) (GC t_R 37.7 min) was produced in 51% yield. **2-Methyl-7-methoxycarbonyl-9,9-dioctylfluorene (50)**: ^{1}H NMR (400 MHz, $CDCl_3$) δ 0.51–0.68 (m, 4H), 0.81 (t, *J* = 7.2 Hz, 6H), 0.94–1.25 (m, 20H), 1.89–2.06 (m, 4H), 2.43 (s, 3H), 3.93 (s, 3H), 7.10–7.18 (m, 2H), 7.61 (s, 1H), 7.67 (s, 1H), 7.98–8.03 (m, 1H), 8.03 ppm (dd, *J* = 7.8, 1.4 Hz,1H); ^{13}C NMR (100 MHz, $CDCl_3$, some signals overlapped) δ 14.0, 21.9, 22.5, 23.6, 29.1, 29.9, 31.7, 40.2, 51.9, 55.0, 118.9, 120.3, 123.6, 123.9, 127.8, 128.0, 128.8, 137.3, 138.2, 146.0, 150.6, 152.0, 167.6 ppm; HRMS (EI) *m/z* calcd for $C_{32}H_{46}O_2$: 462.3498, found: 462.3498.

Sequential Introduction of Iodomethane (E^1) and Benzaldehyde (E^2) to 2,2'-Dibromo-1,1'-binaphthyl (36).

The reaction mixture was washed with H_2O and was extracted with diethyl ether (3 x 20 mL). The combined organic extract was dried over Na_2SO_4, and the solvent was removed. The crude product was purified with silica gel column chromatography (hexane/ethylacetate 12:1) to obtain 2-methyl-2'-[hydroxyl(phenyl)methyl]-1,1'-binaphthyl (**51**) as a mixture of two diastereomers [160.0 mg, 71%, diastereomer ratio = 56/44 (NMR)].

2-Methyl-2'-[hydroxyl(phenyl)methyl]-1,1'-binaphthyl (51): The major diastereomer: ^{1}H NMR (400 MHz, $CDCl_3$) δ 2.01–2.04 (m, 1H), 2.19 (s, 3H), 5.51 (d, *J* = 3.6 Hz, 1H), 6.76–6.81 (m, 1H), 6.91–7.12 (m, 7H), 7.17–7.49 (m, 3H), 7.55 (d, *J* = 8.8 Hz, 1H), 7.79 (d, *J* = 8.8 Hz, 1H), 7.84–8.03 ppm (m, 5H); ^{13}C NMR (150 MHz, $CDCl_3$, some signals overlapped) δ 20.5, 73.4, 124.6, 124.9, 125.9, 126.0, 126.2, 126.3, 126.39, 126.42, 127.0, 127.7, 128.0, 128.1, 128.5, 128.6, 132.0, 132.4, 133.1, 133.3, 133.7, 134.7, 135.1, 139.3, 143.1 ppm; HRMS (EI) *m/z* calcd for $C_{28}H_{22}O$: 374.1671, found: 374.1670. The minor diastereomer: ^{1}H NMR (400 MHz, $CDCl_3$) δ 1.61 (s, 3H), 1.96–2.01 (m, 1H), 5.40 (d, *J* = 2.8 Hz, 1H), 6.99–7.32 (m, 9H), 7.39 (d, *J* = 8.0 Hz, 1H) 7.41–7.48 (m, 2H), 7.88–7.99 (m, 5H), 8.02 ppm (d, *J* = 8.8 Hz, 1H); ^{13}C NMR (150 MHz, $CDCl_3$, some signals overlapped) δ 20.0, 73.4, 124.3, 125.1, 125.2, 126.0, 126.4, 126.7, 126.9, 127.3, 128.0, 128.07, 128.12, 128.2, 128.4, 128.7, 132.2, 132.3, 133.0, 133.1, 133.3, 134.6, 135.9, 139.5, 142.8 ppm; HRMS (EI) *m/z* calcd for $C_{28}H_{22}O$: 374.1671, found: 374.1668.

Sequential Introduction of Two Electrophiles to 2,2'-Dibromo-1,1'-binaphthyl (36). The use of Iodomethane and Benzophenone.

The reaction mixture was washed with H_2O and was extracted with diethyl ether (3 x 20 mL). The combined organic extract was dried over Na_2SO_4, and the solvent was removed. The crude product was purified with silica gel column chromatography (hexane/ethylacetate 30:1) to obtain 2-methyl-2'-[hydroxyl(diphenyl)methyl]-1,1'-binaphthyl (**52**) (212.0 mg, 78%). **2-Methyl-2'-[hydroxyl(diphenyl)methyl]-1,1'-binaphthyl (52)**: ^{1}H NMR (400 MHz, $CDCl_3$) δ 1.80 (s, 3H), 2.80–2.86 (m, 1H), 6.80–7.52 (m, 18H), 7.74–7.90 ppm (m, 4H); ^{13}C NMR (100 MHz, $CDCl_3$) δ 20.4, 84.3, 125.1, 125.7, 125.9, 126.1, 126.50, 126.54, 126.6, 126.9, 127.2, 127.5, 127.6, 127.7, 127.78, 127.81, 128.2, 128.4, 128.5, 129.3, 131.8, 132.4,

133.0, 133.4, 134.0, 134.2, 135.8, 142.2, 146.6, 146.7 ppm; HRMS (EI) *m/z* calcd for $C_{34}H_{26}O$: 450.1984, found: 450.1982.

References

1. Miyake, Y.; Wu, M.; Rahman, M. J.; Kuwatani, Y.; Iyoda, M. *J. Org. Chem.* **2006**, *71*, 6110. doi:10.1021/jo0608063
2. van Klink, G. P. M.; de Boer, H. J. R.; Schat, G.; Akkerman, O. S.; Bickelhaupt, F.; Spek, A. L. *Organometallics* **2002**, *21*, 2119. doi:10.1021/om011083a
3. Murphy, S.; Yang, X.; Schuster, G. B. *J. Org. Chem.* **1995**, *60*, 2411. doi:10.1021/jo00113a022
4. Foubelo, F.; Moreno, B.; Soler, T.; Yus, M. *Tetrahedron* **2005**, *61*, 9082. doi:10.1016/j.tet.2005.07.042
5. Aitken, R. A.; Hodgson, P. K. G.; Morrison, J. J.; Oyewale, A. O. *J. Chem. Soc.* **2002**, *Perkin Trans. 1*, 402.
6. Kobayashi, Y.; William, A. D.; Mizojiri, R. *J. Organomet. Chem.* **2002**, *653*, 91. doi:10.1016/S0022-328X(02)01174-9
