# Supplementary material for: Synthesis of unsymmetrically substituted biaryls via sequential lithiation of dibromobiaryls using integrated microflow systems
Source: Beilstein J Org Chem. 2009 Apr 29;5:16. doi: 10.3762/bjoc.5.16 (PMC2686311; doi:10.3762/bjoc.5.16)
Supplement: File 2 — 1H NMR spectra and 13C NMR spectra of compounds 4, 5, 9–16, 19, 22–26, 31–35, 39. [file Beilstein_J_Org_Chem-05-16-s002.pdf]

Synthesis of unsymmetrically substituted biaryls via sequential lithiation of dibromobiaryls using integrated microflow systems

Aiichiro Nagaki, Naofumi Takabayashi, Yutaka Tomida, and Jun-ichi Yoshida*

Address: Department of Synthetic Chemistry and Biological Chemistry, Graduate School of Engineering, Kyoto University, Kyotodaigakukatsura, Nishikyo-ku, Kyoto, 615-8510, Japan

Email: Jun-ichi Yoshida - yoshida@sbchem.kyoto-u.ac.jp

* Corresponding author

Supporting Information

^{1}H NMR spectra and ^{13}C NMR spectra of compounds 4,5,9–16,19,22–26,31–35,39

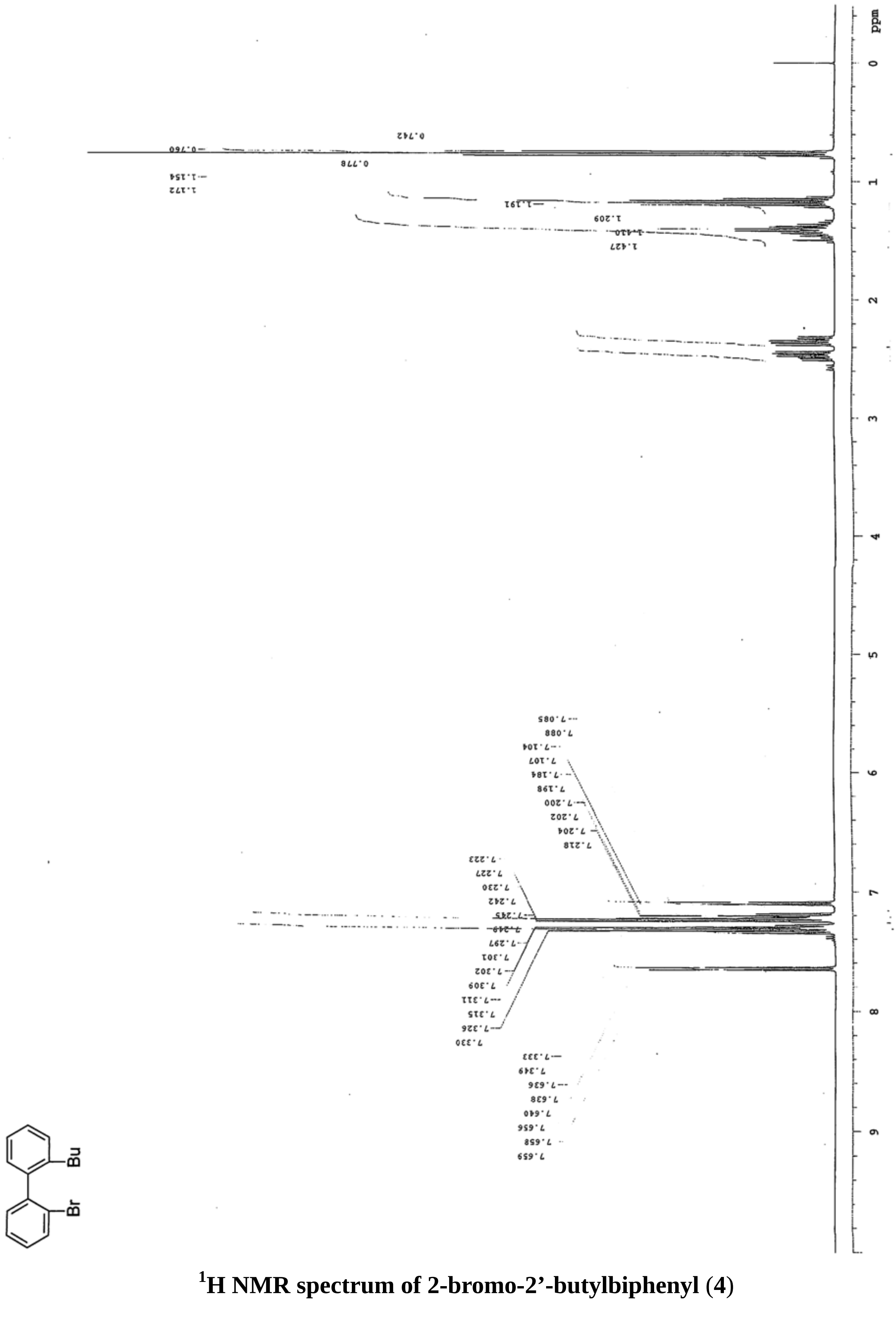

^{1}H NMR spectrum of 2-bromo-2'-butylbiphenyl (4)

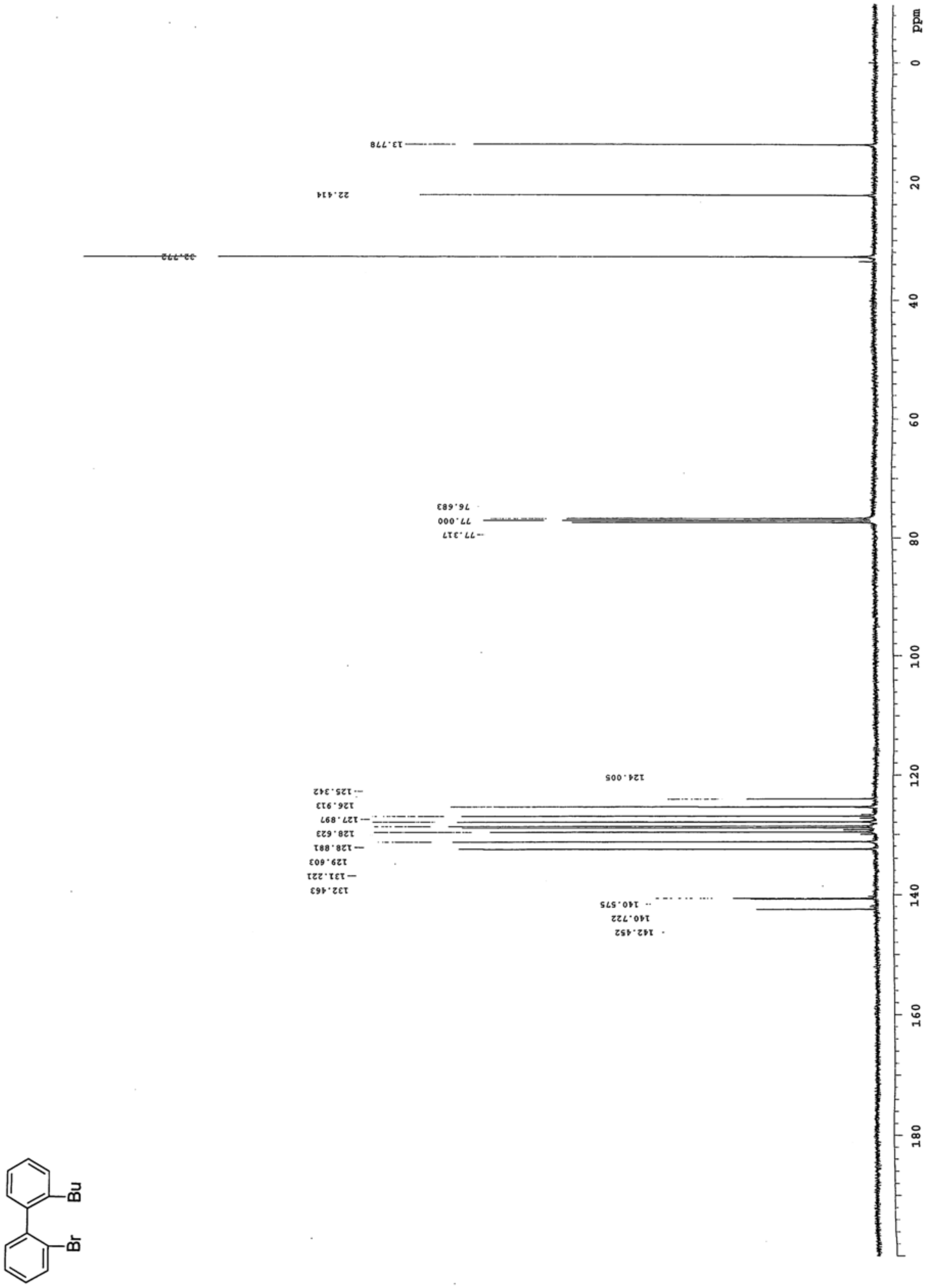

^{13}C NMR spectrum of 2-bromo-2’-butylbiphenyl (4)

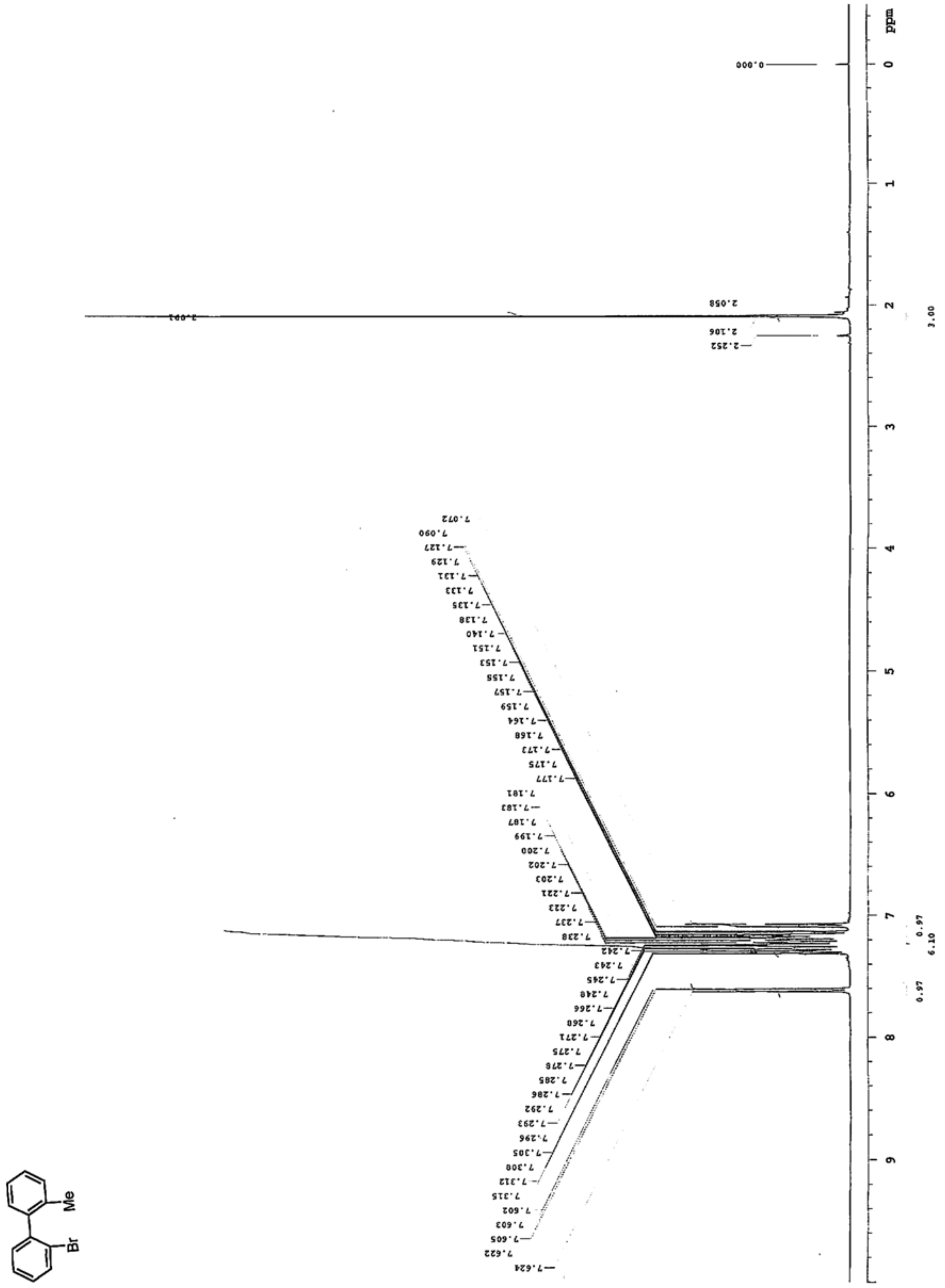

^{1}H NMR spectrum of 2-bromo-2'-methylbiphenyl (5)

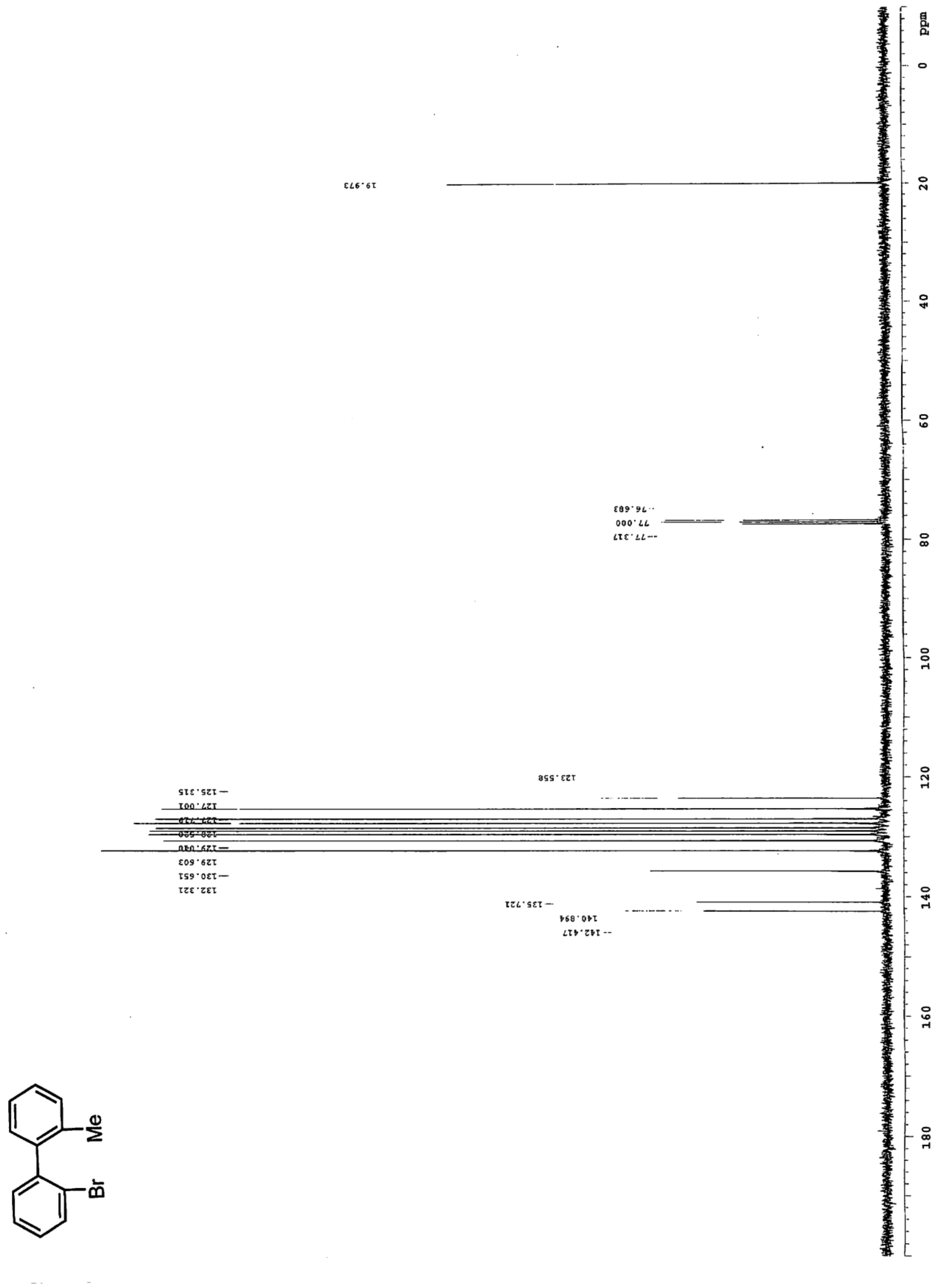

^{13}C NMR spectrum of 2-bromo-2'-methylbiphenyl (5)

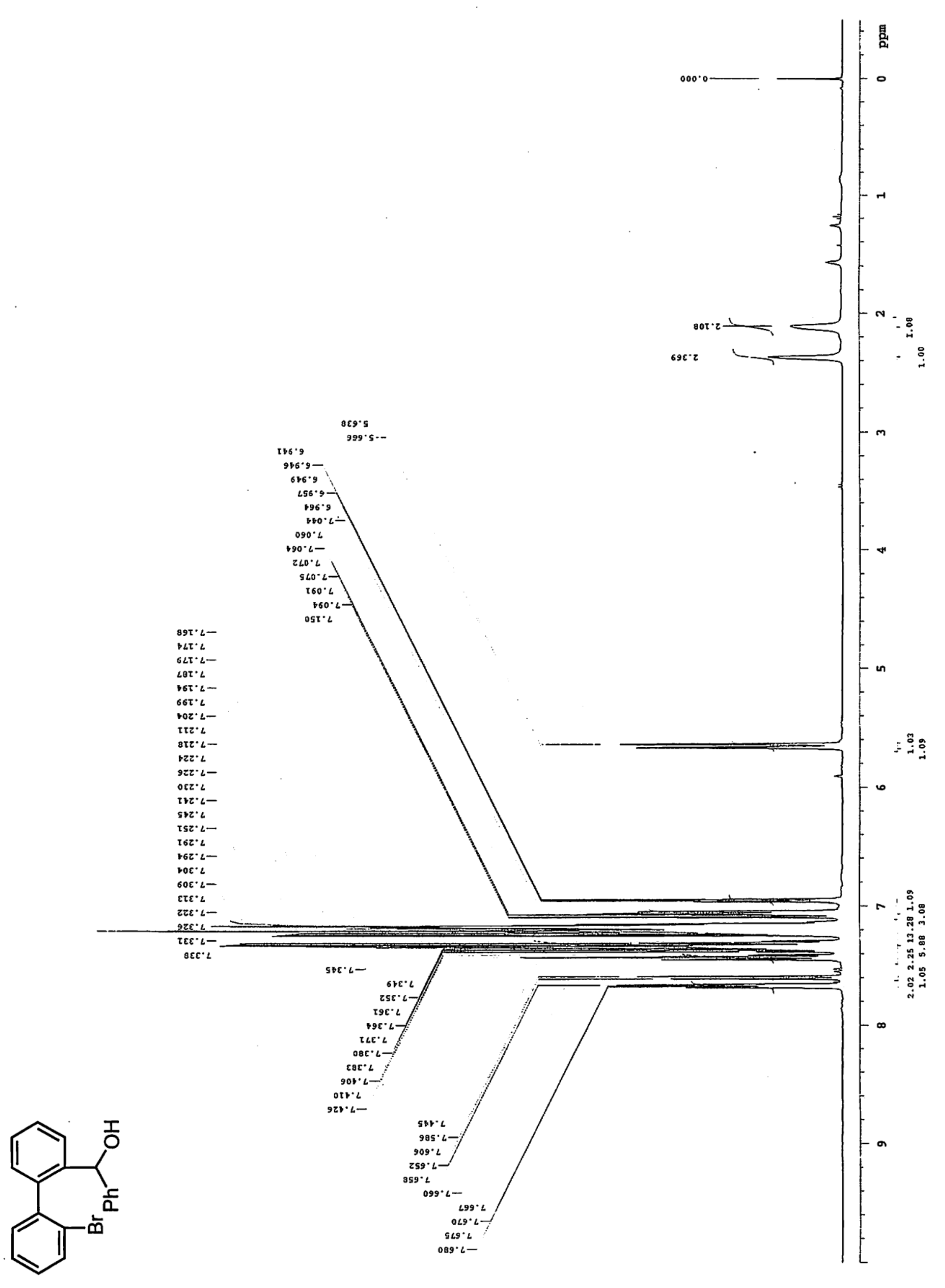

^{1}H NMR spectrum of 2-bromo-2’-[hydroxyl(phenyl)methyl]biphenyl (9)

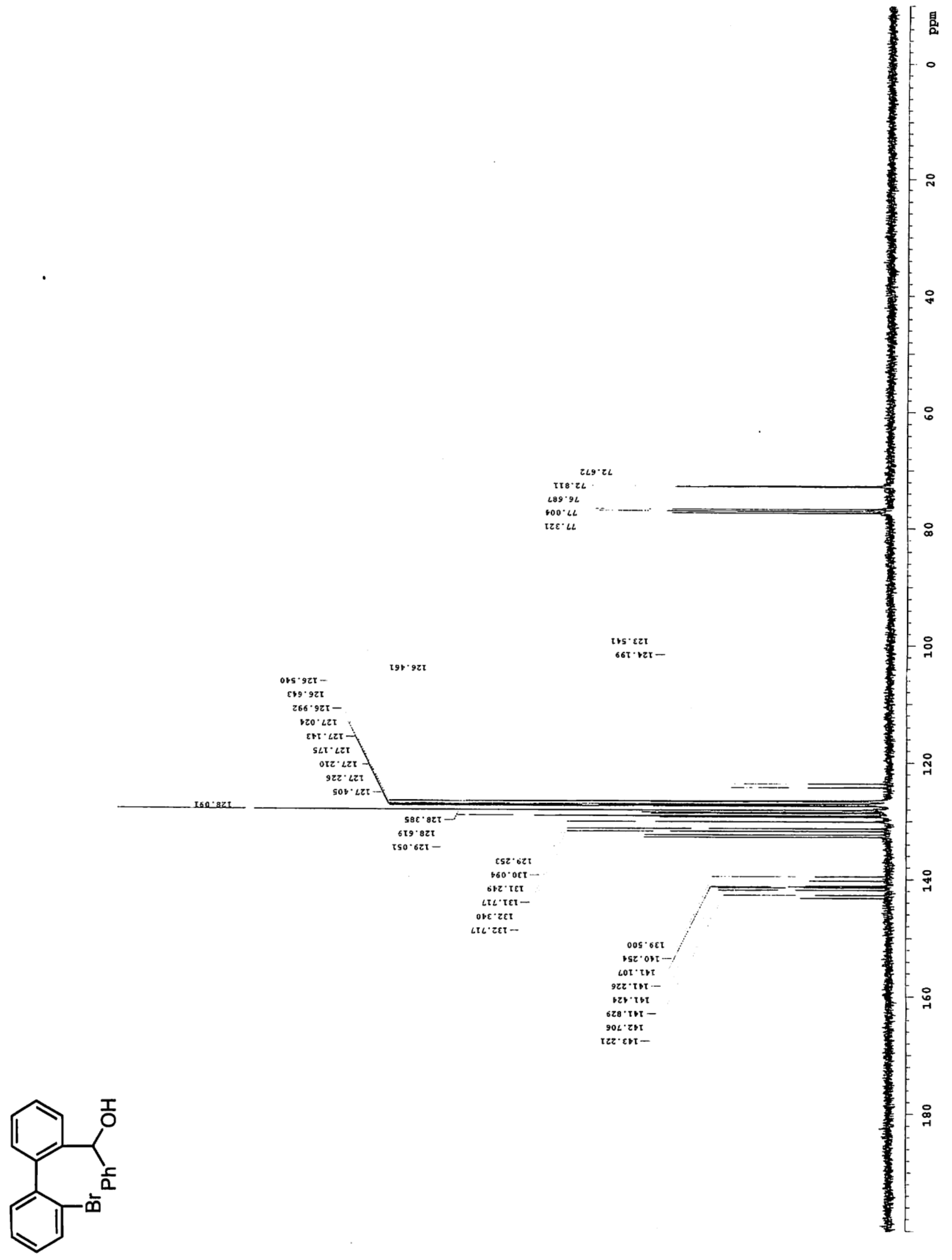

^{13}C NMR spectrum of 2-bromo-2'-[hydroxyl(phenyl)methyl]biphenyl (9)

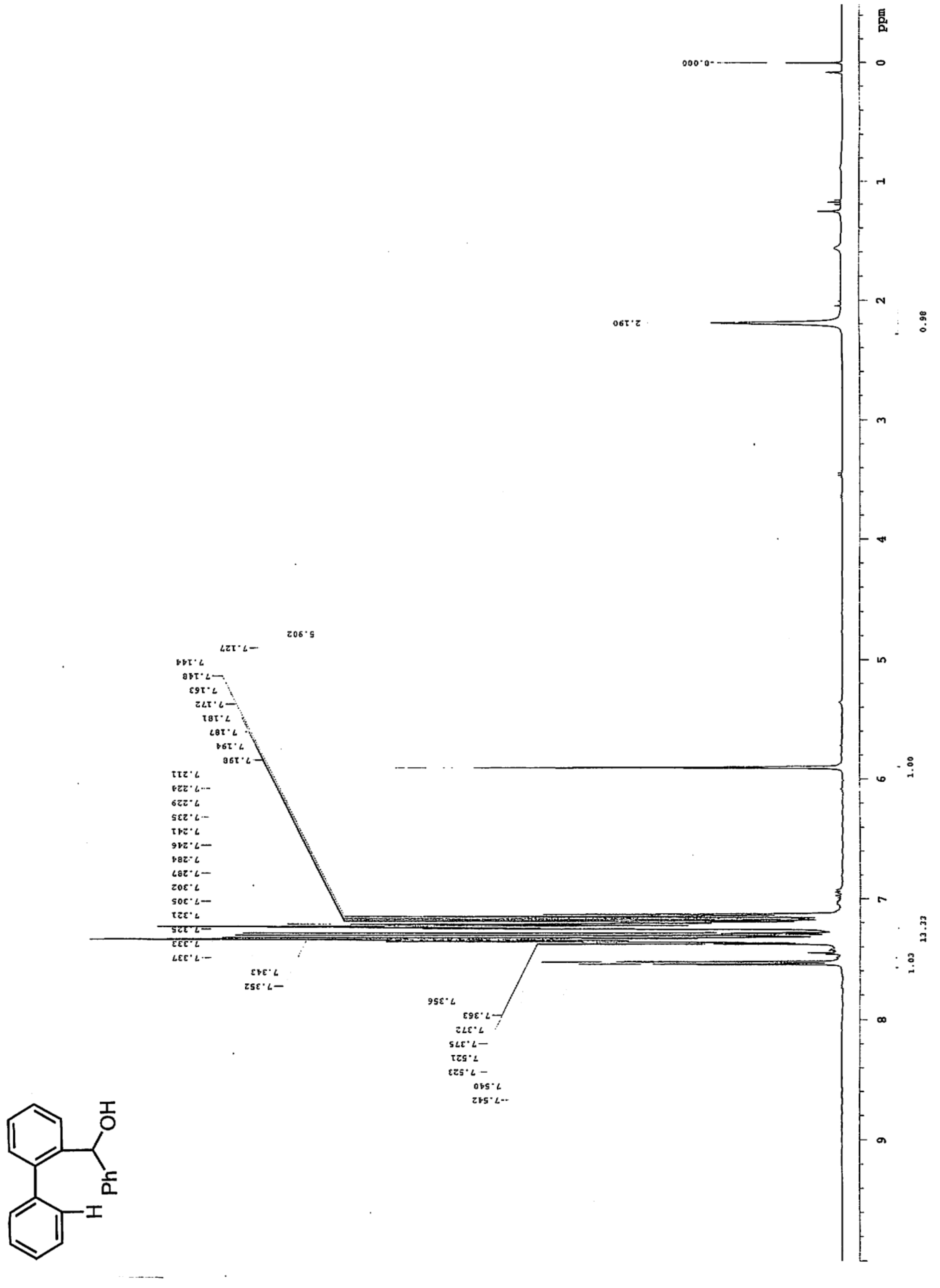

^{1}H NMR spectrum of 2-[hydroxyl(phenyl)methyl]biphenyl (10)

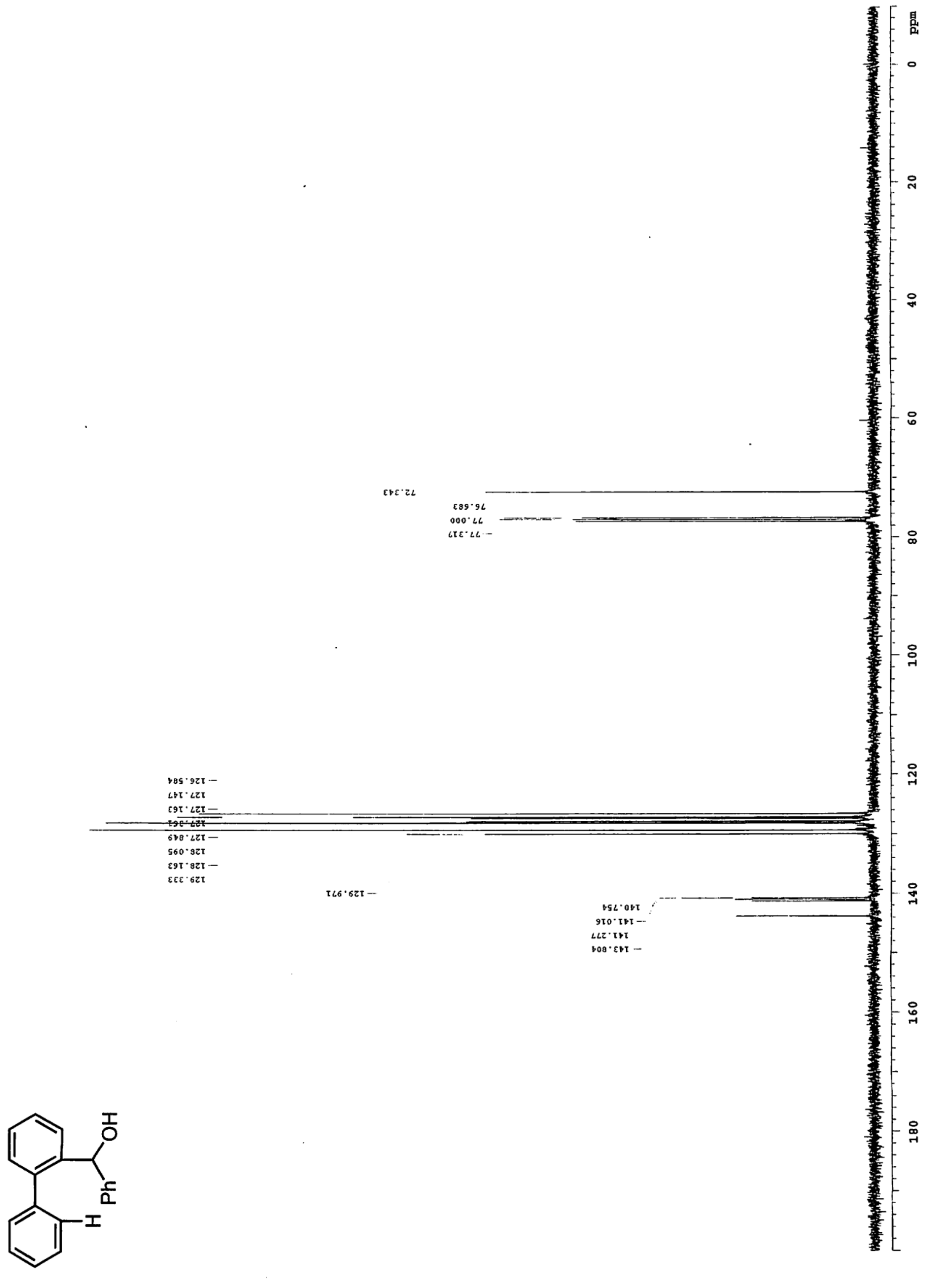

^{13}C NMR spectrum of 2-[hydroxyl(phenyl)methyl]biphenyl (10)

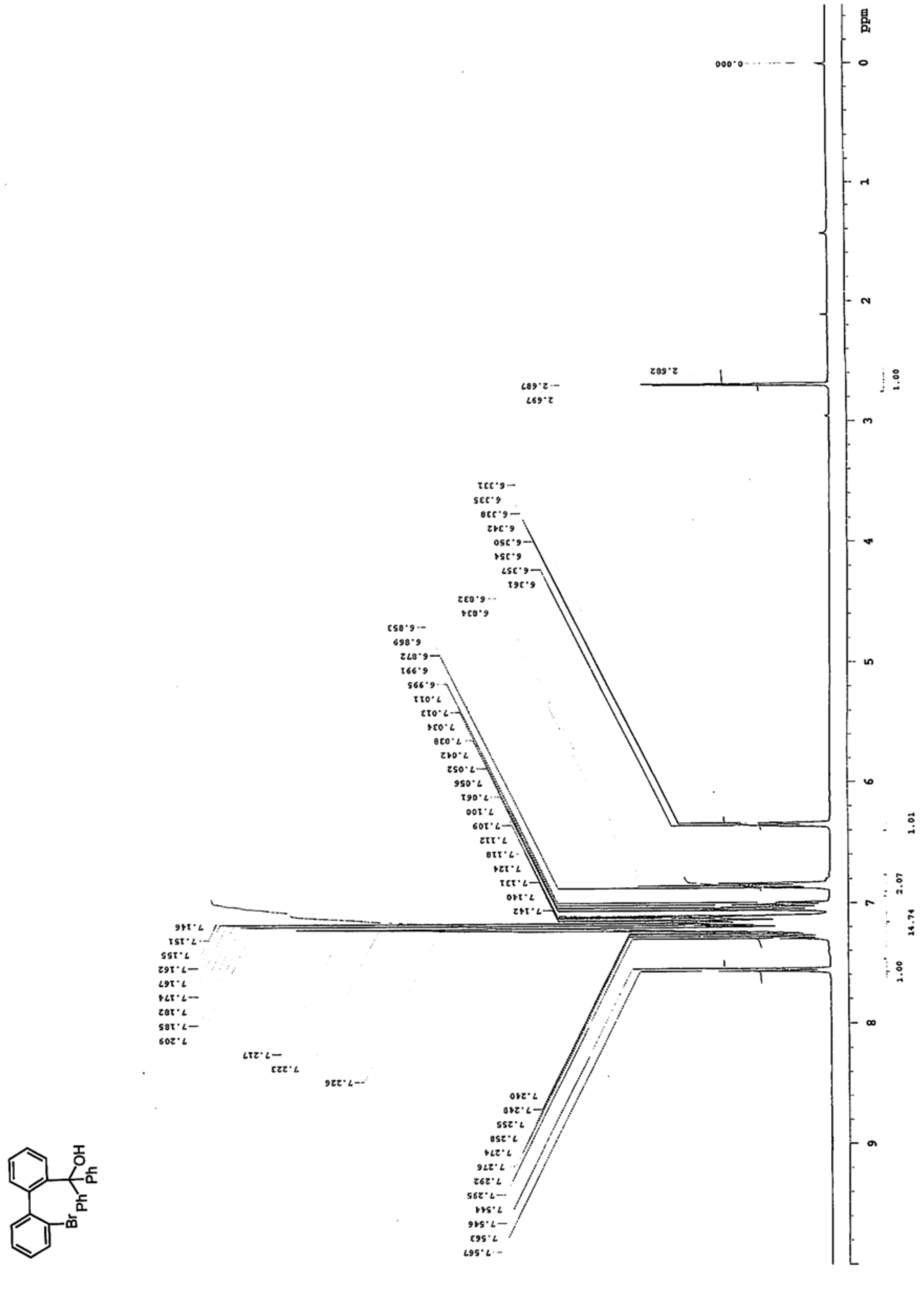

^{1}H NMR spectrum of 2-bromo-2’-[hydroxyl(diphenyl)methyl]biphenyl (**11**)

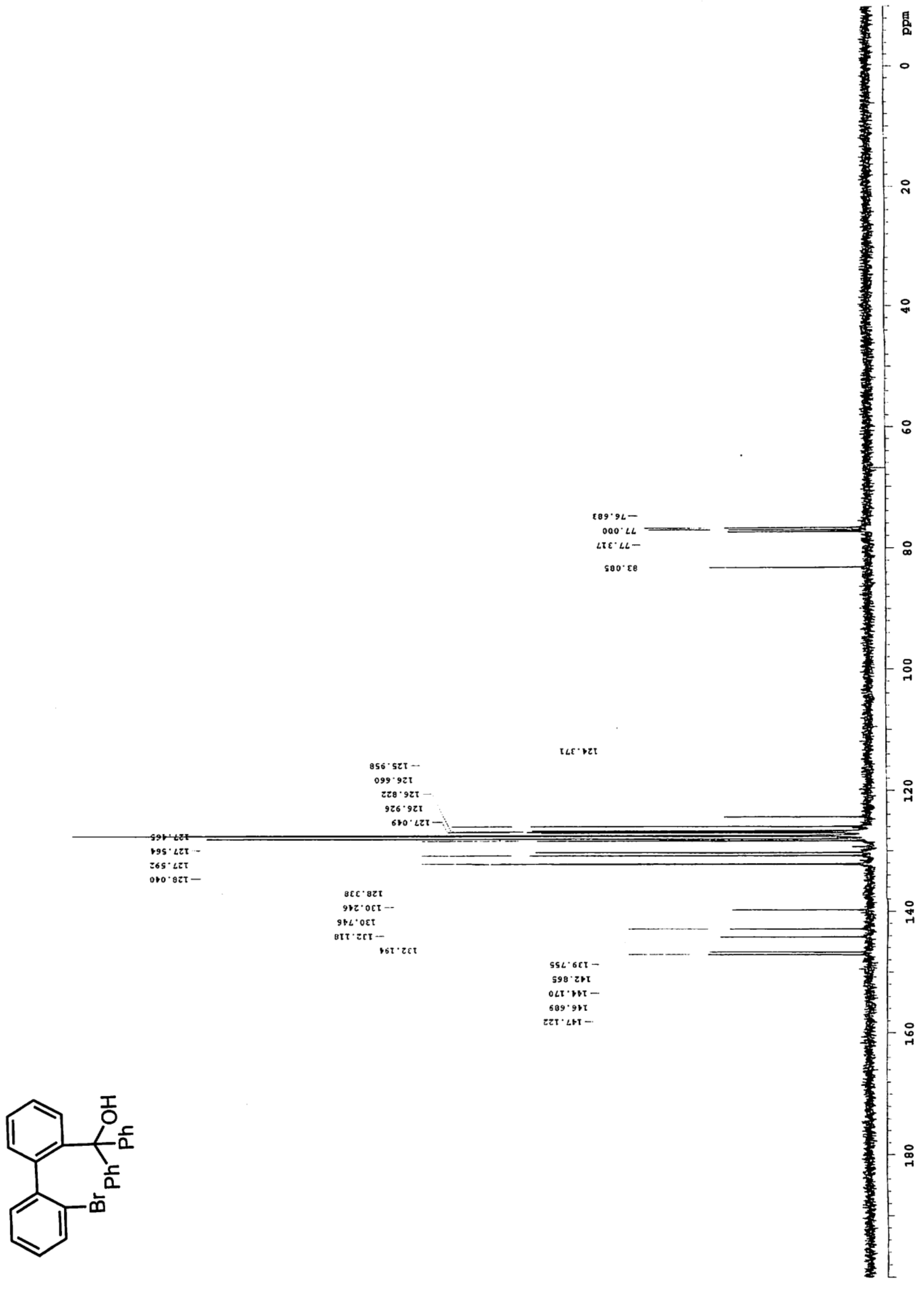

^{13}C NMR spectrum of 2-bromo-2’-[hydroxyl(diphenyl)methyl]biphenyl (11)

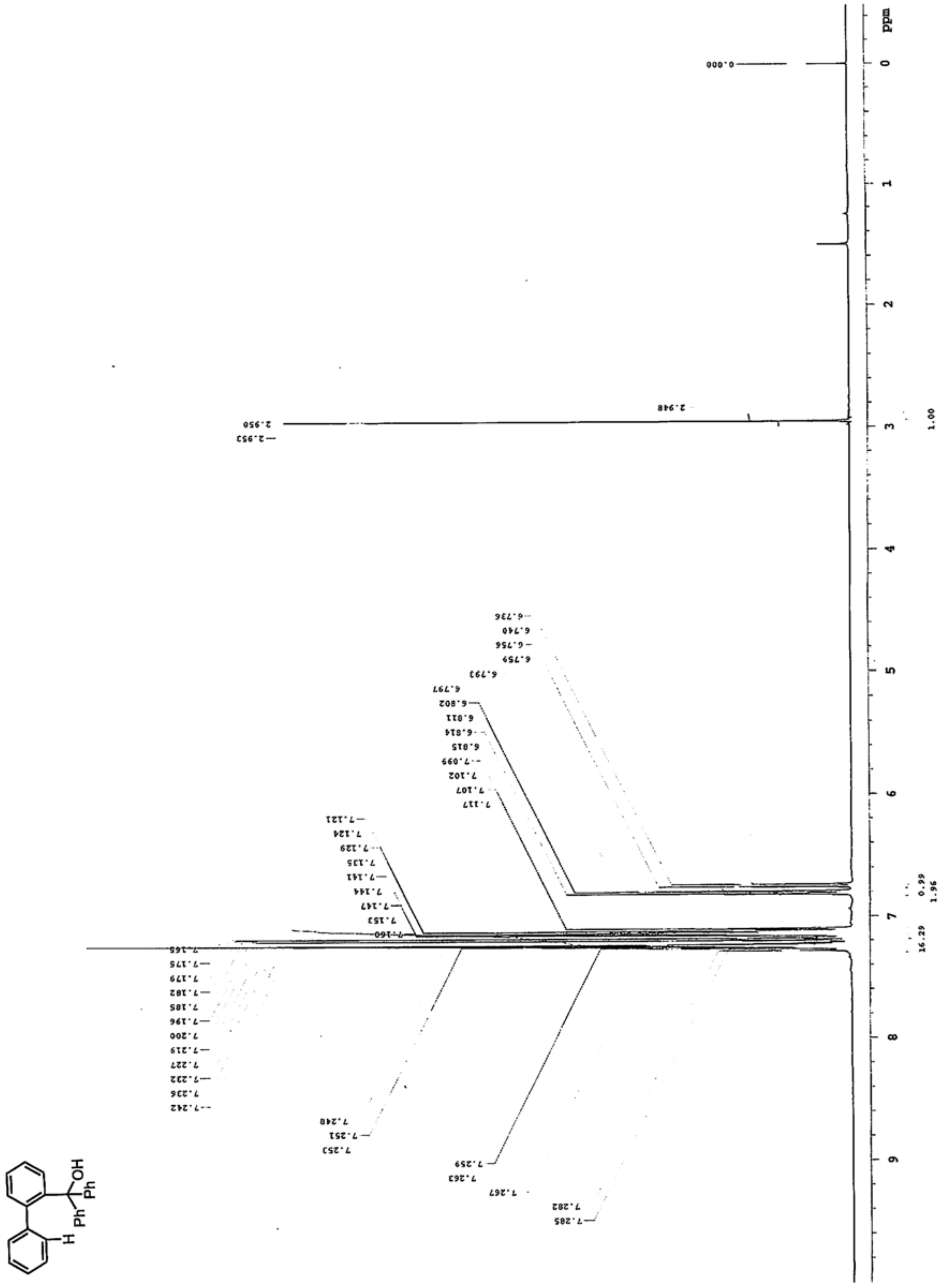

^{1}H NMR spectrum of 2-[hydroxyl(diphenyl)methyl]biphenyl (12)

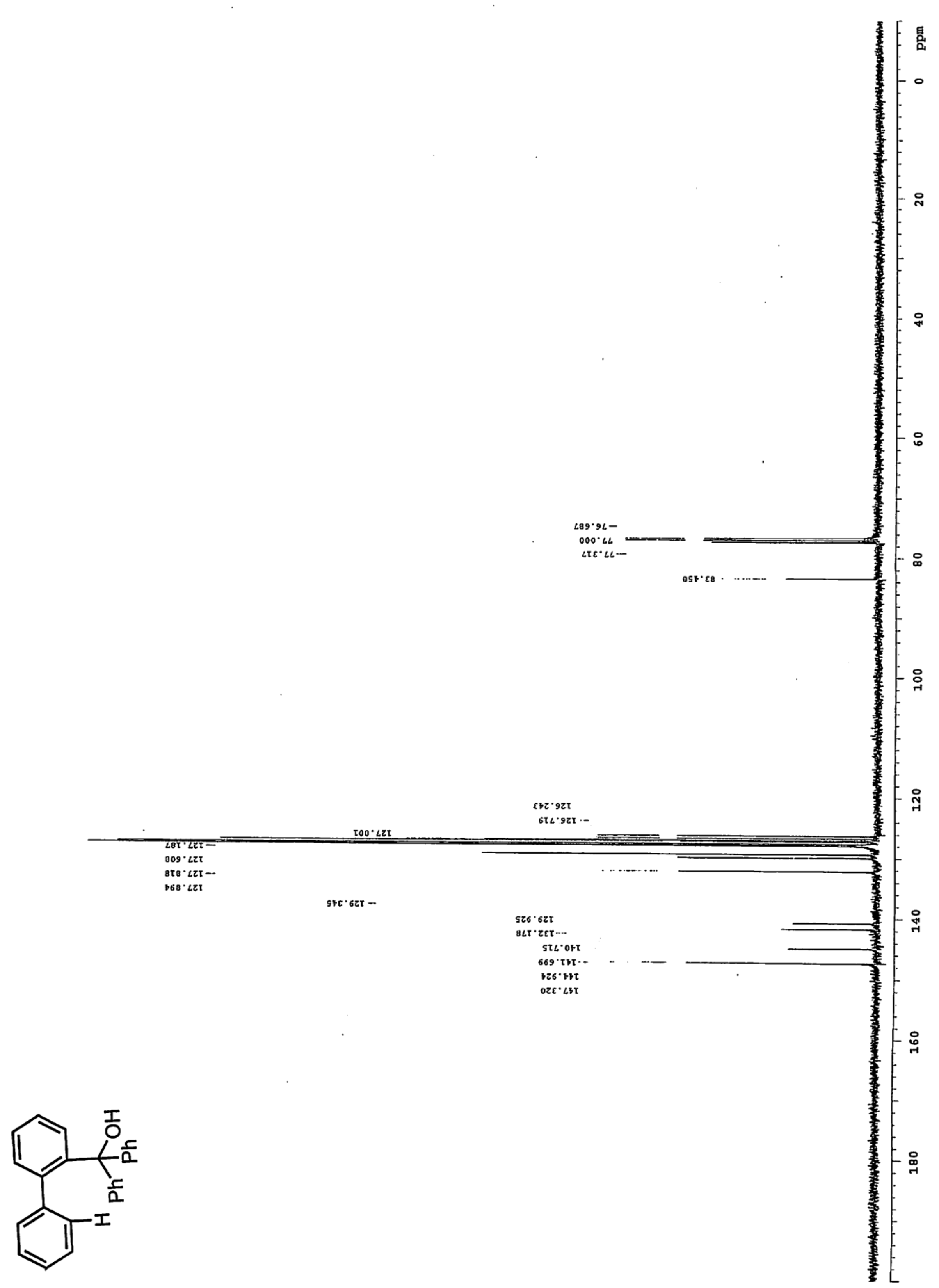

^{13}C NMR spectrum of 2-[hydroxyl(diphenyl)methyl]biphenyl (12)

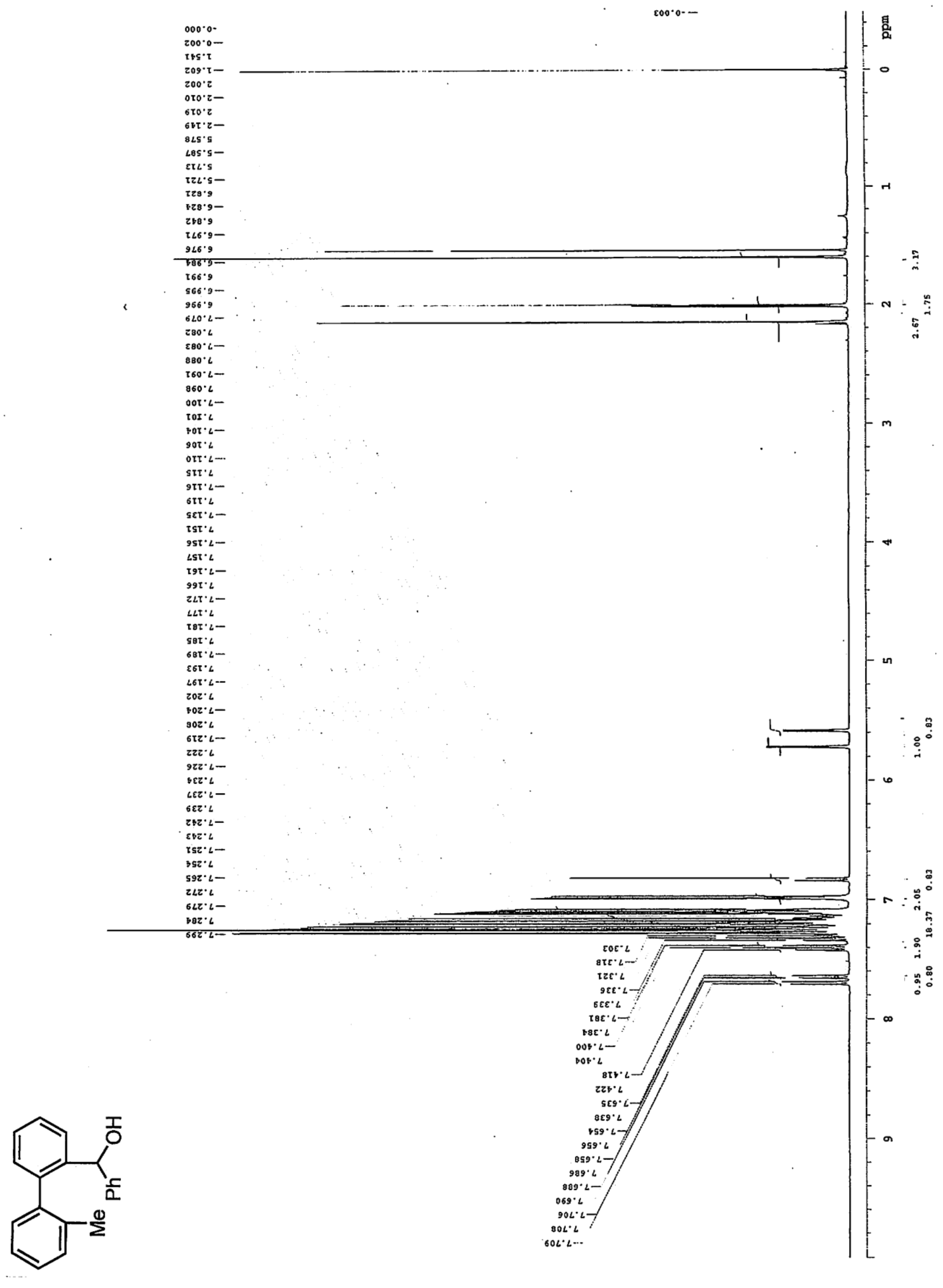

^{1}H NMR spectrum of 2-methyl-2'-[hydroxyl(phenyl)methyl]biphenyl (13)

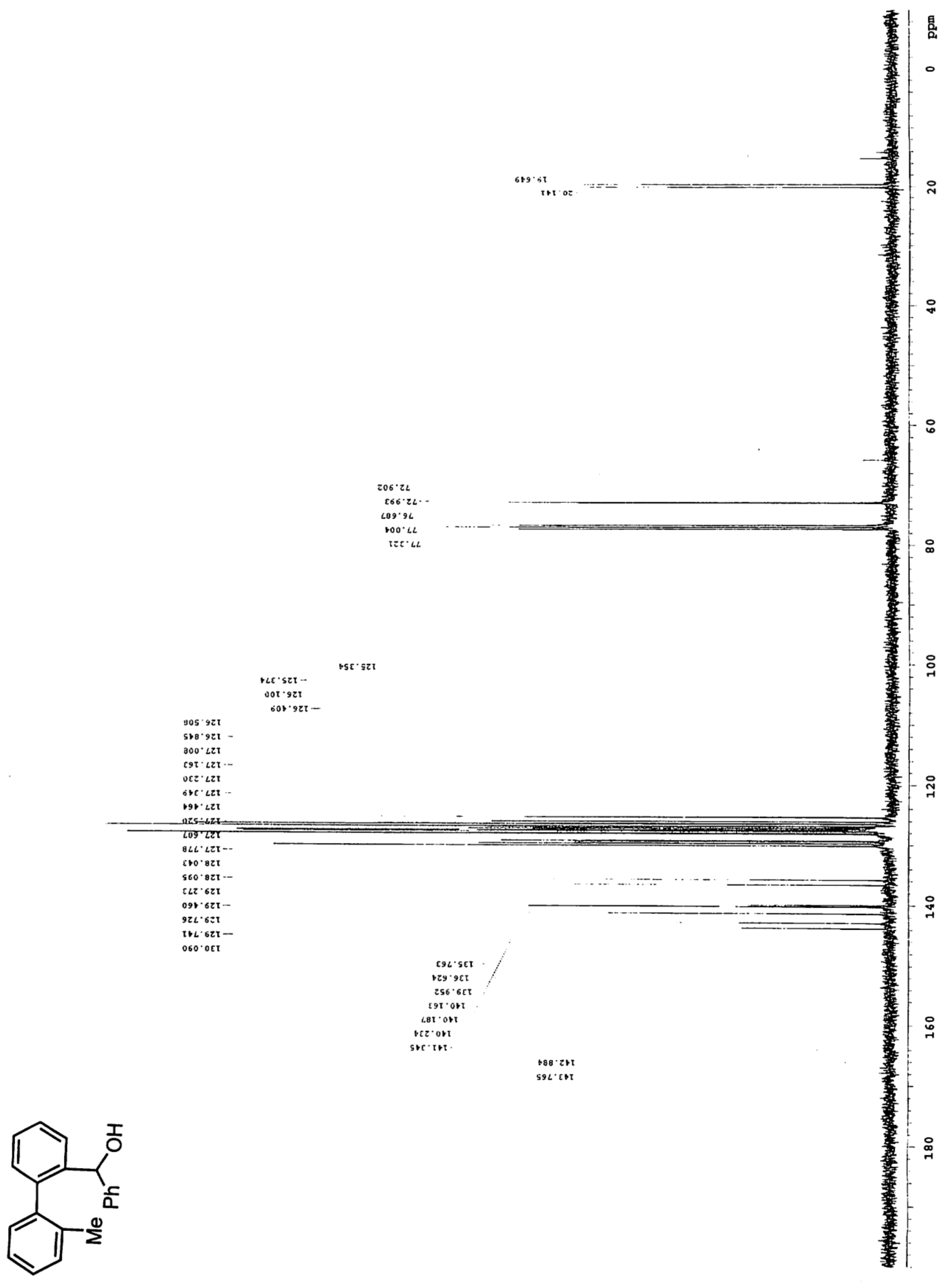

^{13}C NMR spectrum of 2-methyl-2’-[hydroxyl(phenyl)methyl]biphenyl (13)

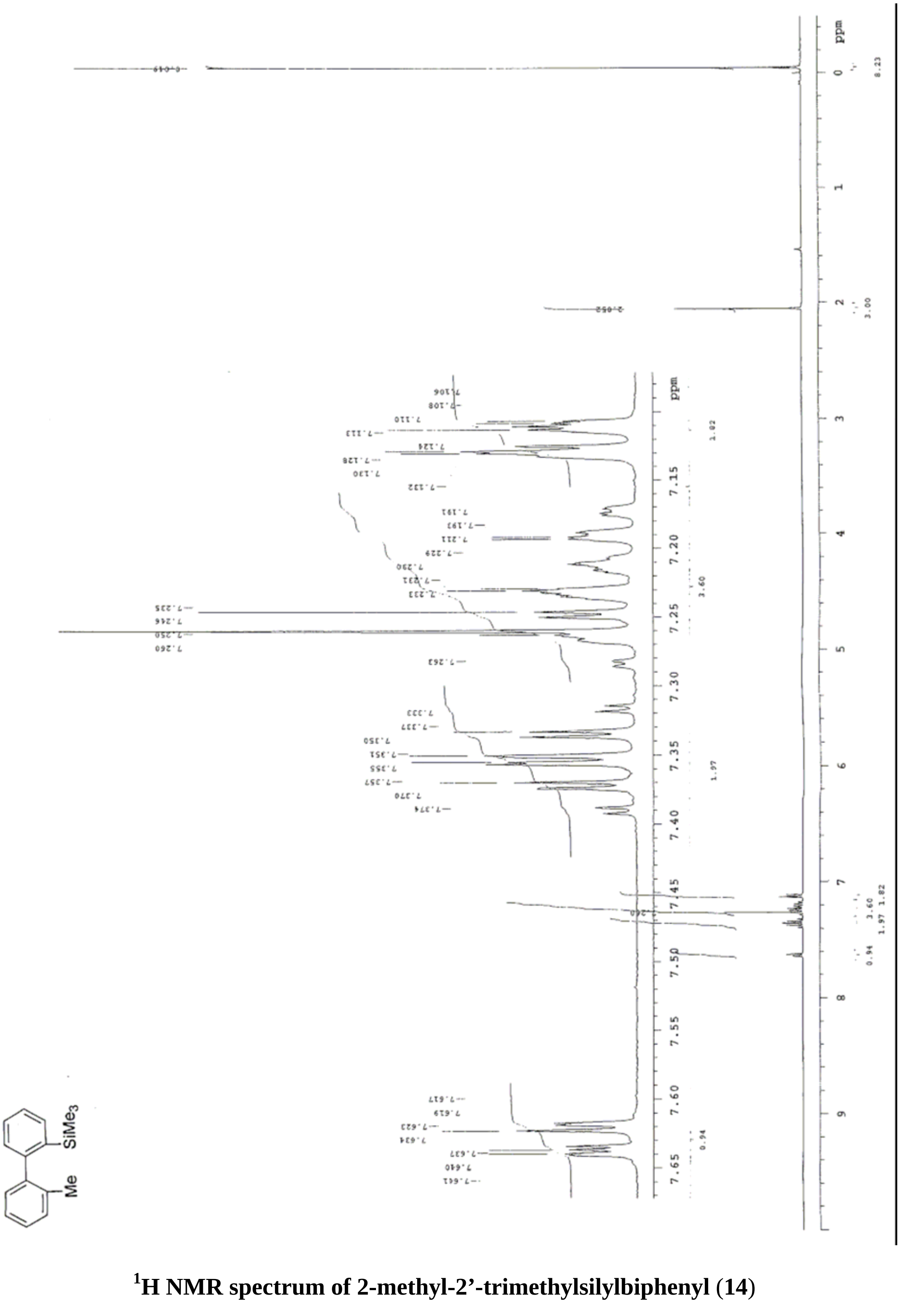

^{1}H NMR spectrum of 2-methyl-2'-trimethylsilylbiphenyl (14)

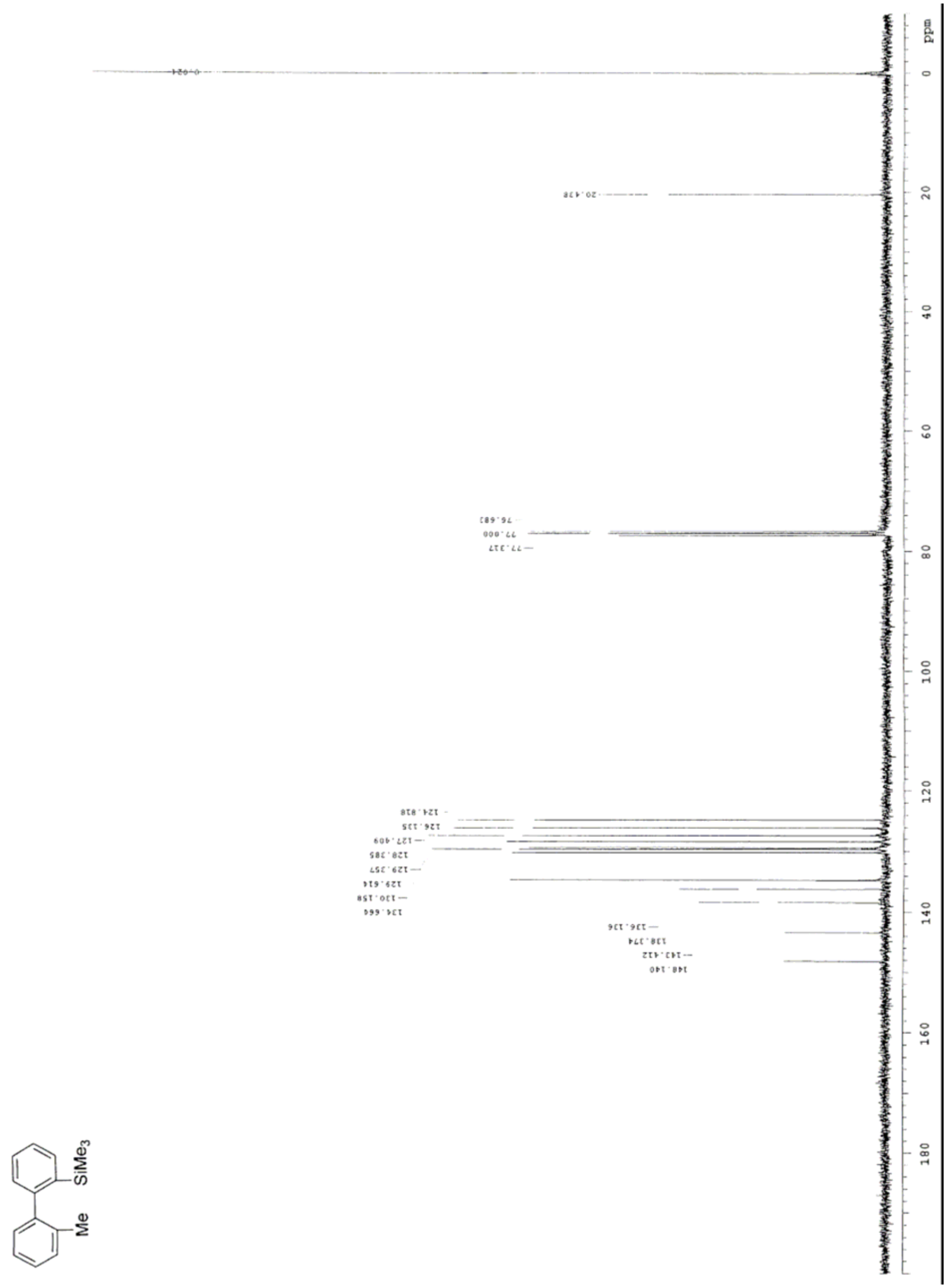

^{13}C NMR spectrum of 2-methyl-2'-trimethylsilylbiphenyl (14)

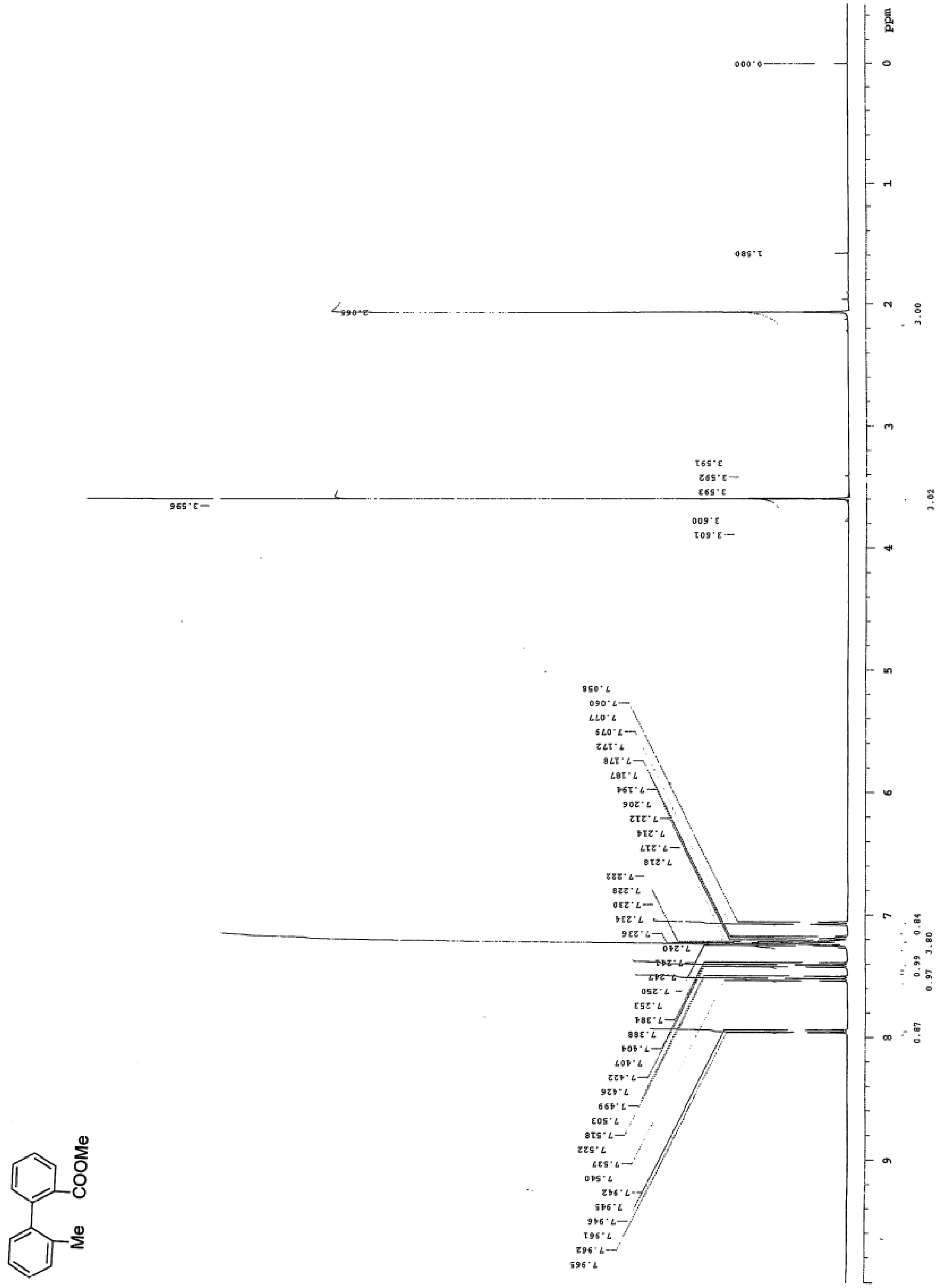

^{1}H NMR spectrum of 2-methyl-2'-methoxycarbonylbiphenyl (15)

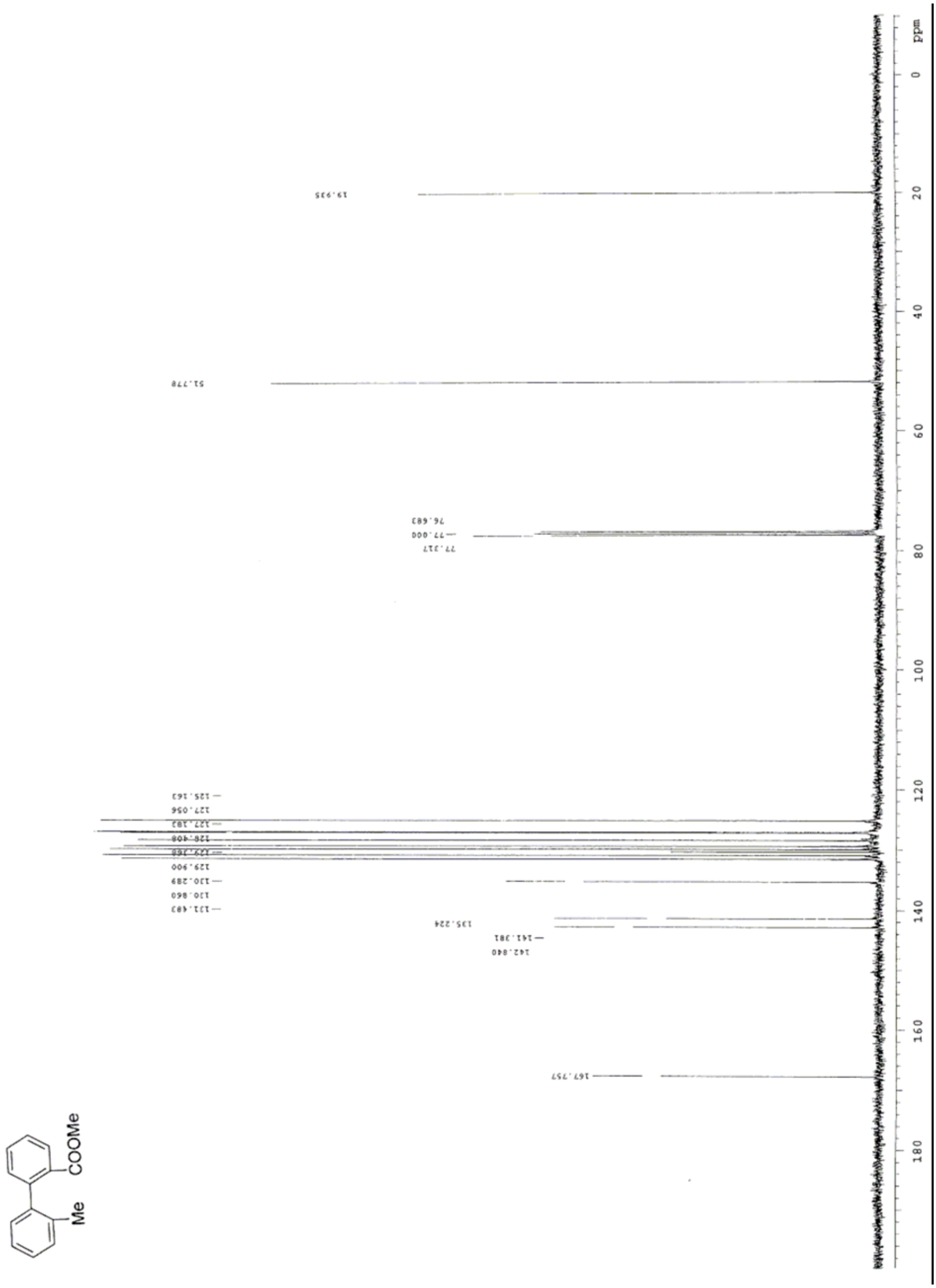

^{13}C NMR spectrum of 2-methyl-2’-methoxycarbonylbiphenyl (15)

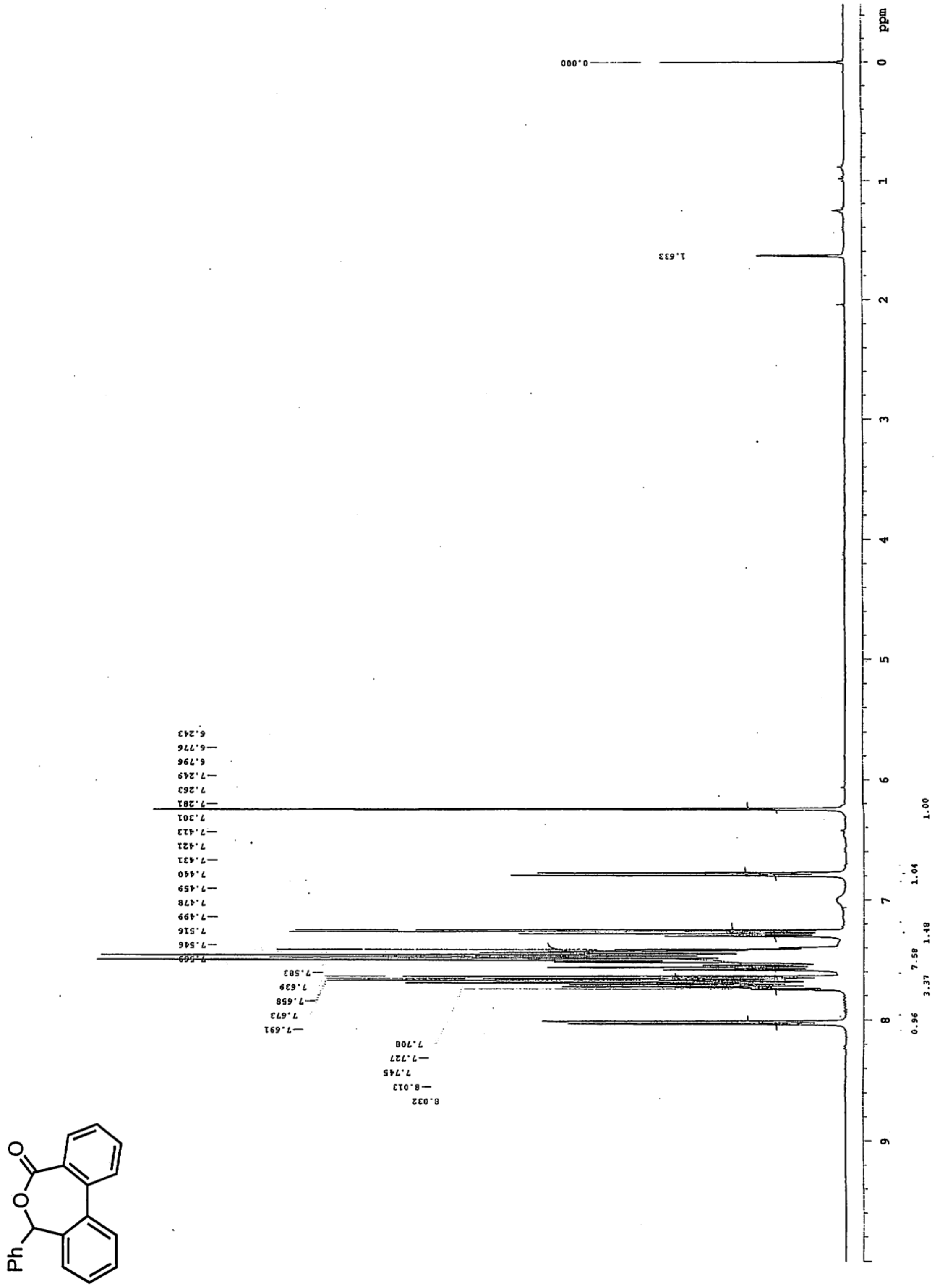

^{1}H NMR spectrum of 7-phenyl-5,7-dihydrodibenz[c,e]oxepin-5-one (16)

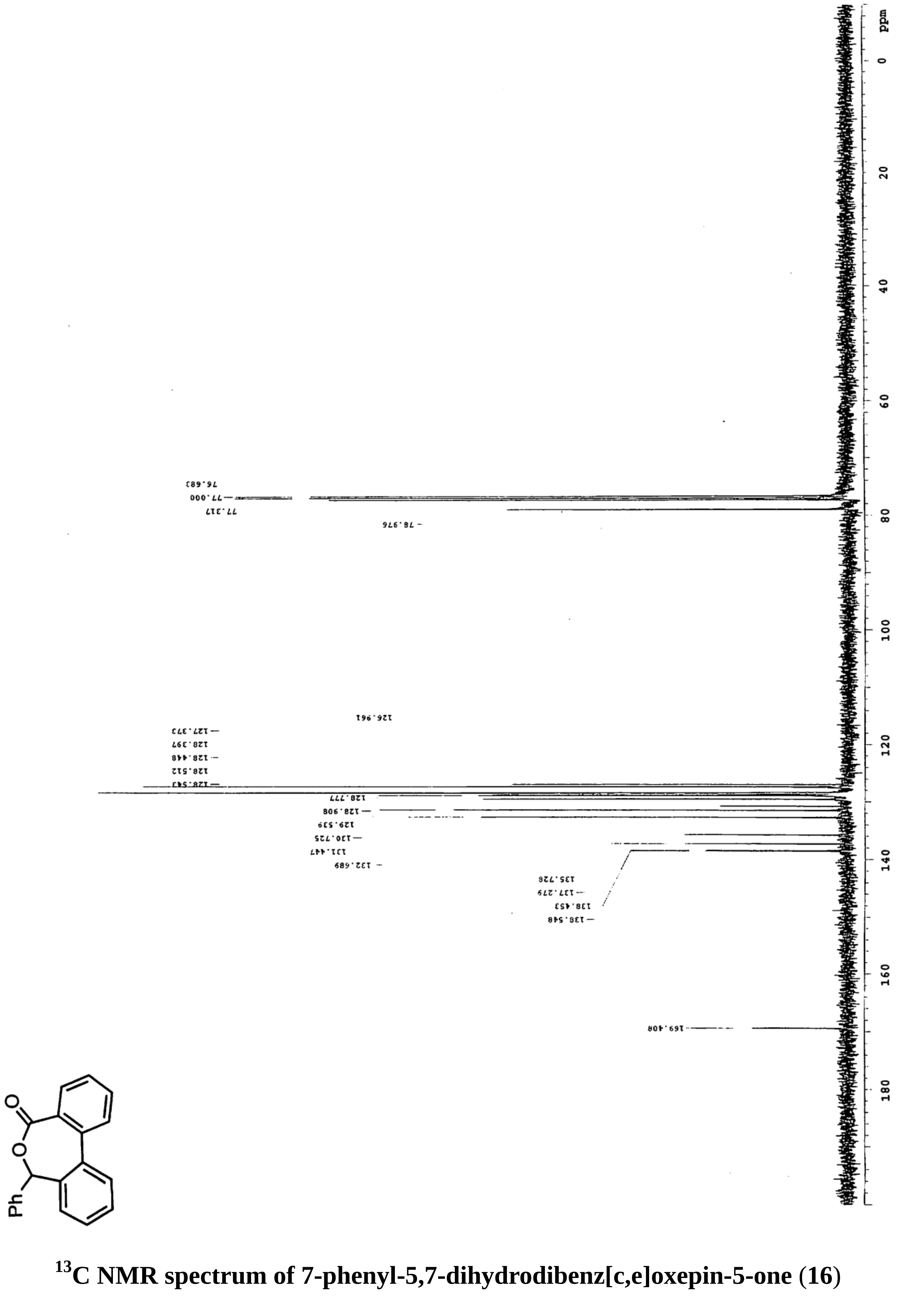

^{13}C NMR spectrum of 7-phenyl-5,7-dihydrodibenz[c,e]oxepin-5-one (16)

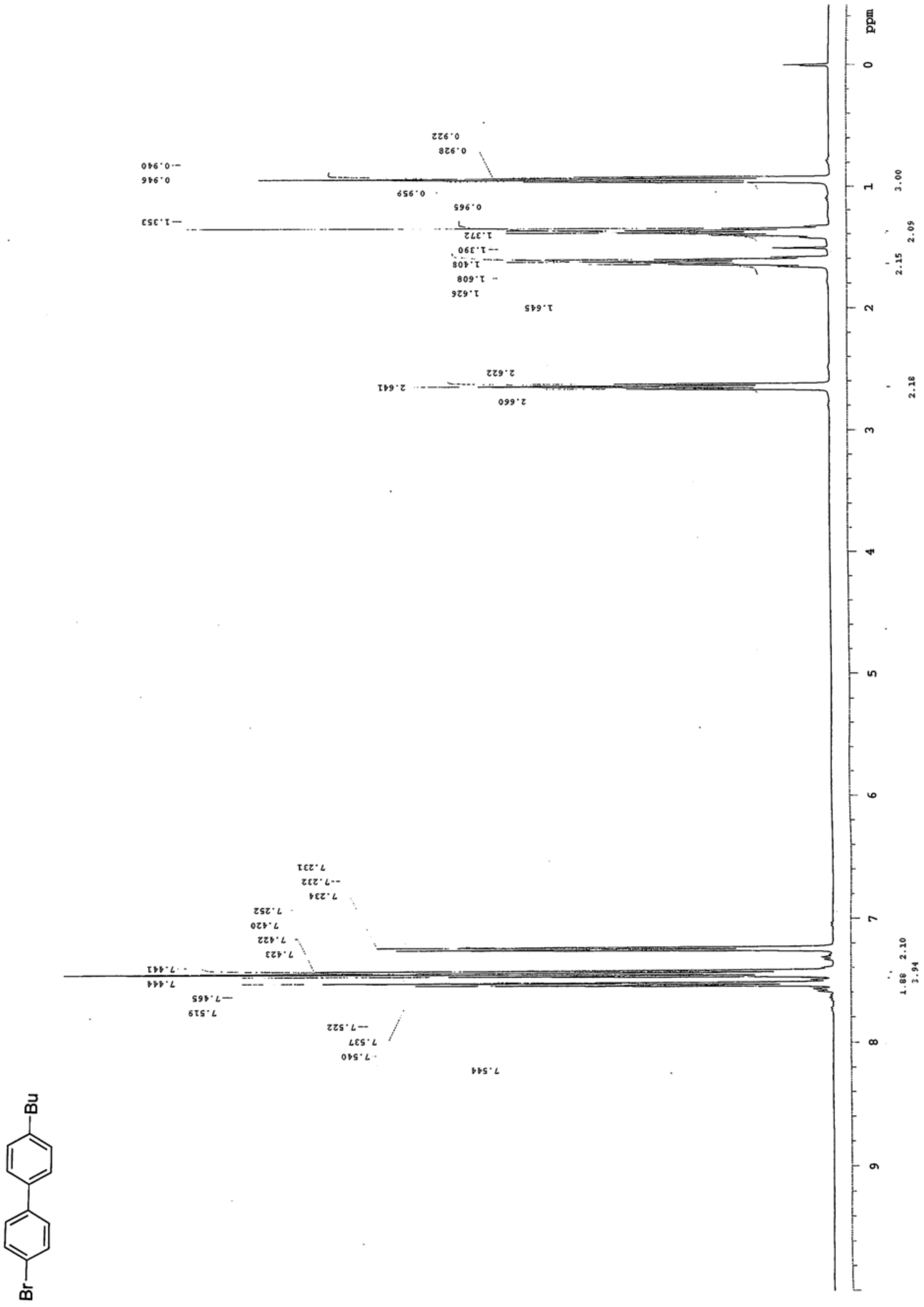

^{1}H NMR spectrum of 4-bromo-4'-butylbiphenyl (19)

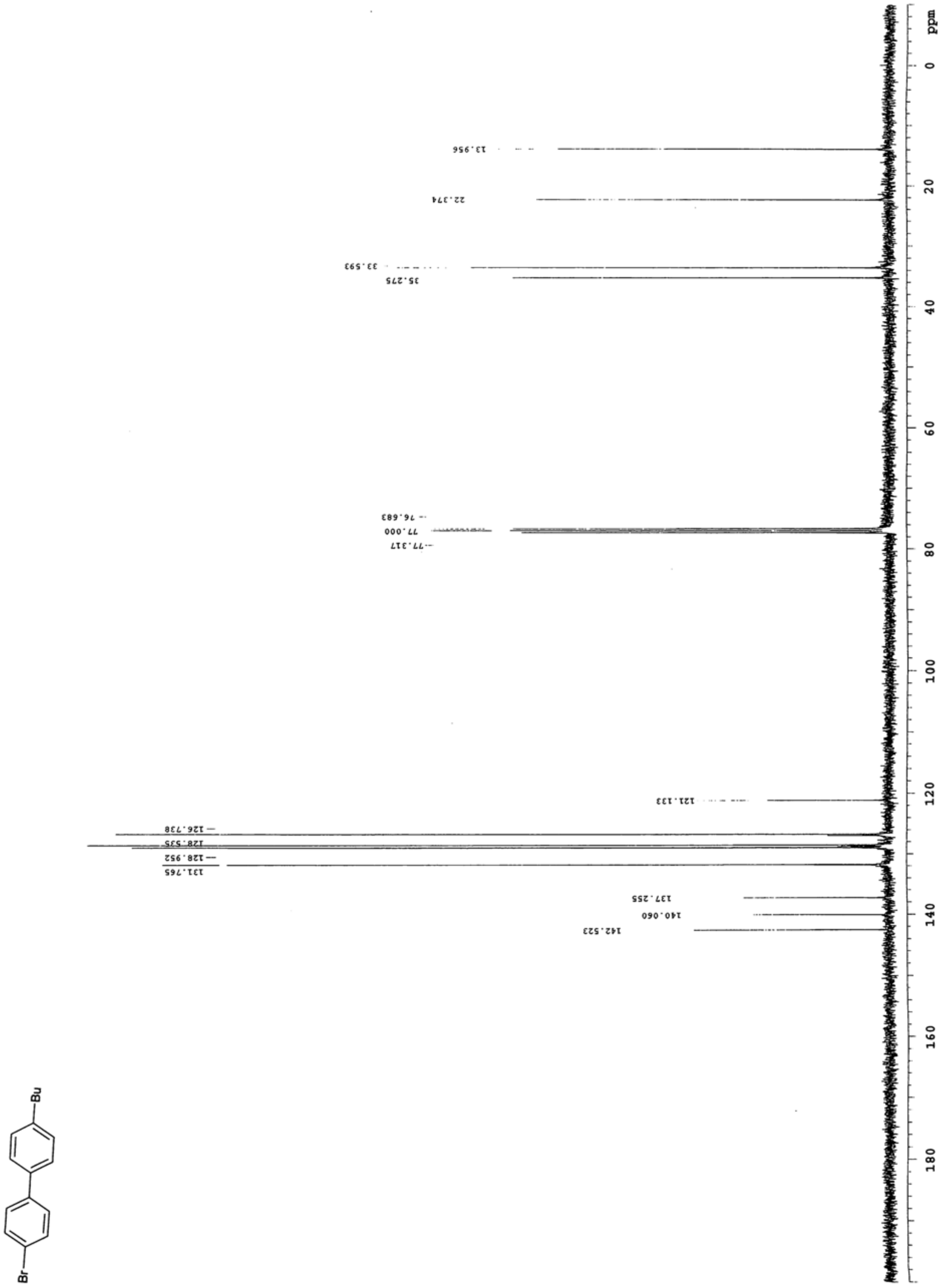

^{13}C NMR spectrum of 4-bromo-4'-butylbiphenyl (19)

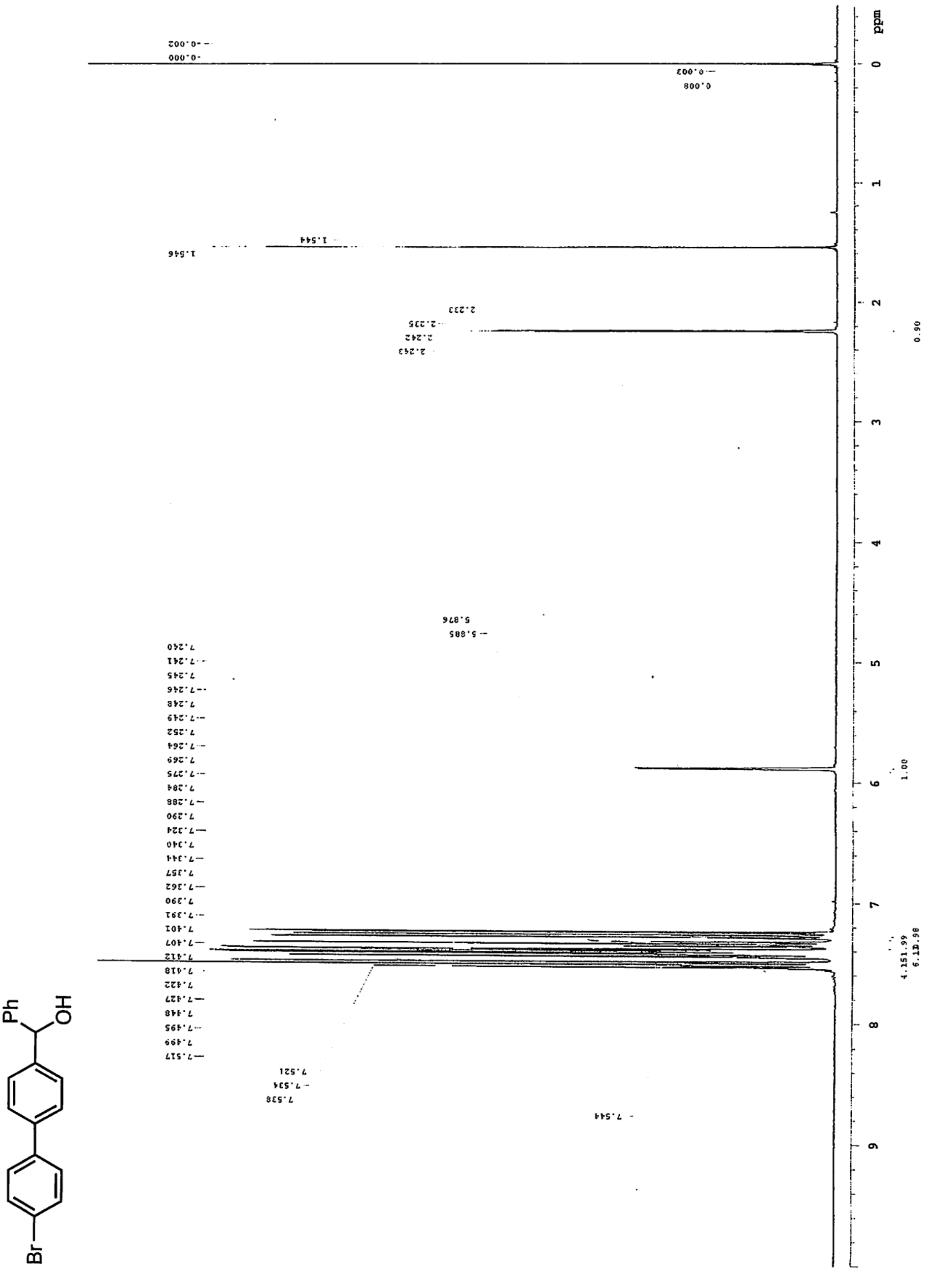

^{1}H NMR spectrum of 4-bromo-4’-[hydroxyl(phenyl)methyl]biphenyl (22)

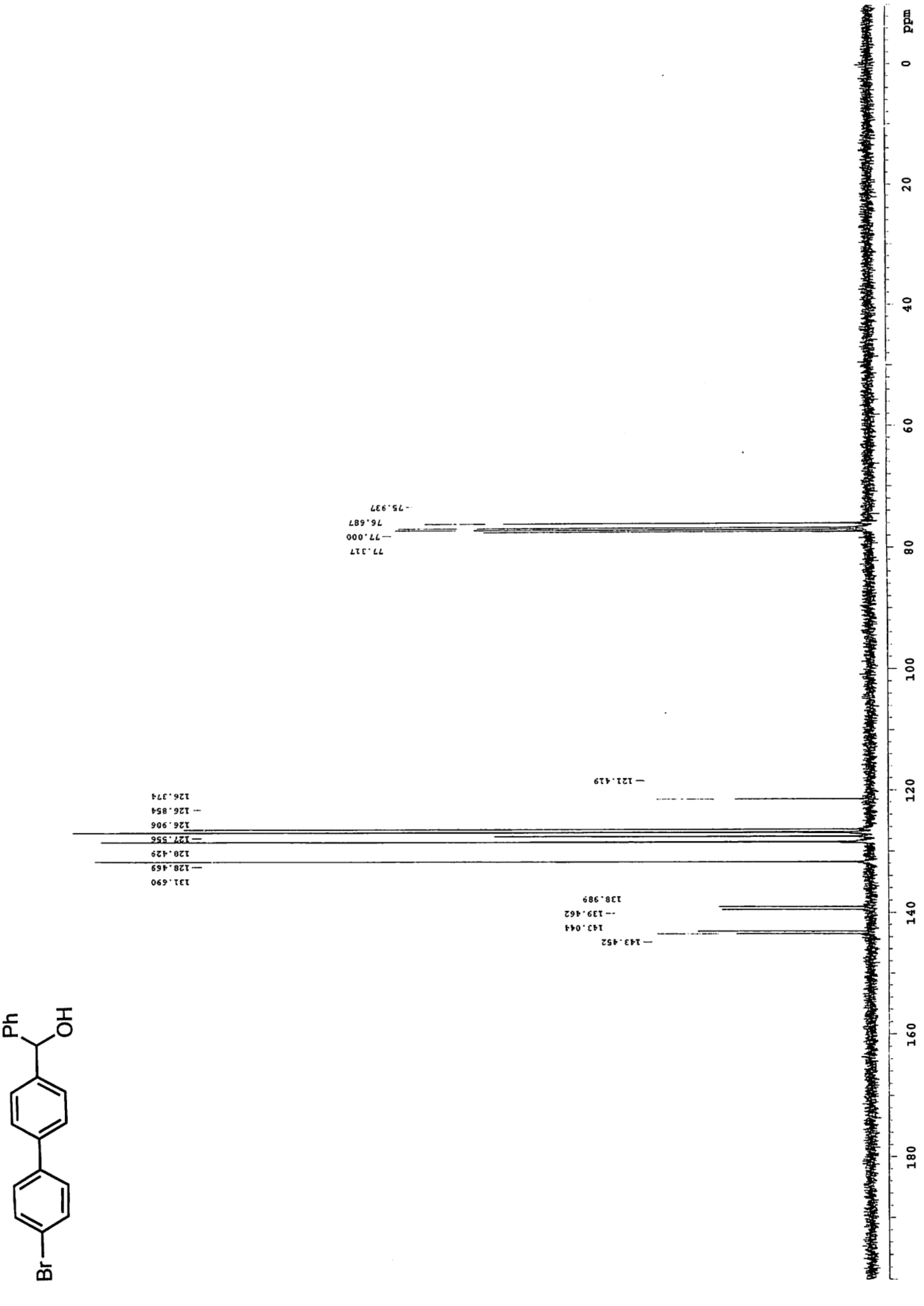

^{13}C NMR spectrum of 4-bromo-4'-[hydroxyl(phenyl)methyl]biphenyl (**22**)

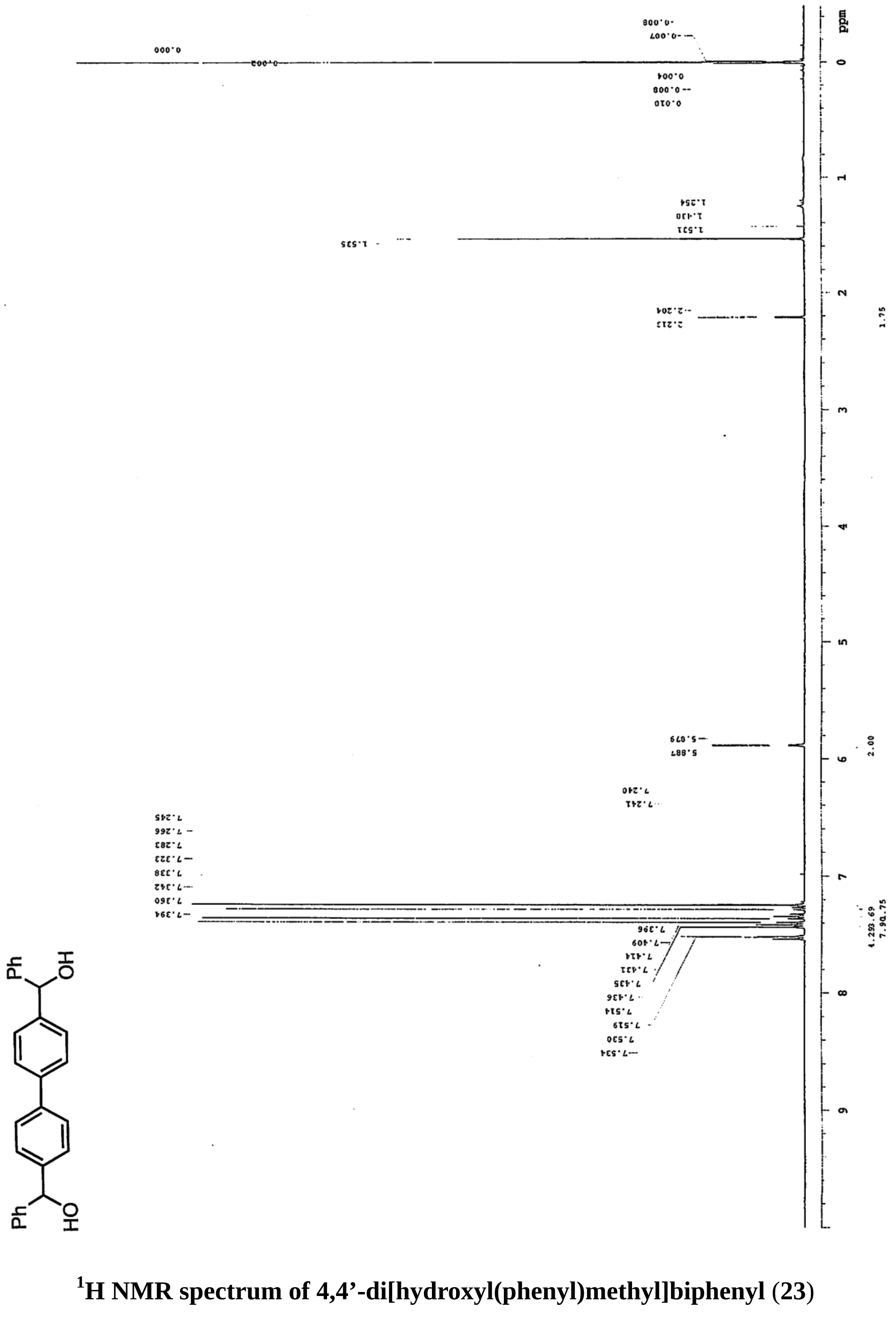

^{1}H NMR spectrum of 4,4'-di[hydroxyl(phenyl)methyl]biphenyl (23)

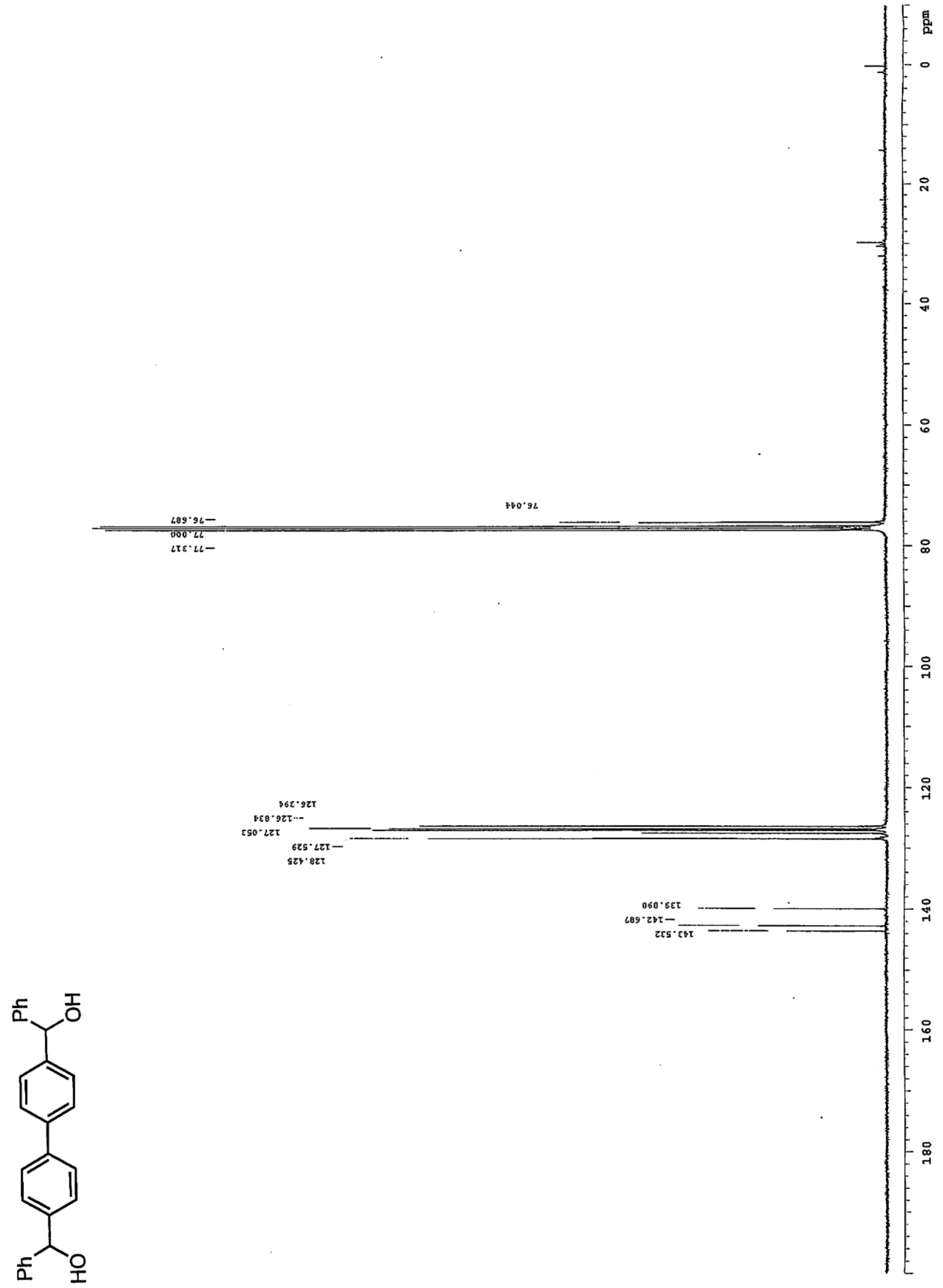

^{13}C NMR spectrum of 4,4'-di[hydroxyl(phenyl)methyl]biphenyl (23)

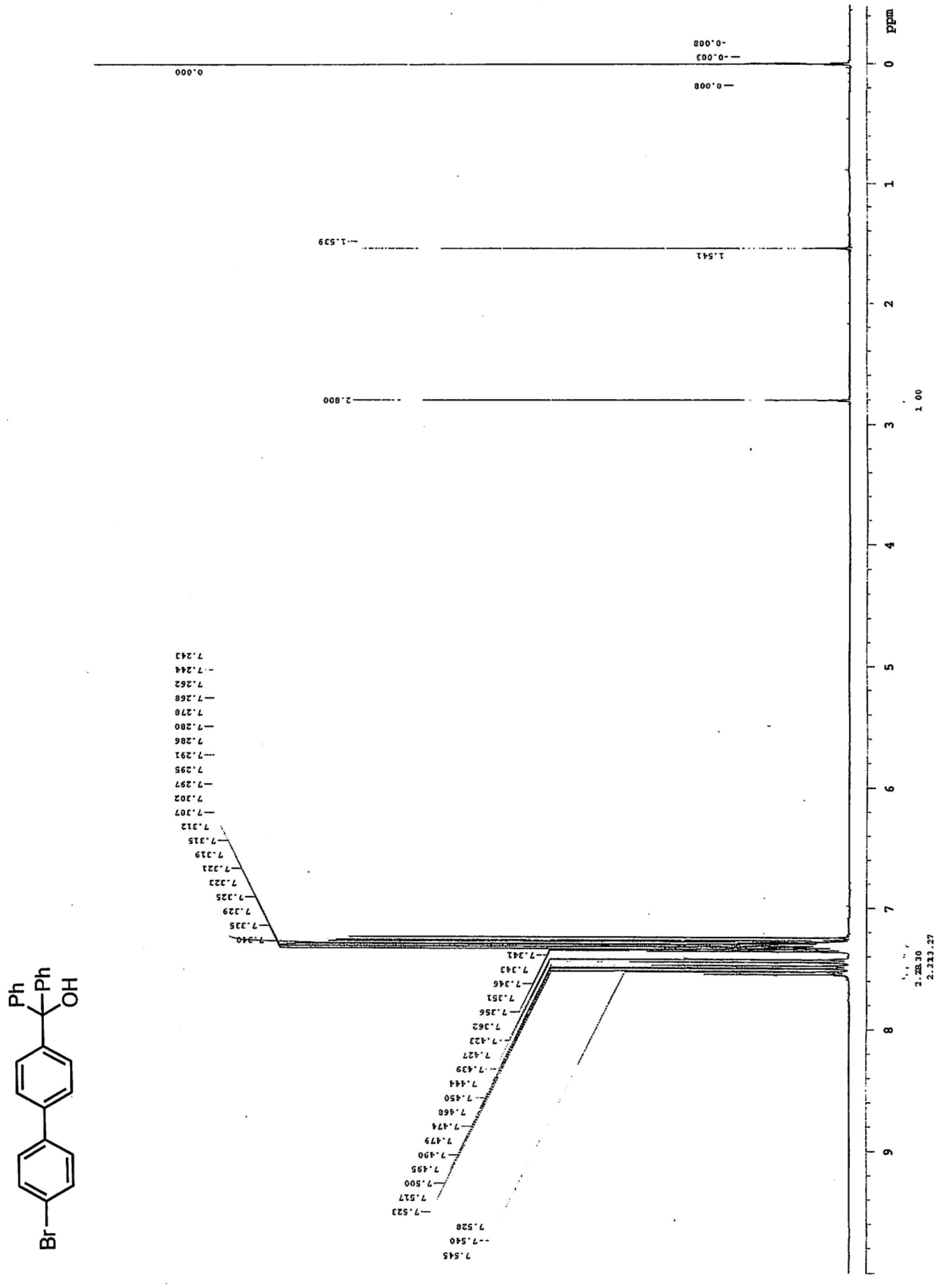

^{1}H NMR spectrum of 4-bromo-4’-[hydroxyl(diphenyl)methyl]biphenyl (**24**)

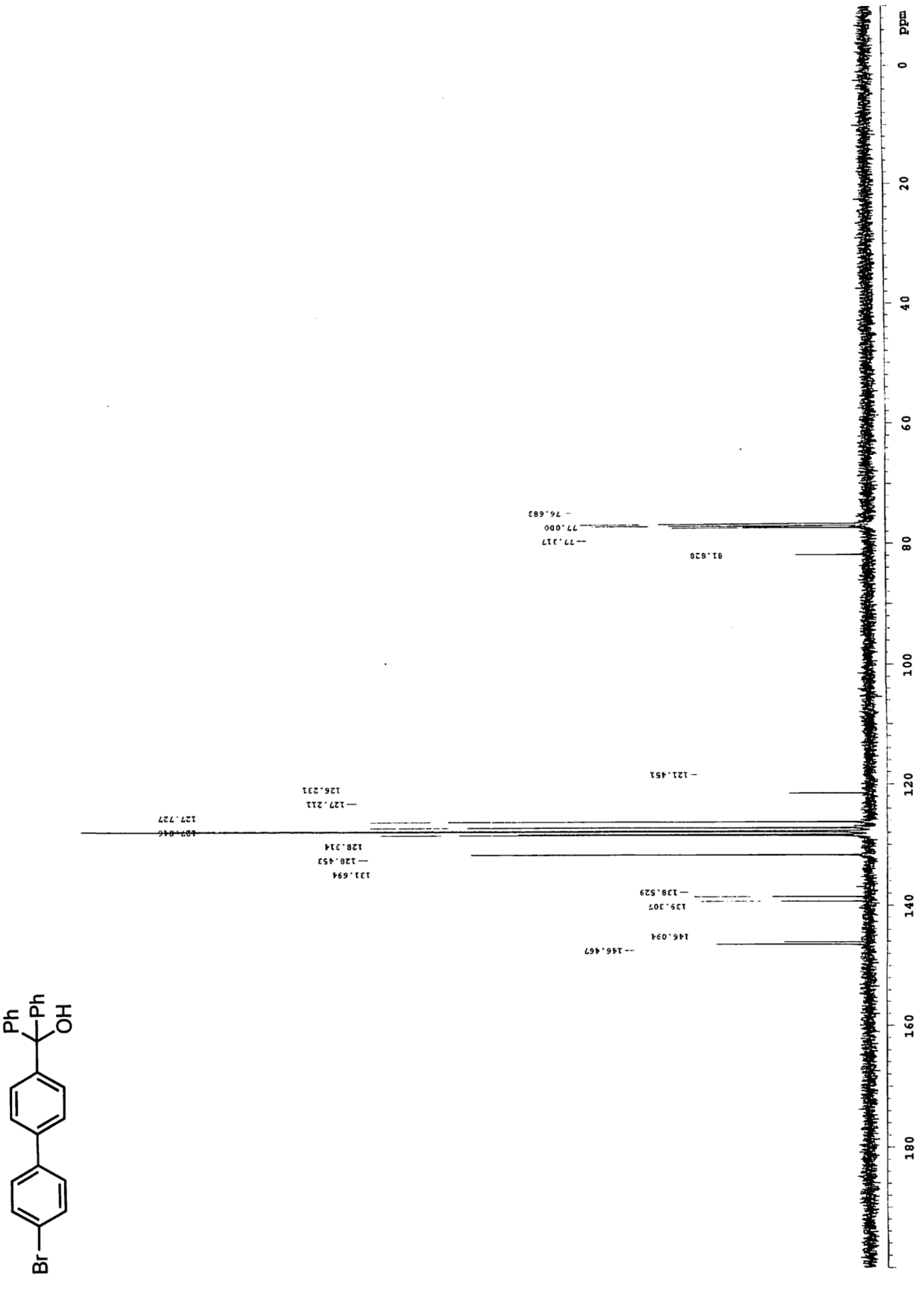

^{13}C NMR spectrum of 4-bromo-4'-[hydroxyl(diphenyl)methyl]biphenyl (24)

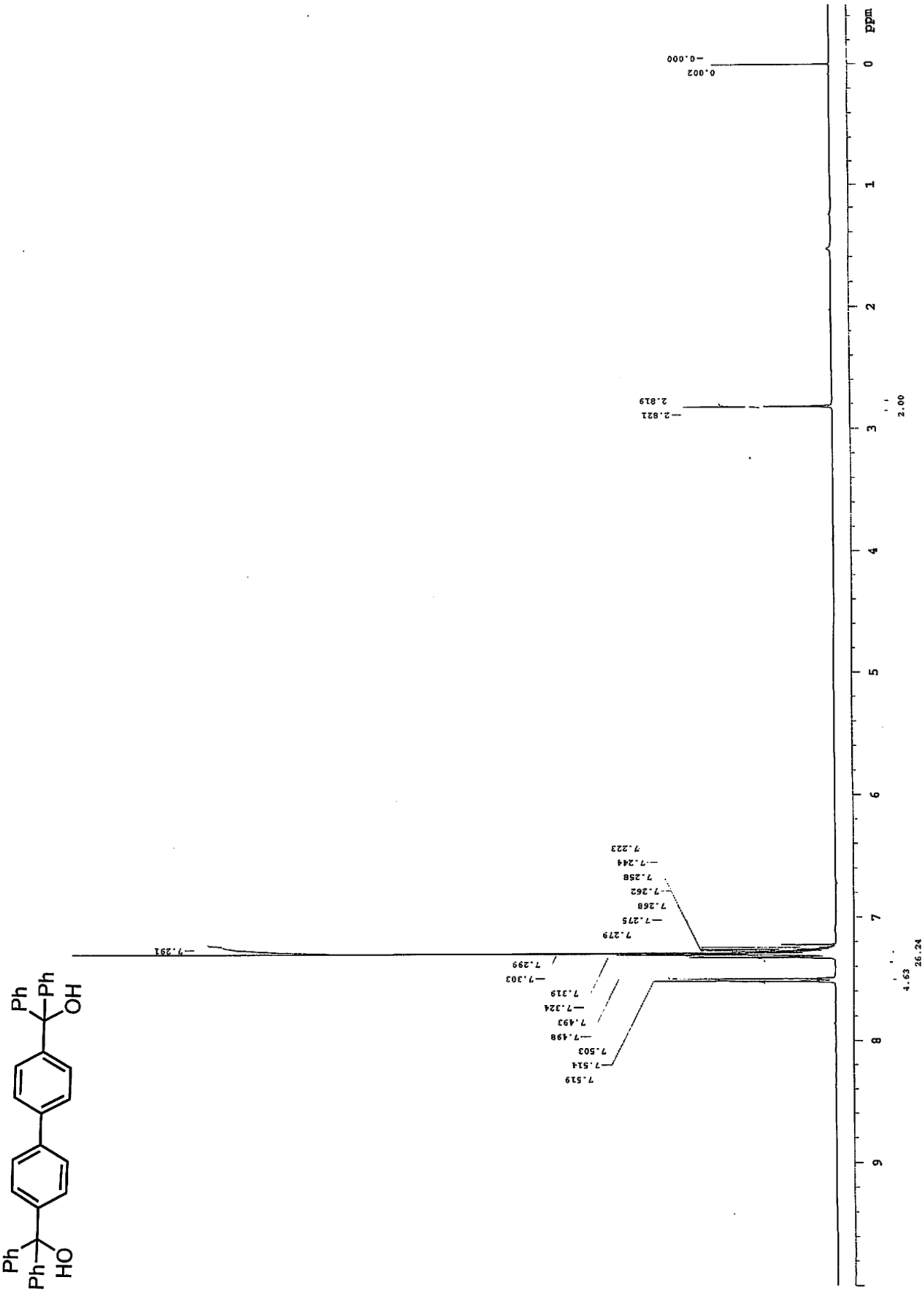

^{1}H NMR spectrum of 4,4'-bis[hydroxyl(diphenyl)methyl]biphenyl (25)

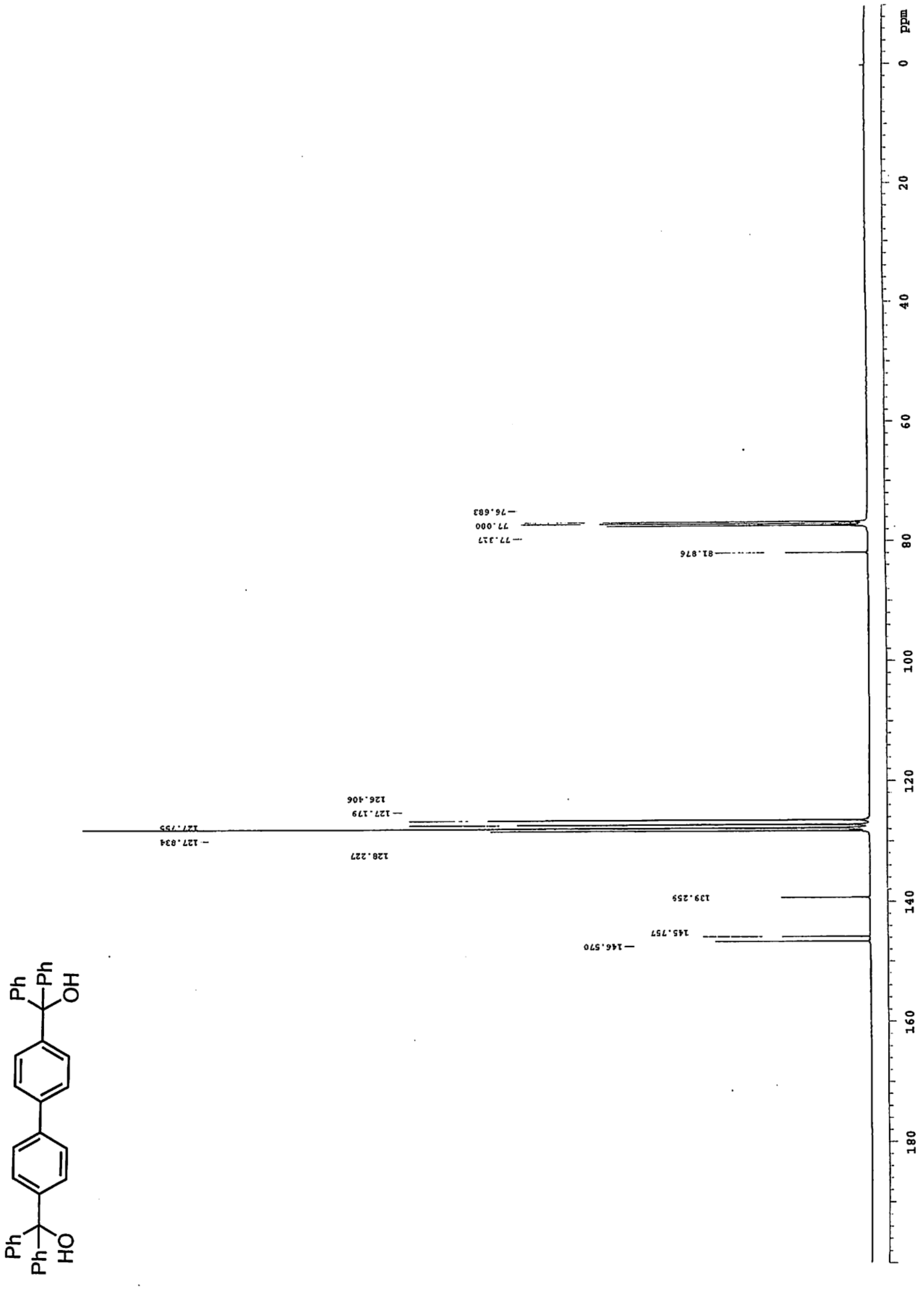

^{13}C NMR spectrum of 4,4'-bis[hydroxyl(diphenyl)methyl]biphenyl (**25**)

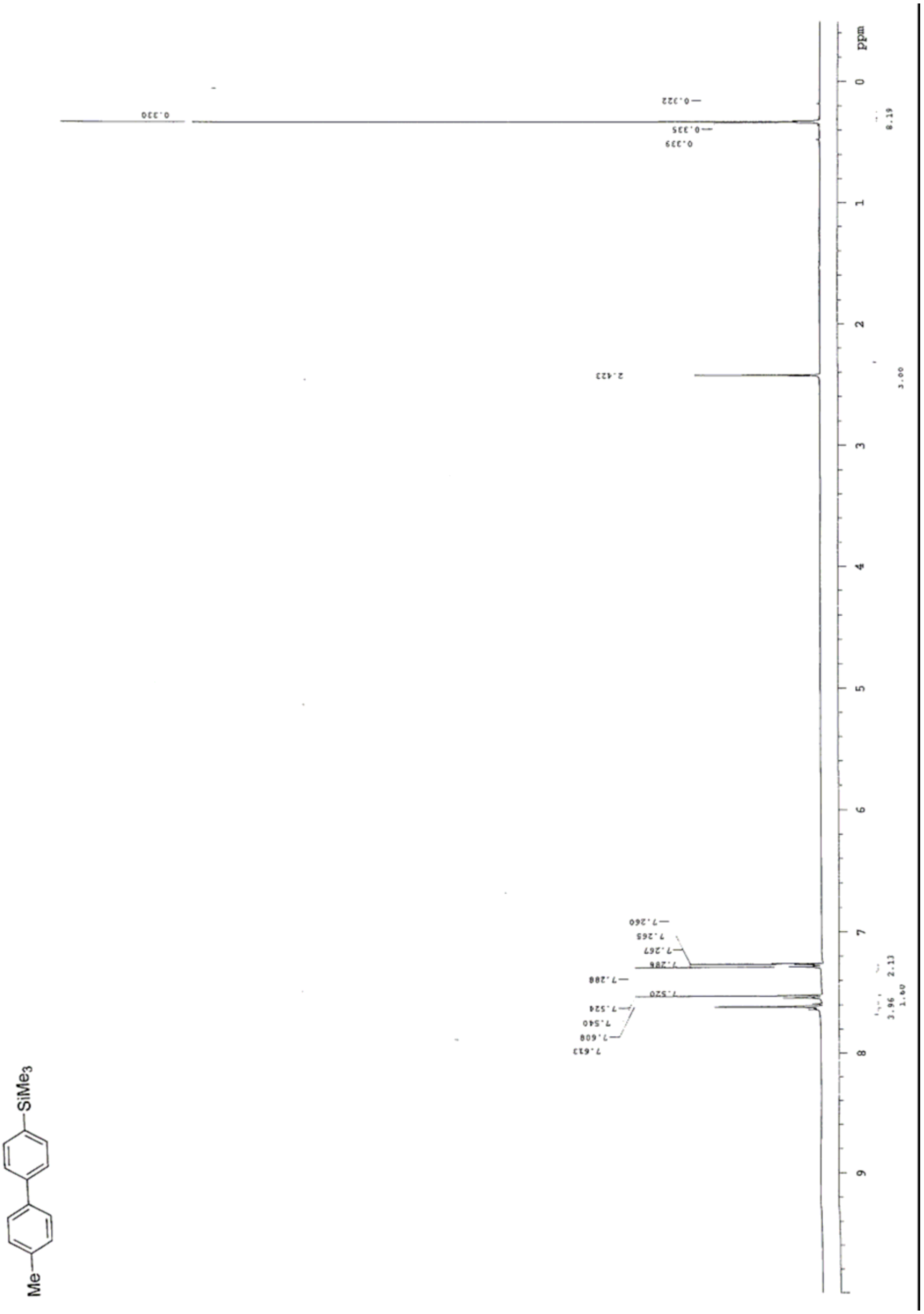

^{1}H NMR spectrum of 4-methyl-4’-trimethylsilylbiphenyl (26)

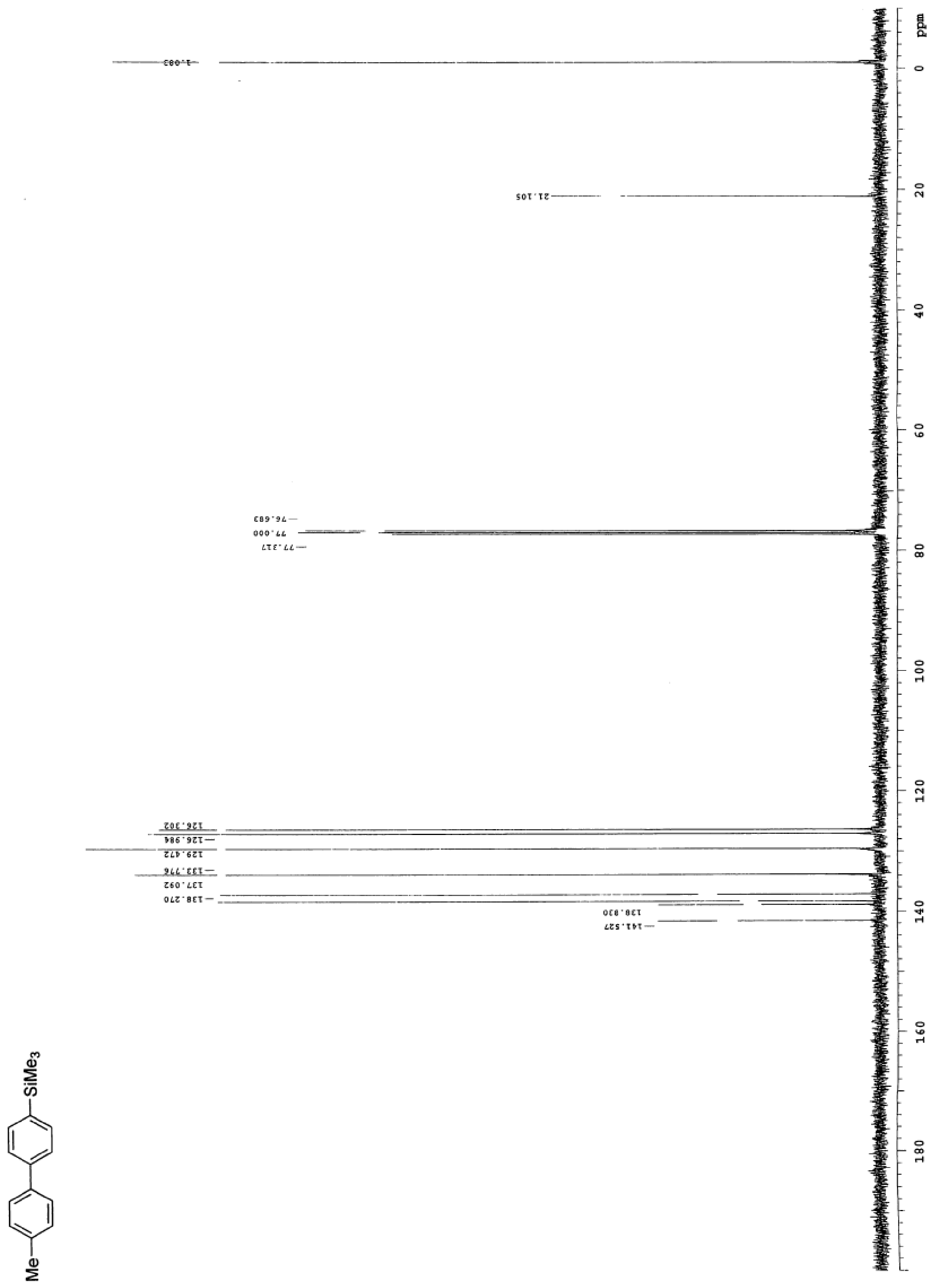

^{13}C NMR spectrum of 4-methyl-4’-trimethylsilylbiphenyl (**26**)

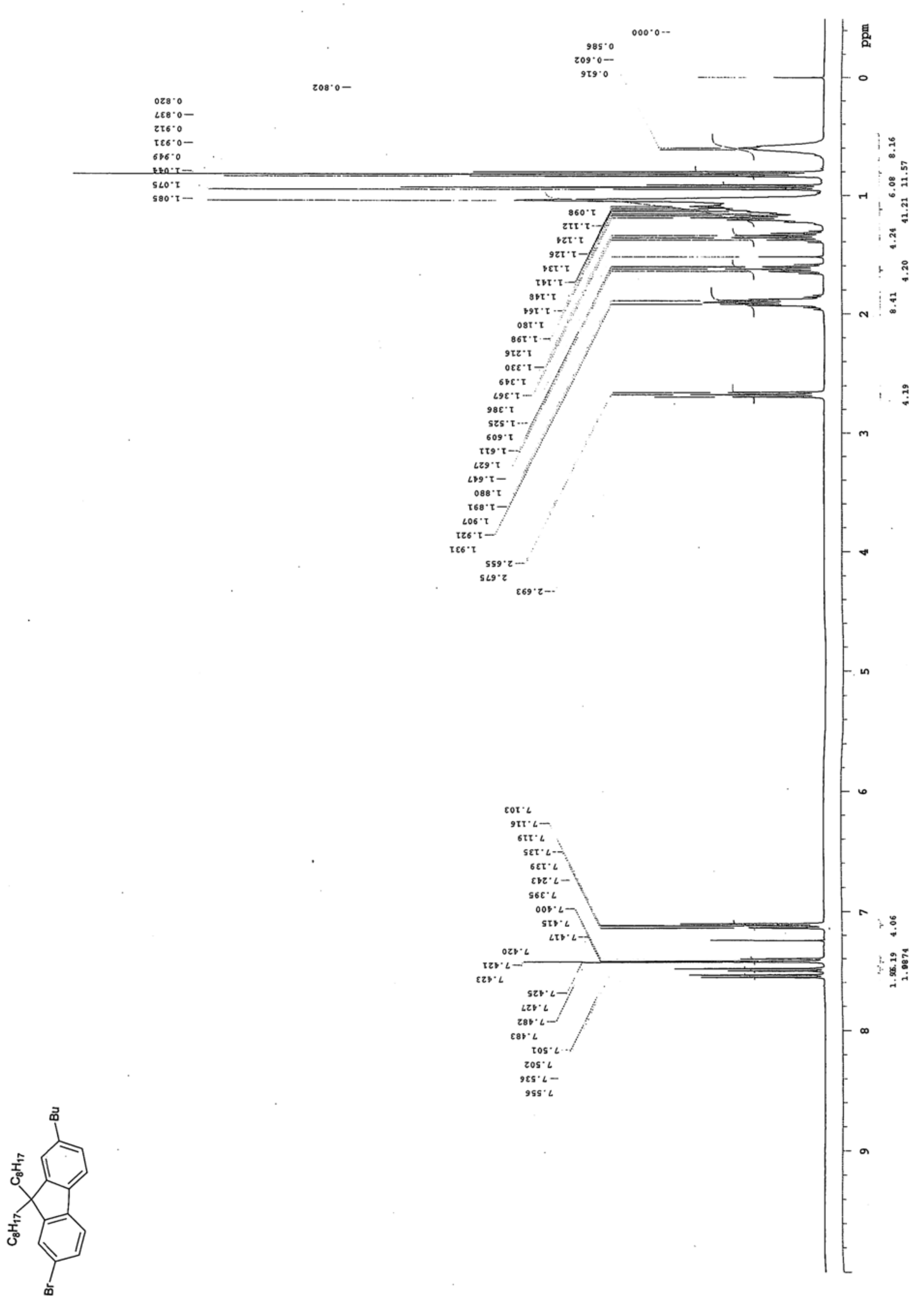

^{1}H NMR spectrum of 2-bromo-7-butyl-9,9-dioctylfluorene (31)

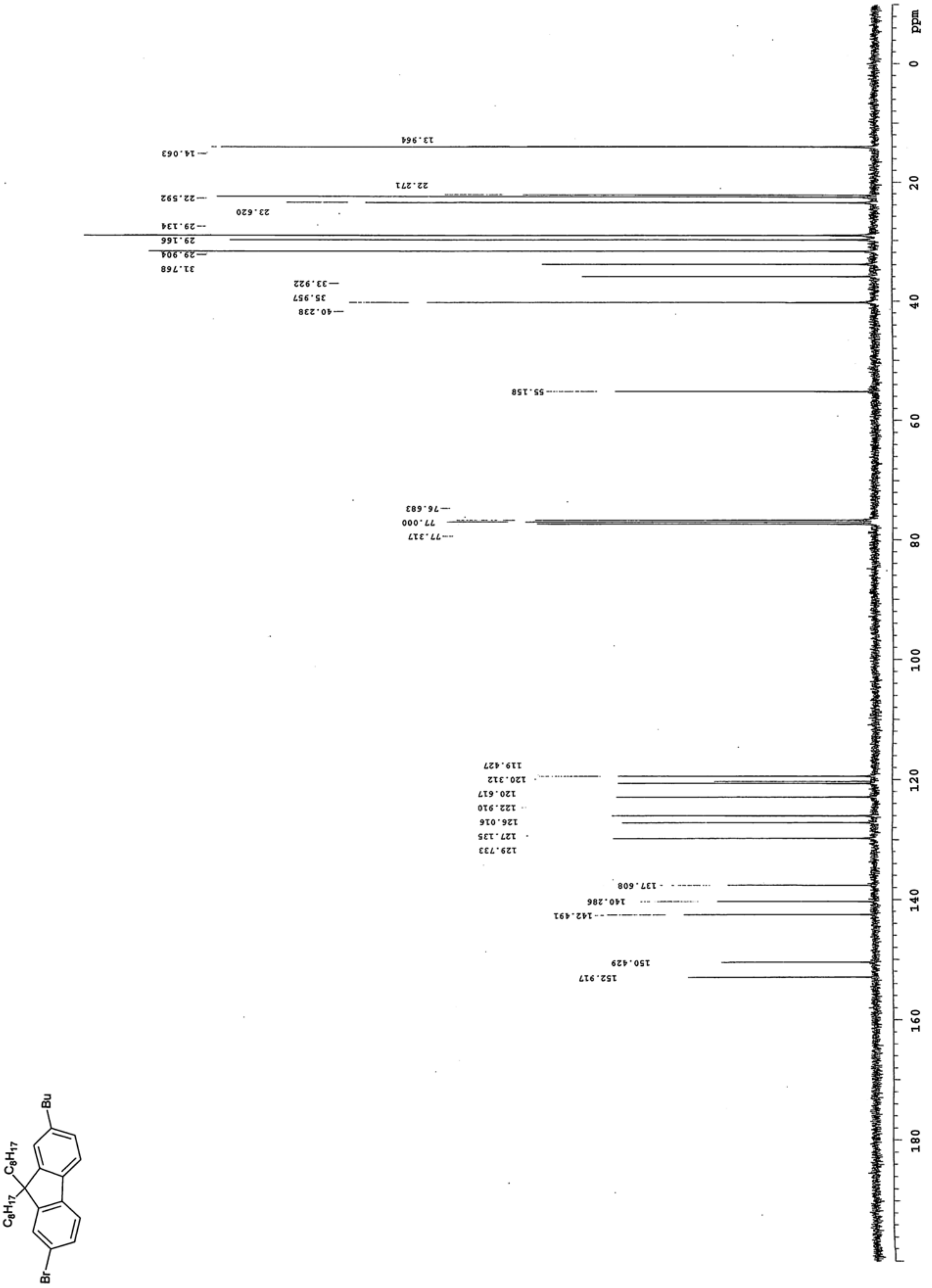

^{13}C NMR spectrum of 2-bromo-7-butyl-9,9-dioctylfluorene (31)

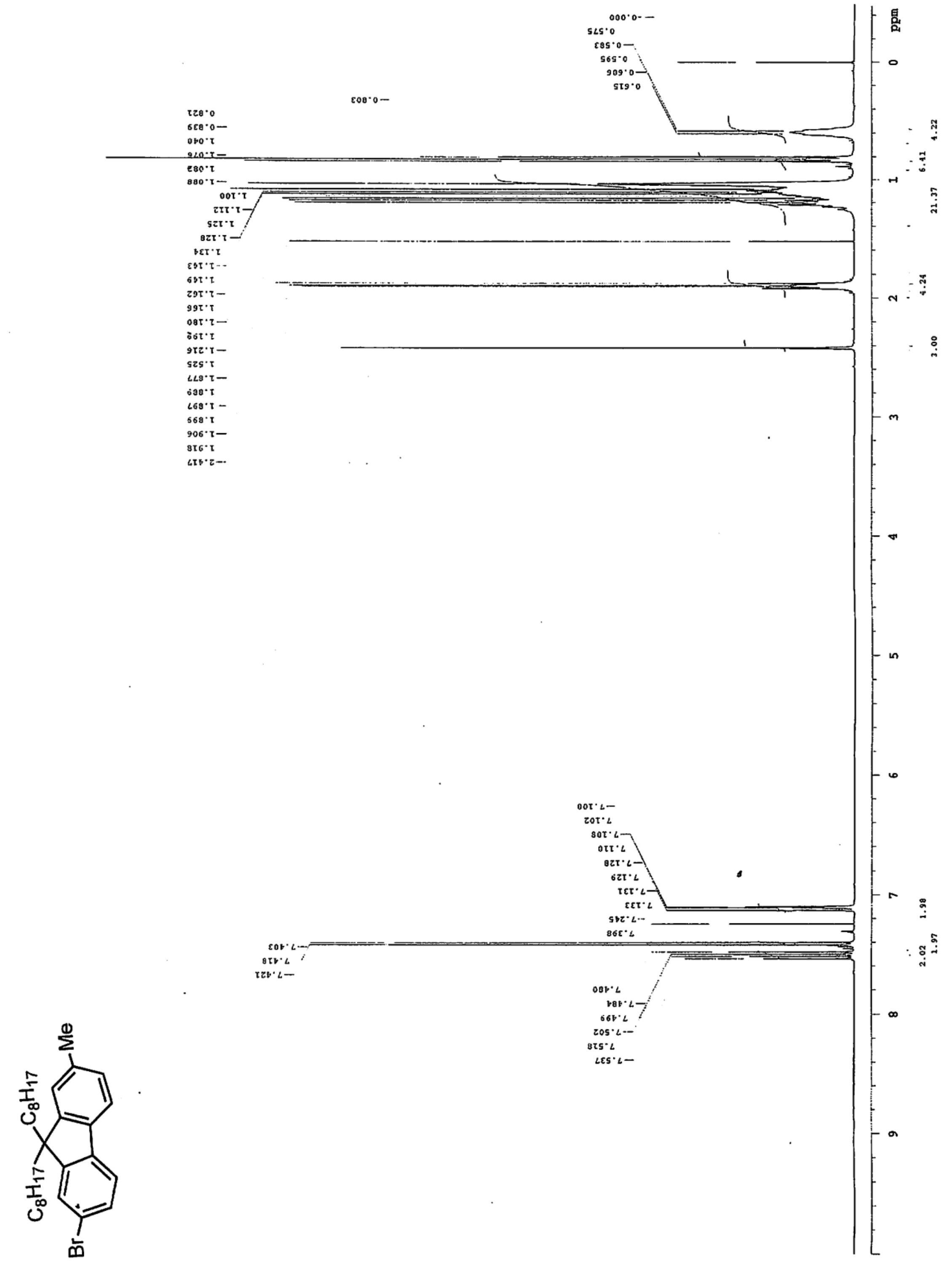

^{1}H NMR spectrum of 2-bromo-7-methyl-9,9-dioctylfluorene (32)

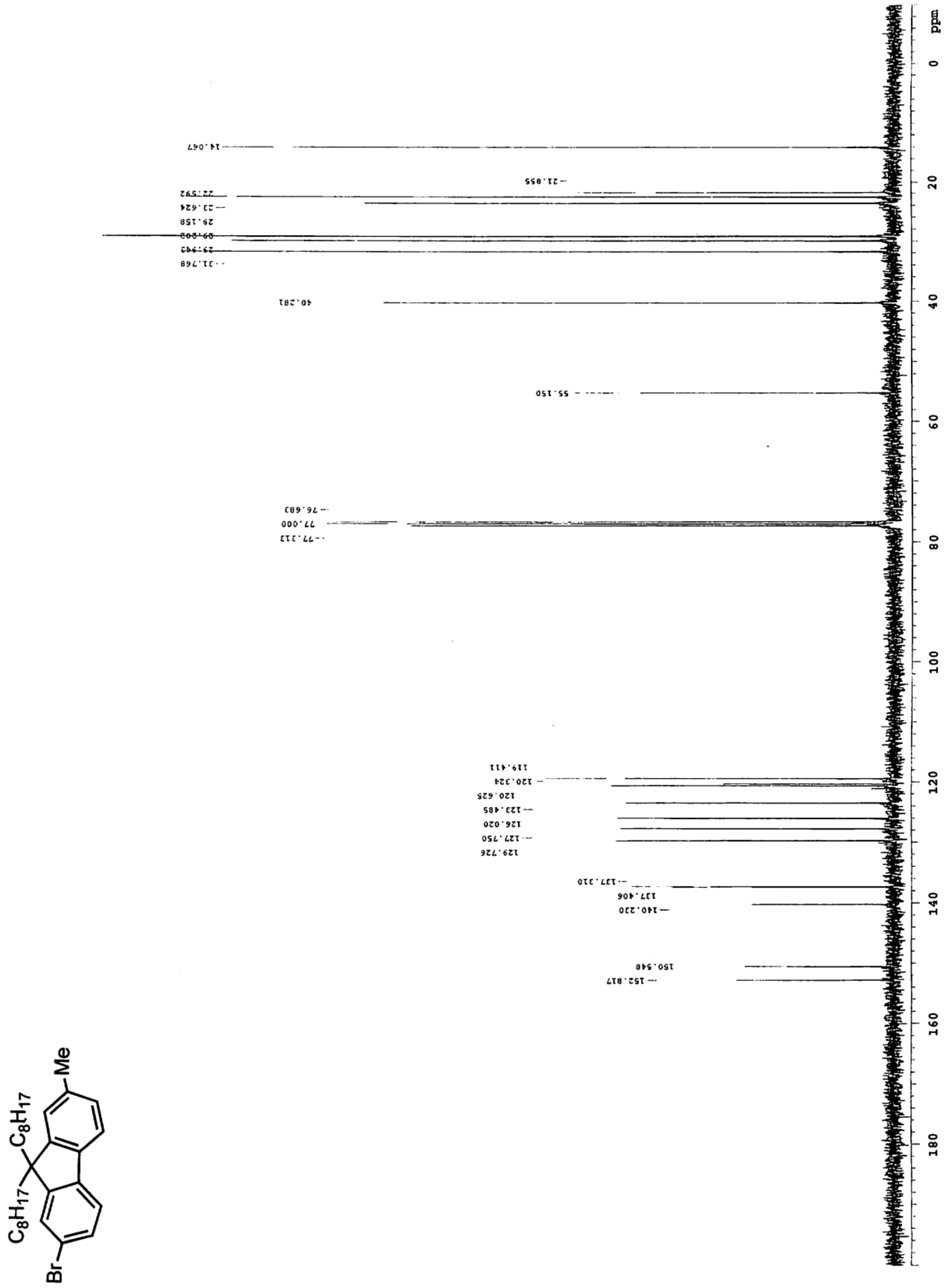

^{13}C NMR spectrum of 2-bromo-7-methyl-9,9-dioctylfluorene (32)

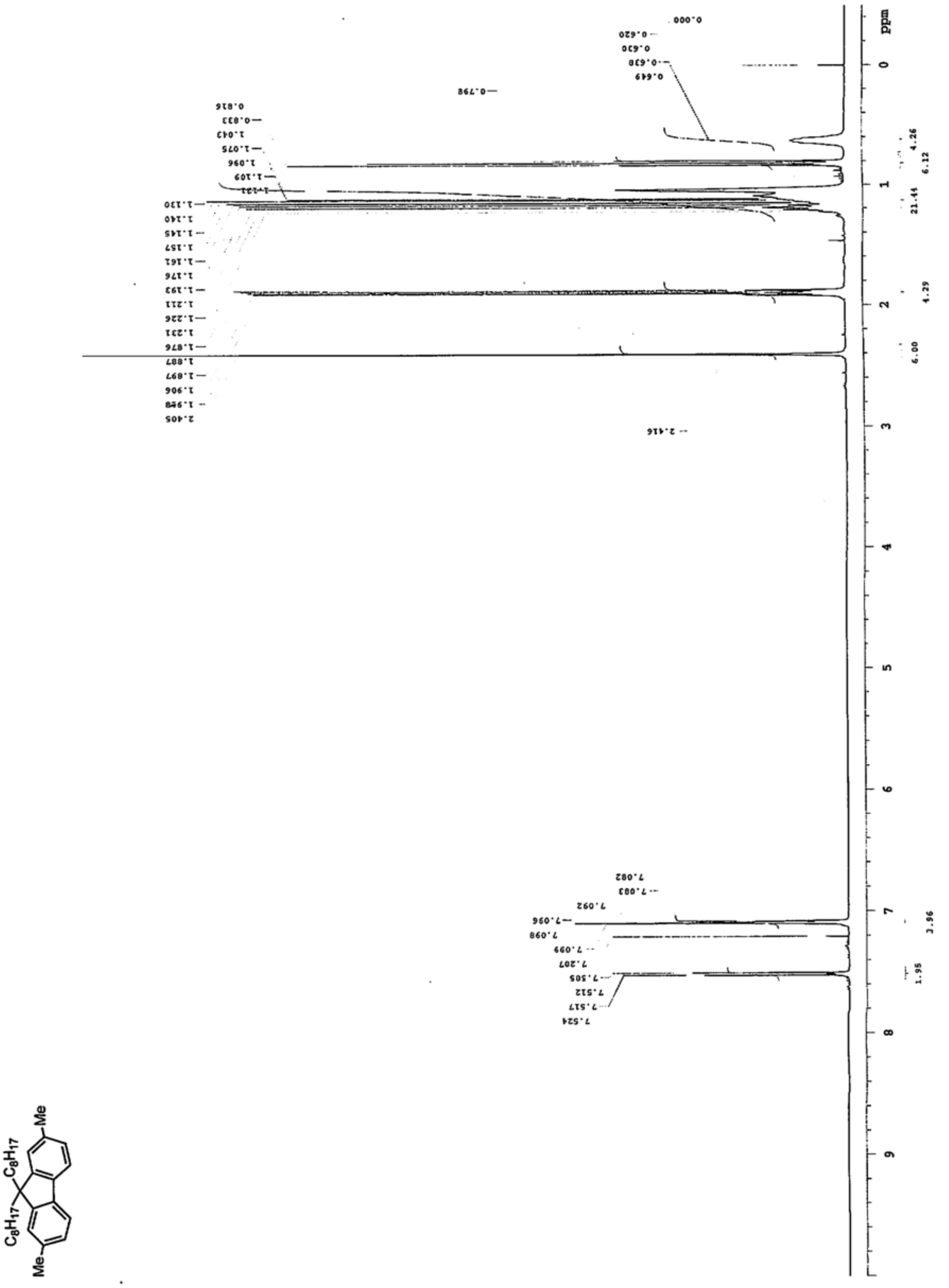

^{1}H NMR spectrum of 2,7-dimethyl-9,9-dioctylfluorene (33)

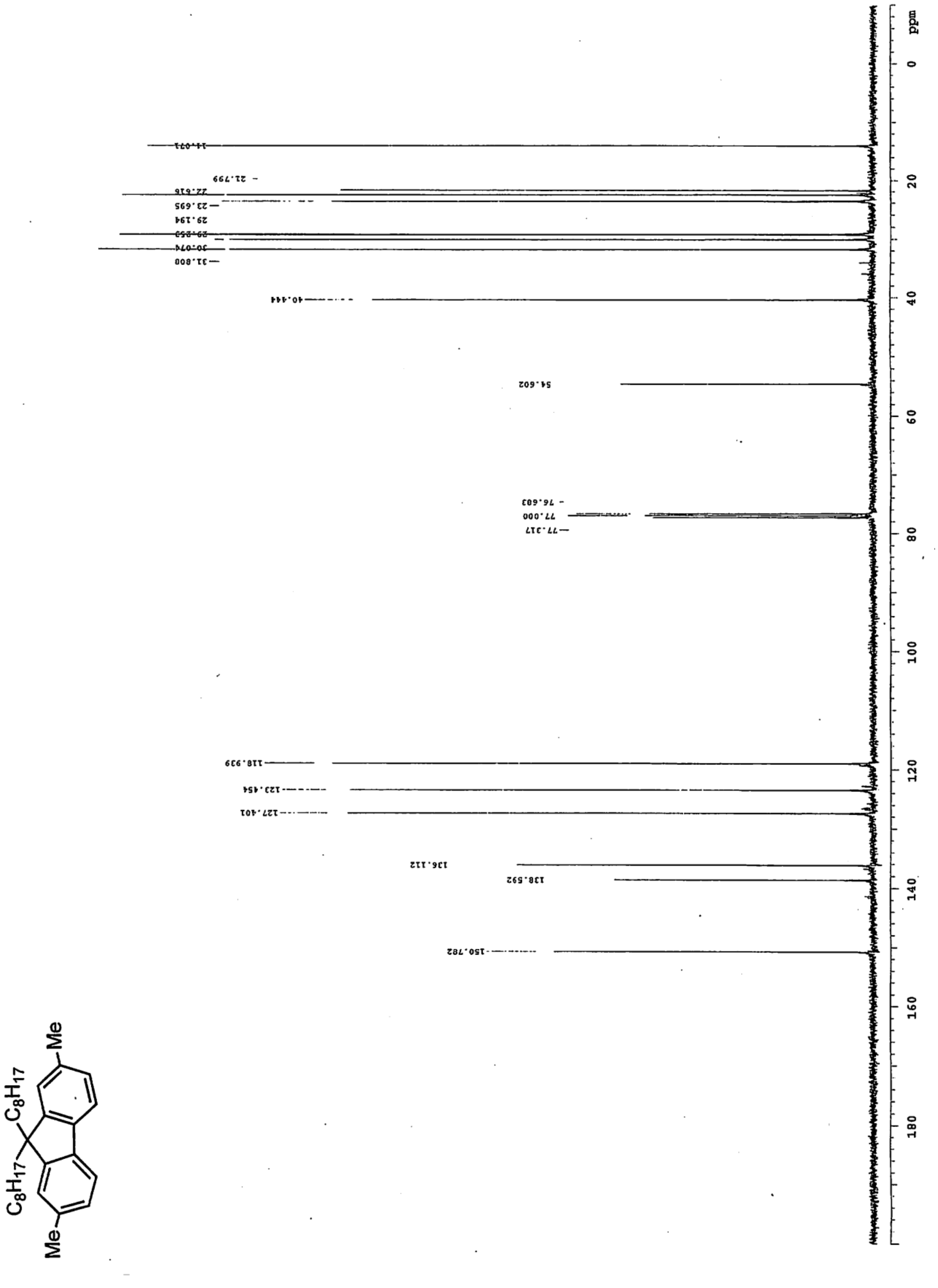

^{13}C NMR spectrum of 2,7-dimethyl-9,9-dioctylfluorene (33)

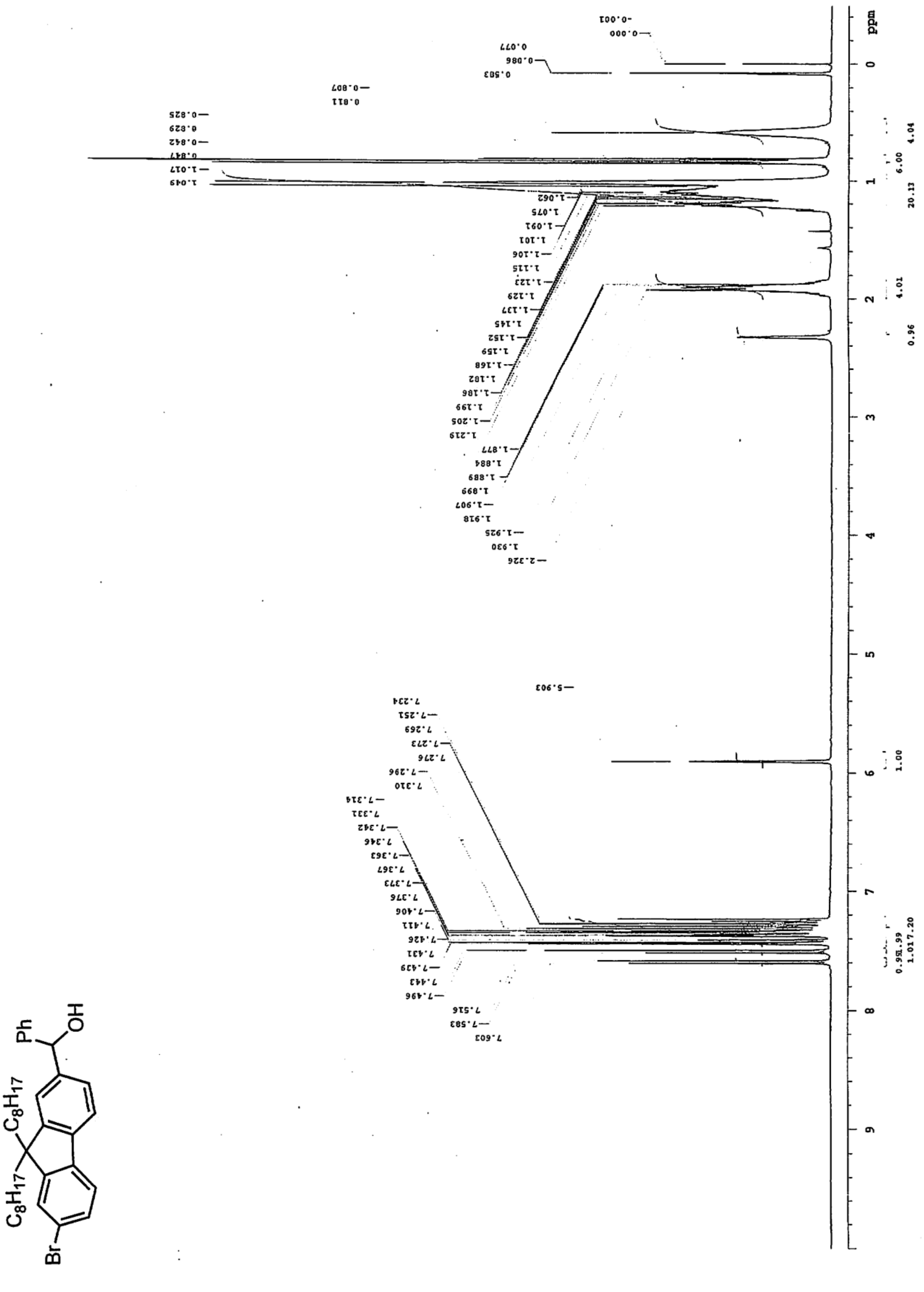

^{1}H NMR spectrum of 2-bromo-7-[hydroxyl(phenyl)methyl]-9,9-dioctylfluorene (**34**)

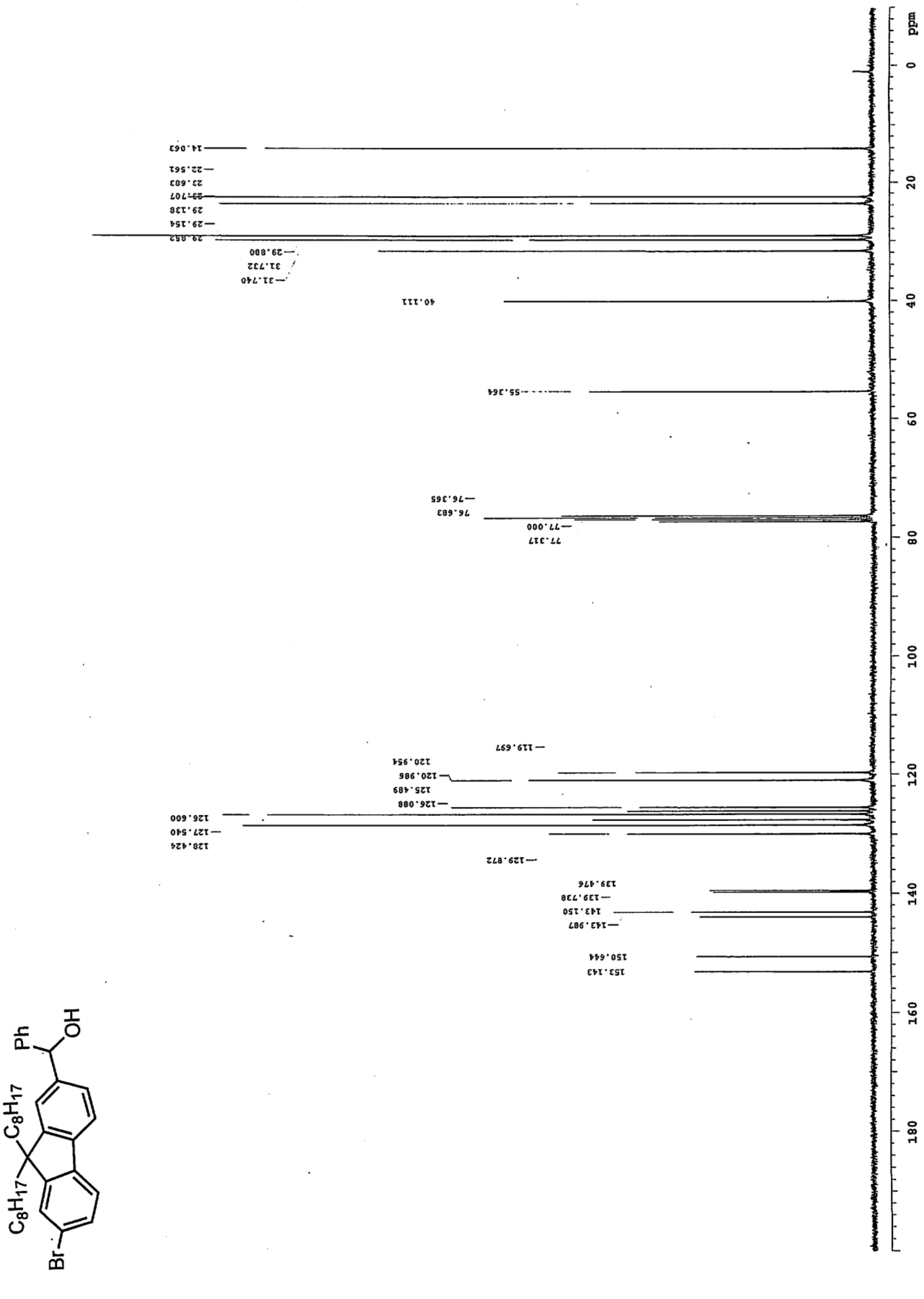

^{13}C NMR spectrum of 2-bromo-7-[hydroxyl(phenyl)methyl]-9,9-dioctylfluorene (34)

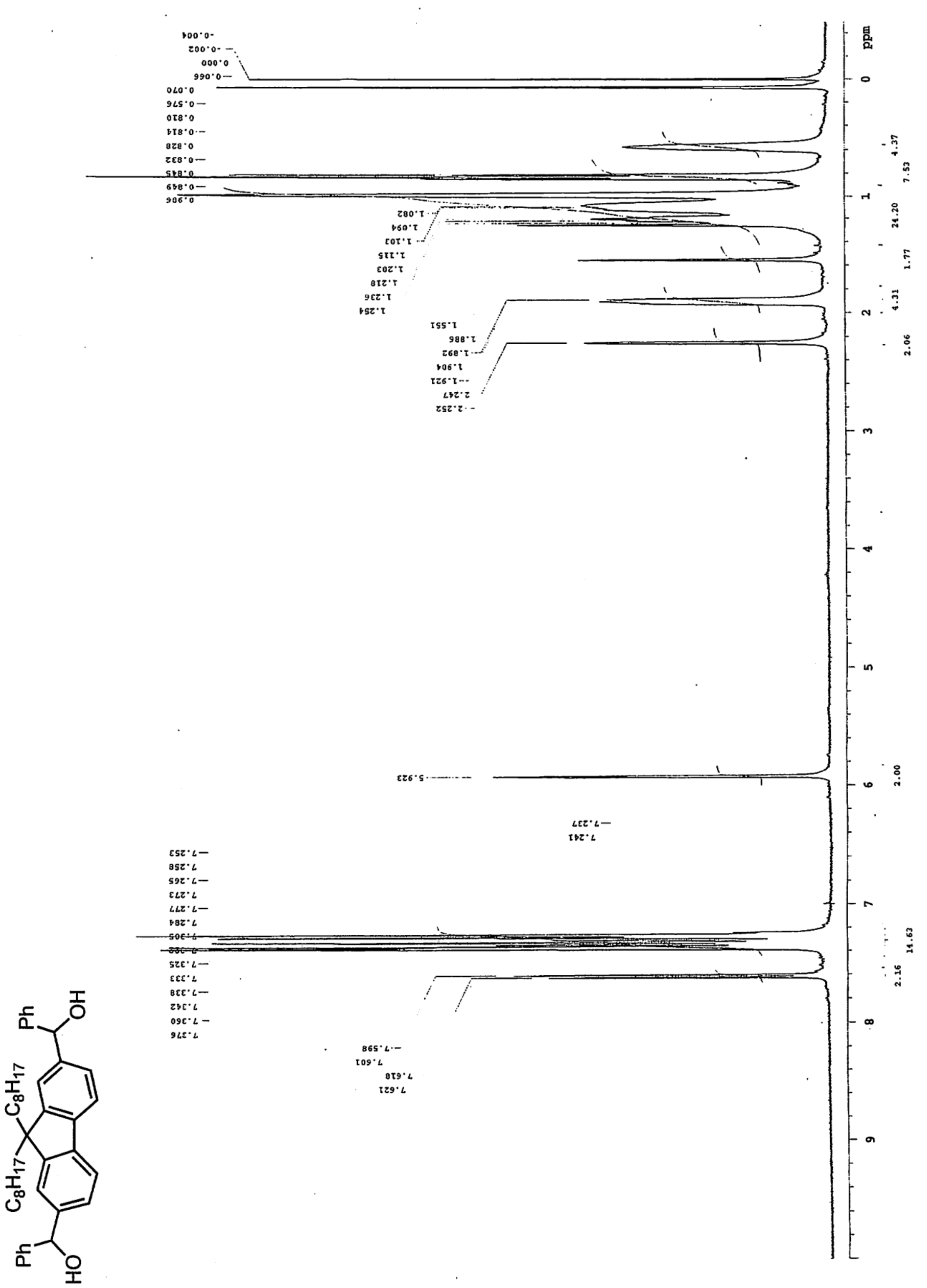

^{1}H NMR spectrum of 2,7’-di[hydroxyl(phenyl)methyl]-9,9-dioctylfluorene (35)

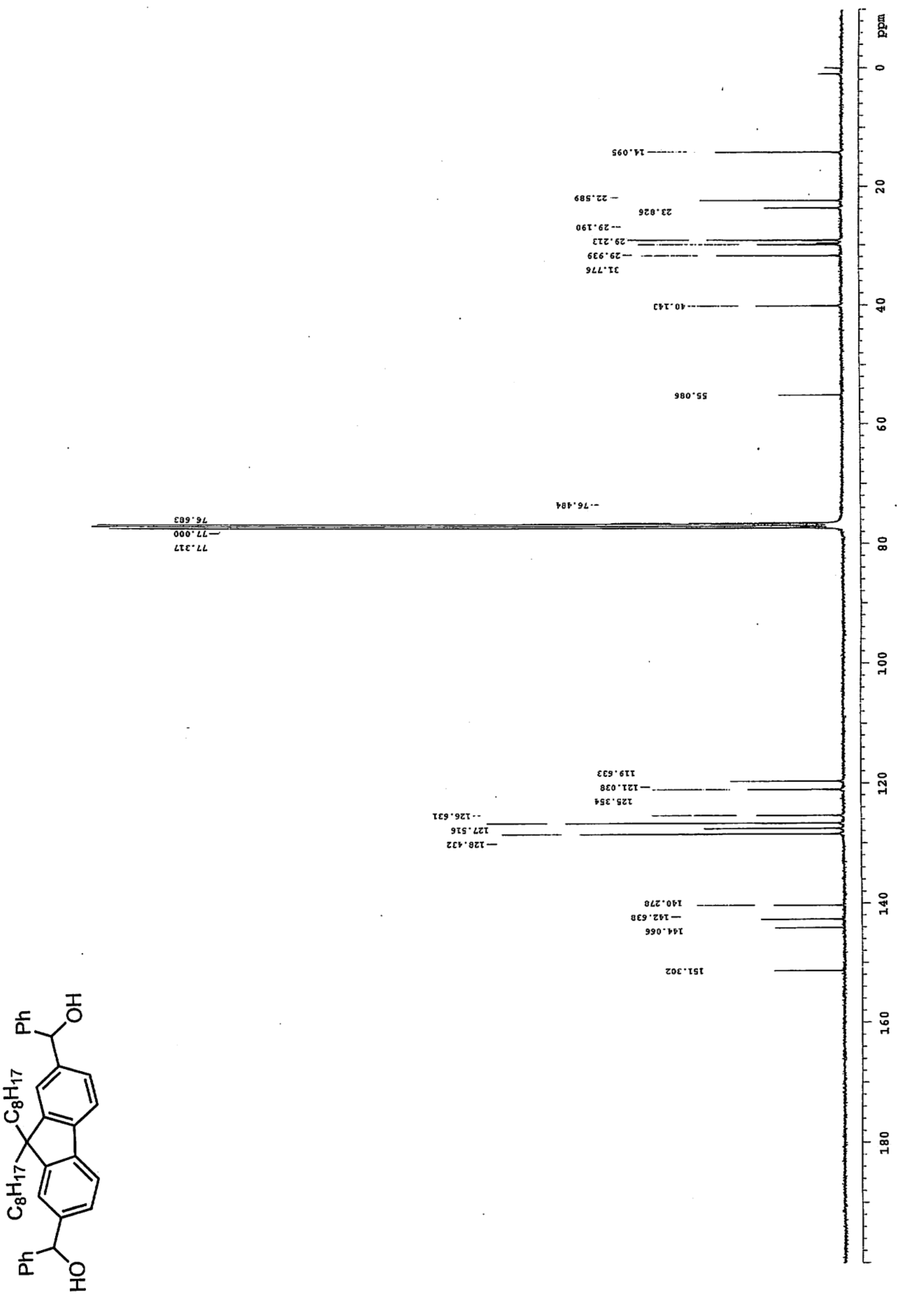

^{13}C NMR spectrum of 2,7’-di[hydroxyl(phenyl)methyl]-9,9-dioctylfluorene (35)

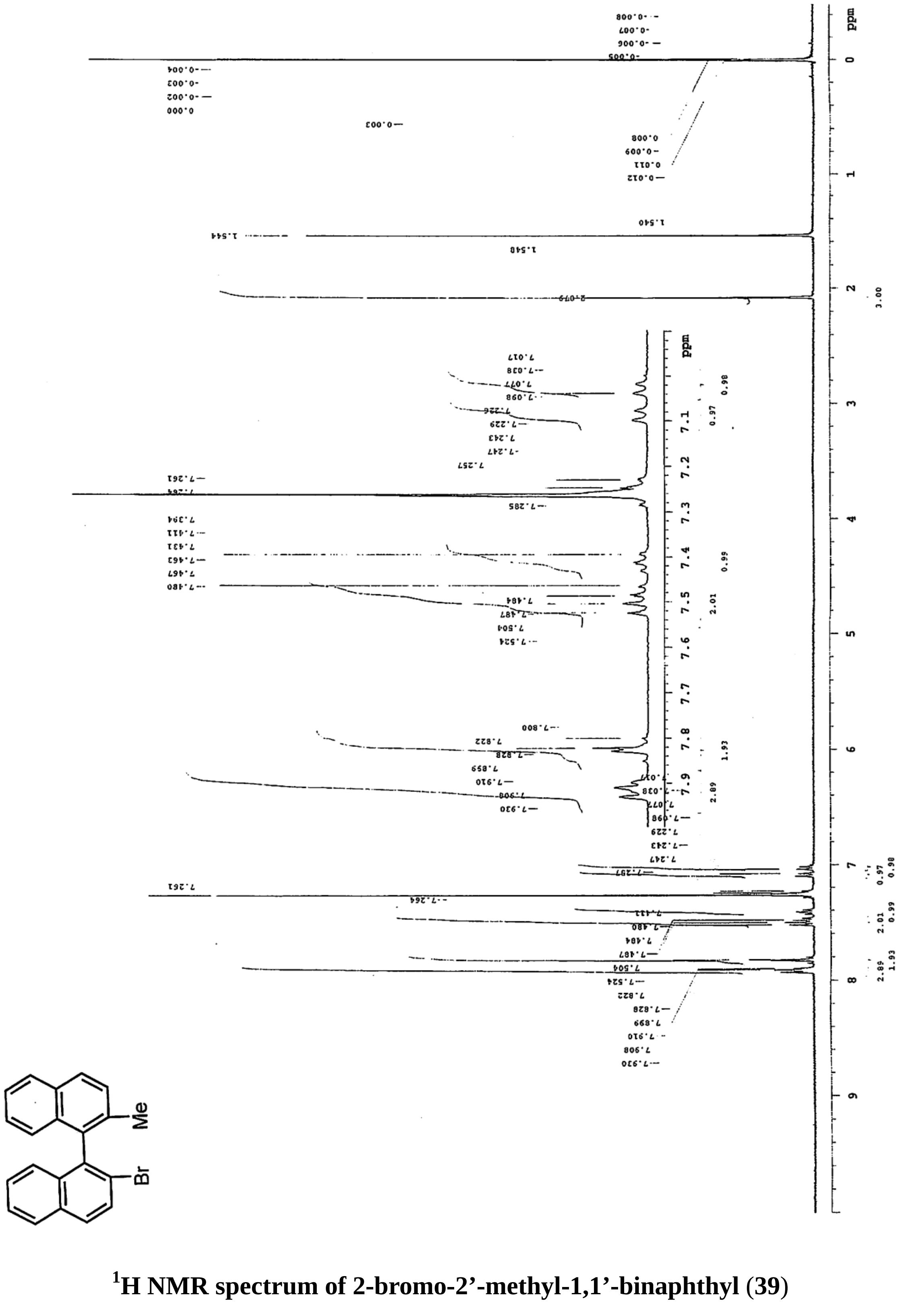

^{1}H NMR spectrum of 2-bromo-2'-methyl-1,1'-binaphthyl (39)

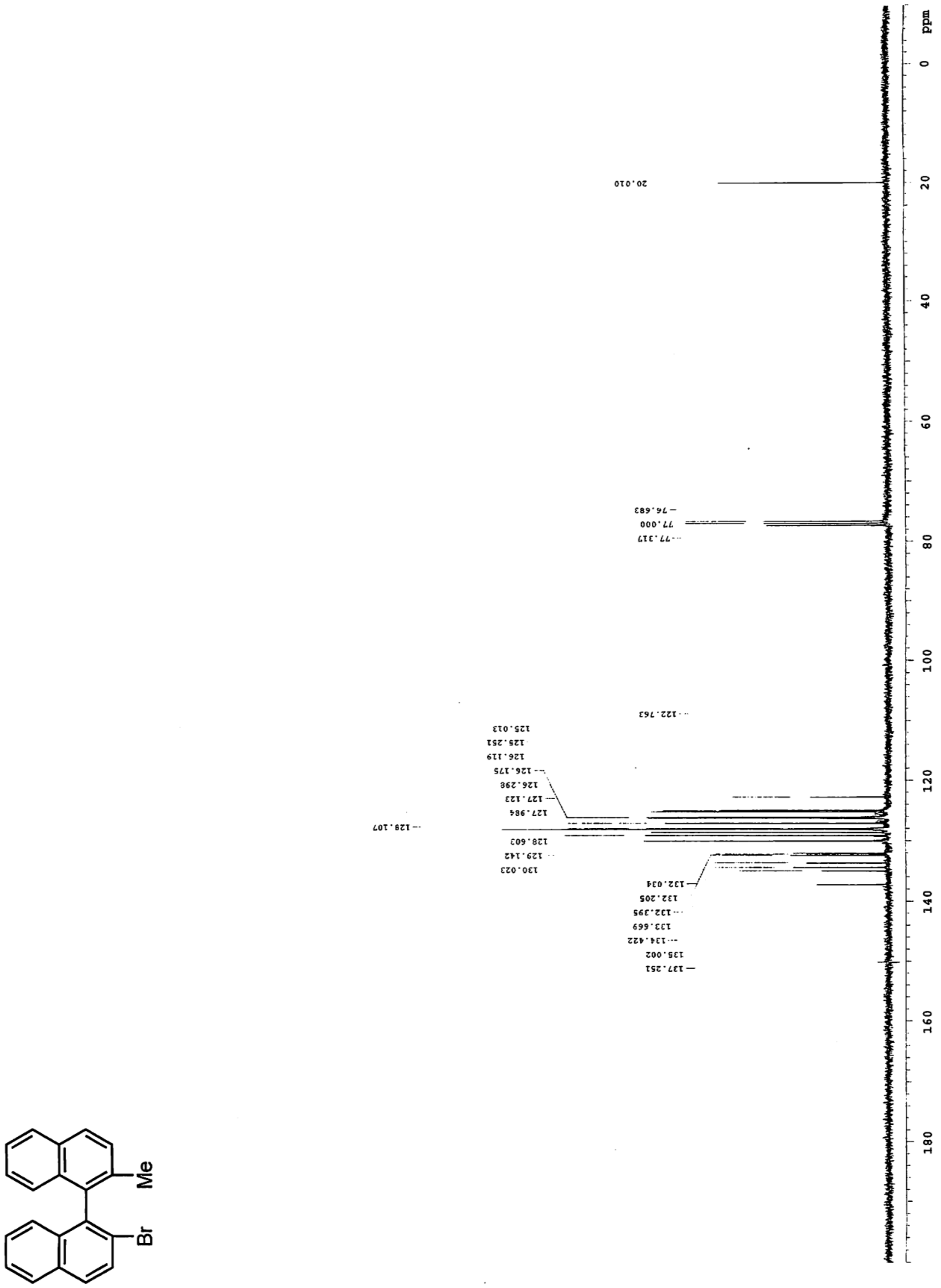

^{13}C NMR spectrum of 2-bromo-2’-methyl-1,1’-binaphthyl (39)
