# Supplementary material for: Synthesis of unsymmetrically substituted biaryls via sequential lithiation of dibromobiaryls using integrated microflow systems
Source: Beilstein J Org Chem. 2009 Apr 29;5:16. doi: 10.3762/bjoc.5.16 (PMC2686311; doi:10.3762/bjoc.5.16)
Supplement: File 3 — 1H NMR spectra and 13C NMR spectra of compounds 41, 42, 46, 48–52. [file Beilstein_J_Org_Chem-05-16-s003.pdf]

Synthesis of unsymmetrically substituted biaryls via sequential lithiation of dibromobiaryls using integrated microflow systems

Aiichiro Nagaki, Naofumi Takabayashi, Yutaka Tomida, and Jun-ichi Yoshida*

Address: Department of Synthetic Chemistry and Biological Chemistry, Graduate School of Engineering, Kyoto University, Kyotodaigakukatsura, Nishikyo-ku, Kyoto, 615-8510, Japan

Email: Jun-ichi Yoshida - yoshida@sbchem.kyoto-u.ac.jp

* Corresponding author

Supporting Information

^{1}H NMR spectra and ^{13}C NMR spectra of compounds 41–42,46,48–52

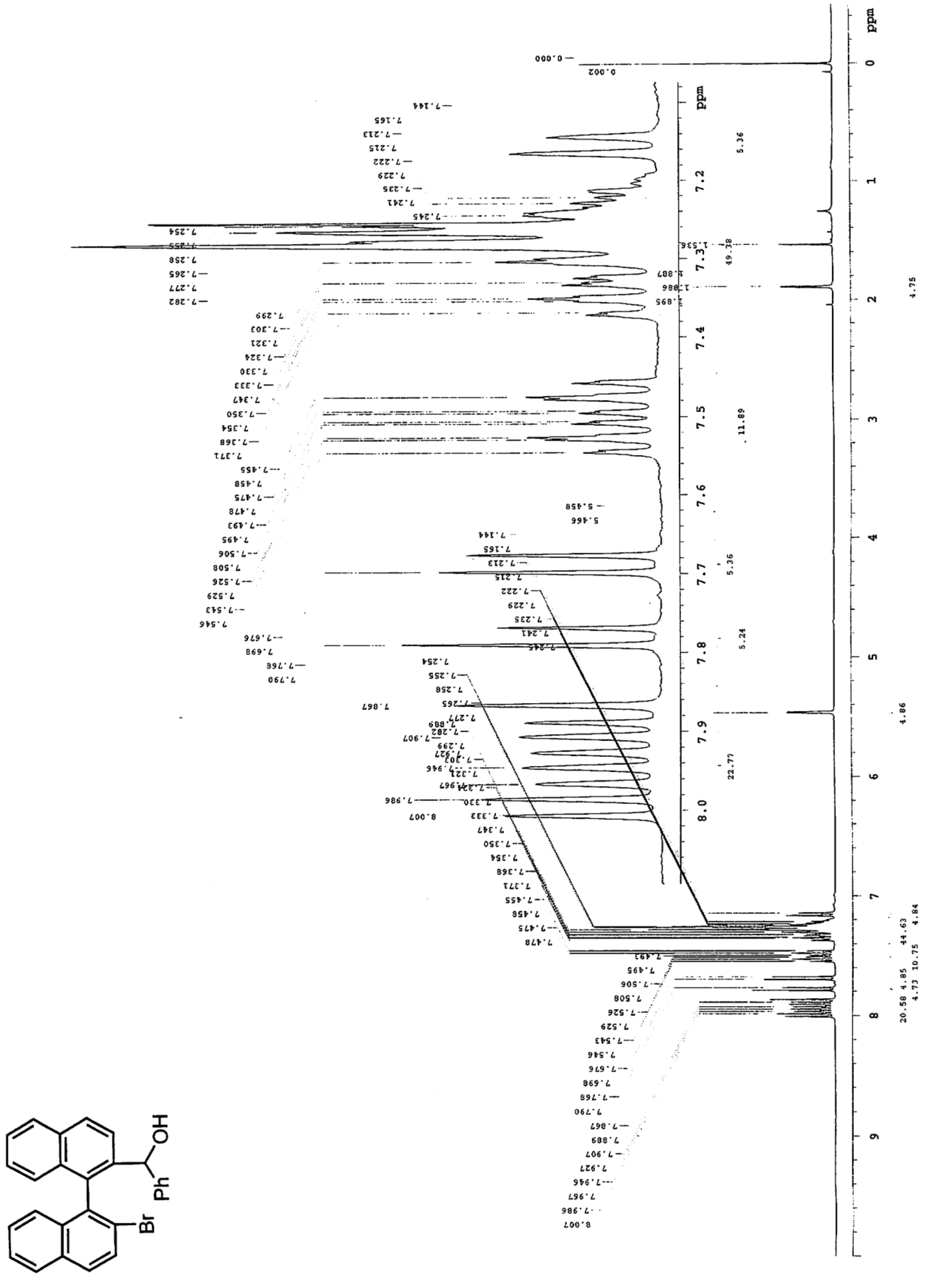

^{1}H NMR spectrum of the major diastereomer of 2-bromo-2’-[hydroxyl(phenyl)methyl]-1,1’-binaphthyl (41)

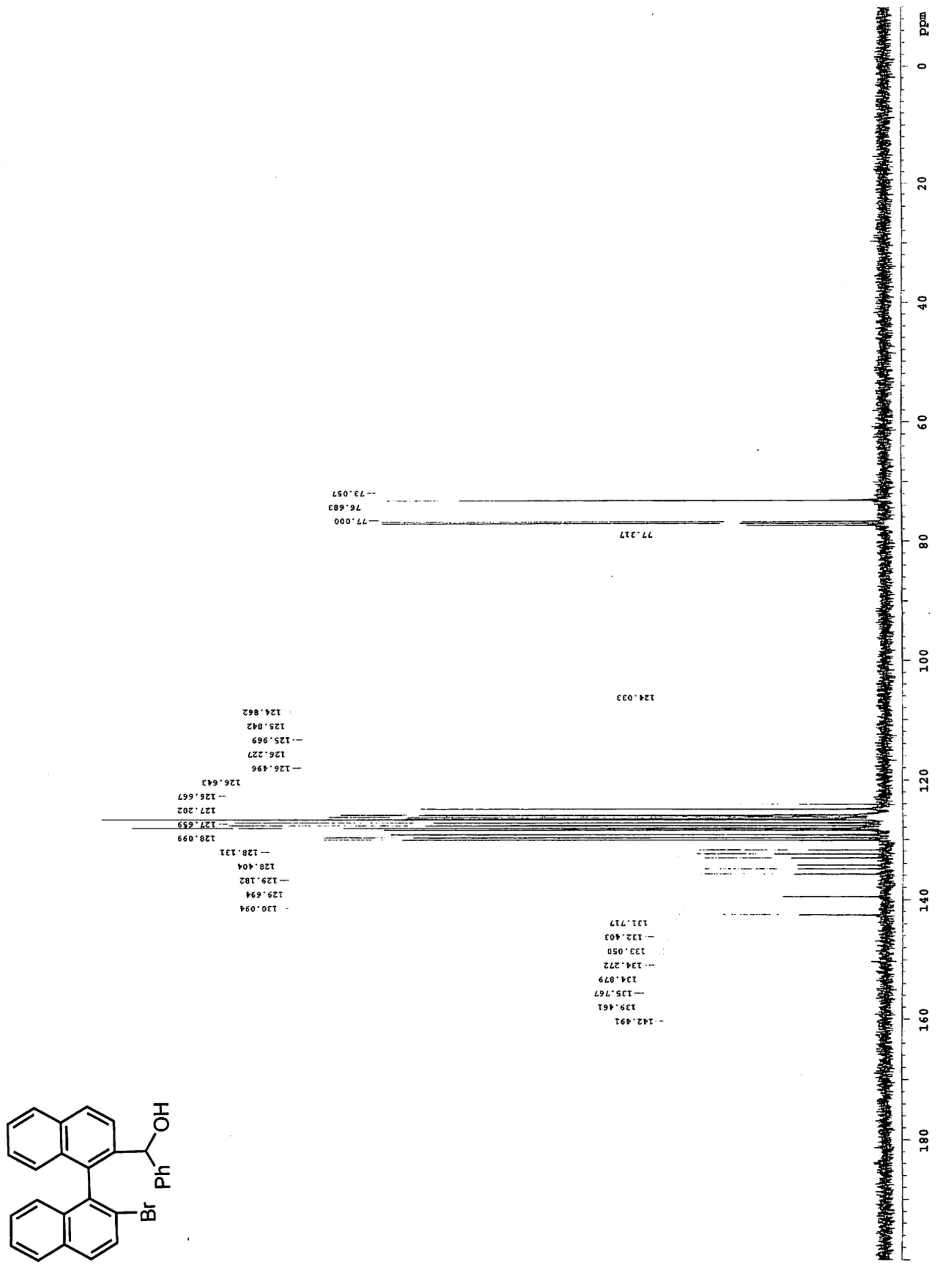

^{13}C NMR spectrum of the major diastereomer of 2-bromo-2’-[hydroxyl(phenyl)methyl]-1,1’-binaphthyl (41)

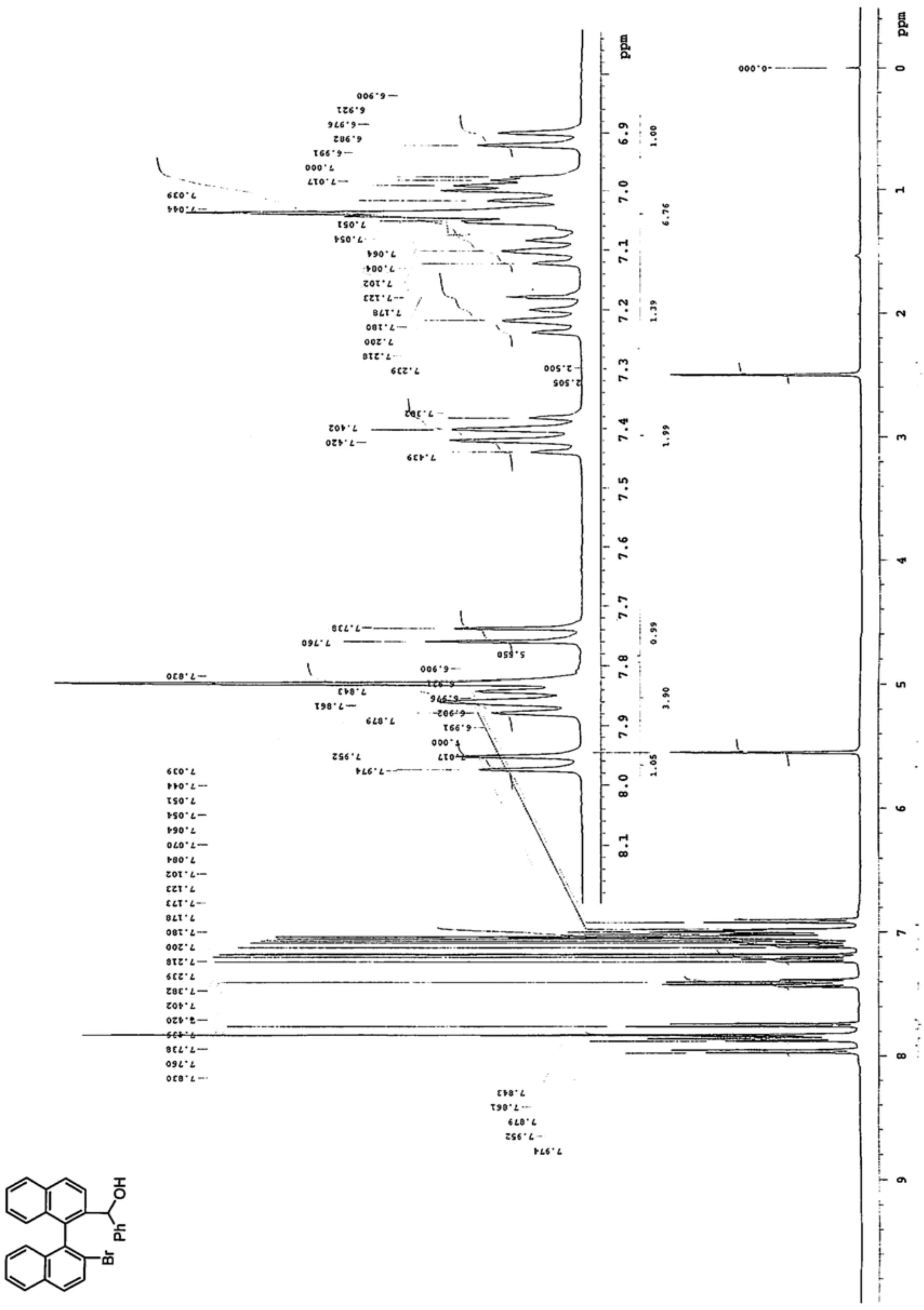

^{1}H NMR spectrum of the minor diastereomer of 2-bromo-2'-[hydroxyl(phenyl)methyl]-1,1'-binaphthyl (41)

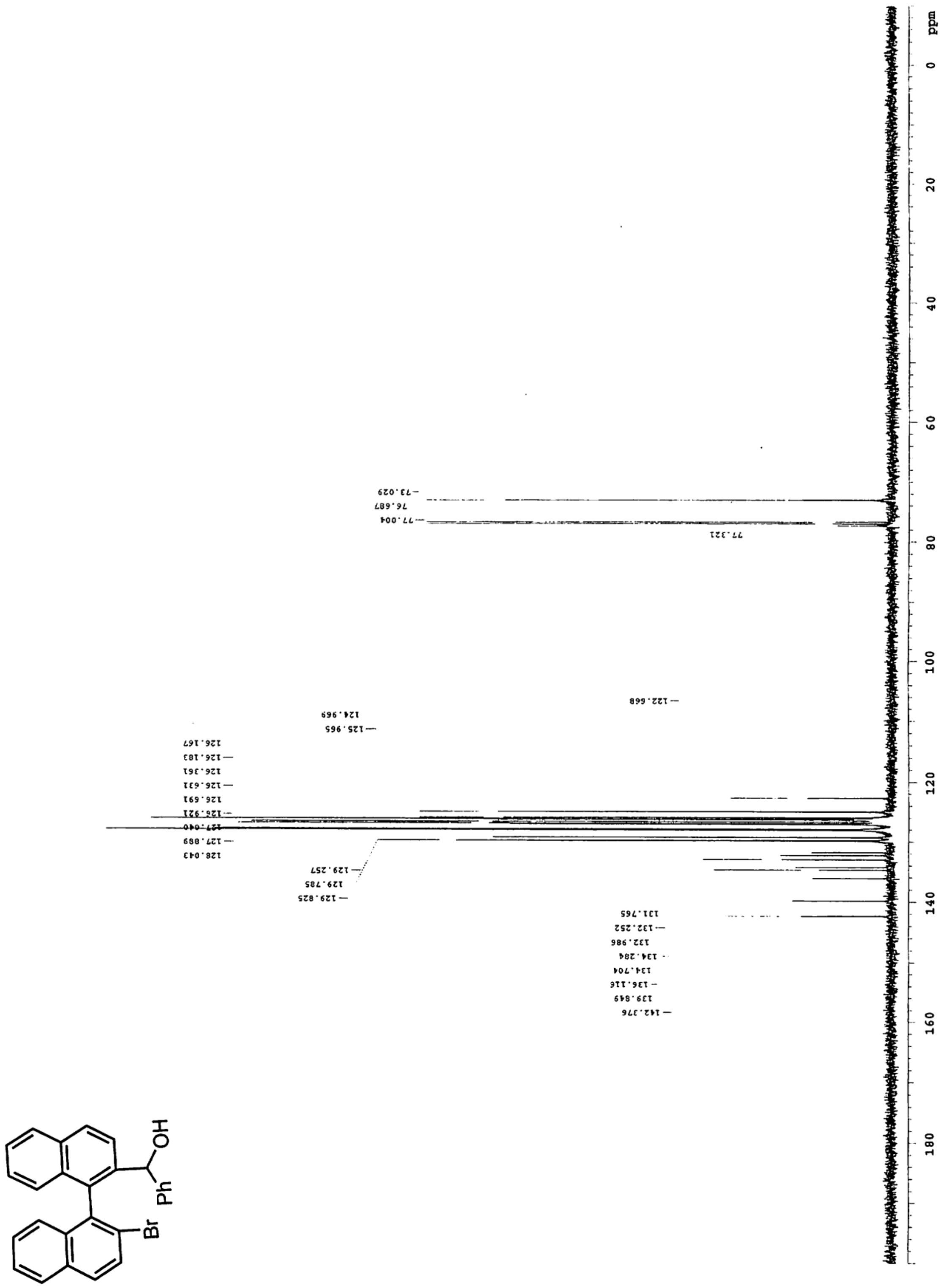

^{13}C NMR spectrum of the minor diastereomer of
2-bromo-2’-[hydroxyl(phenyl)methyl]-1,1’-binaphthyl (**41**)

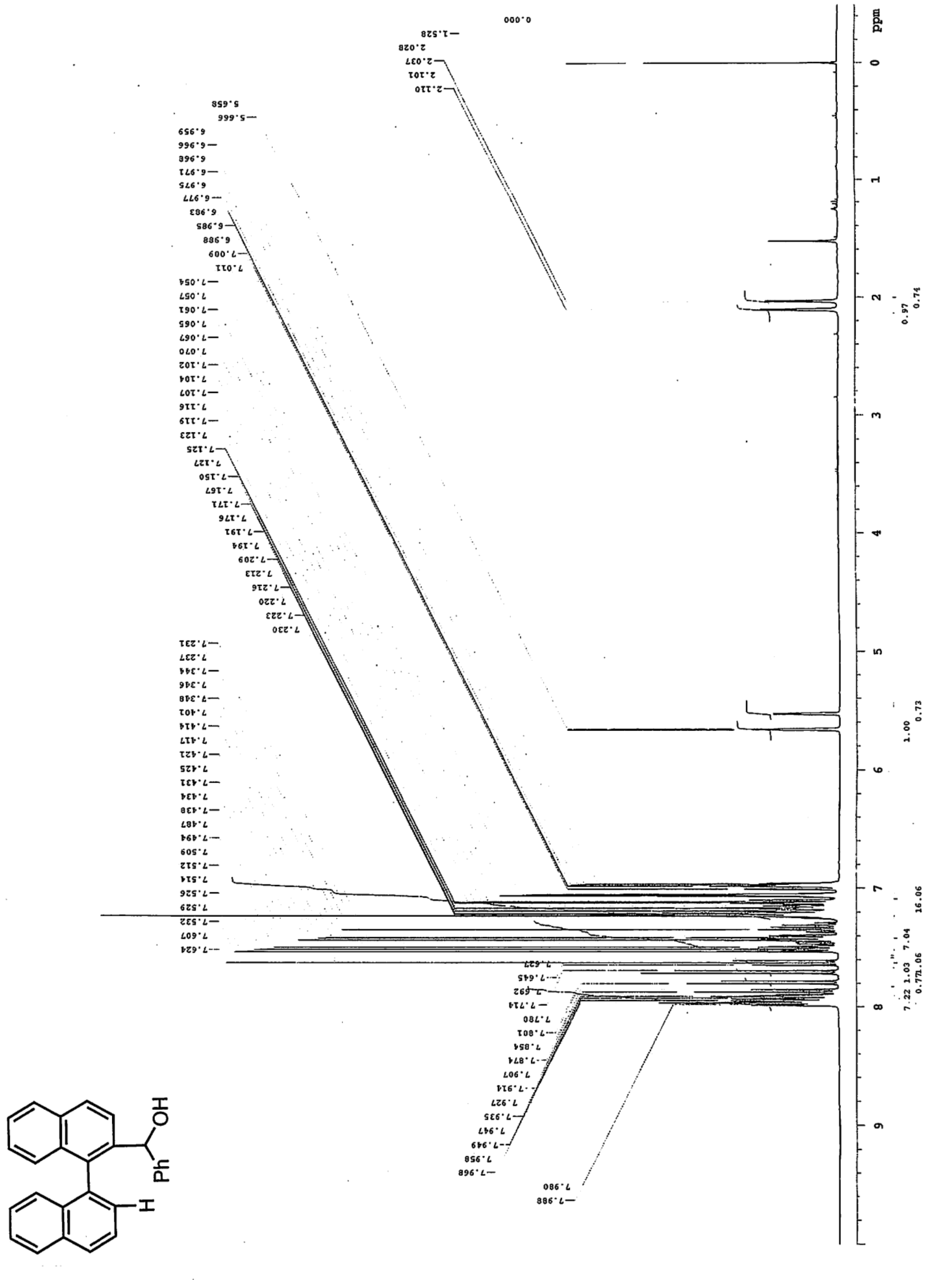

^{1}H NMR spectrum of 2-[hydroxyl(phenyl)methyl]-1,1’-binaphthyl (42)

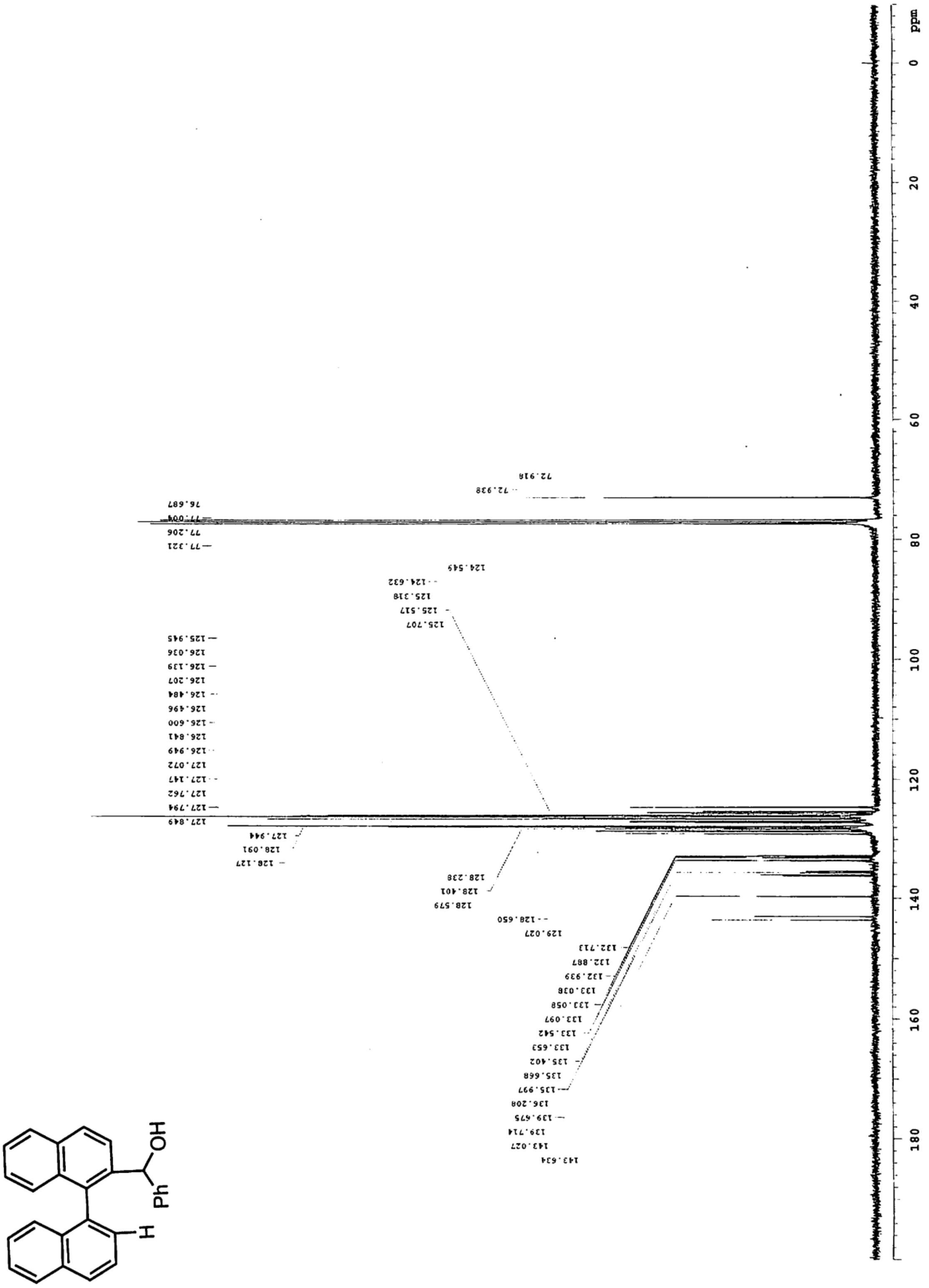

^{13}C NMR spectrum of 2-[hydroxyl(phenyl)methyl]-1,1'-binaphthyl (**42**)

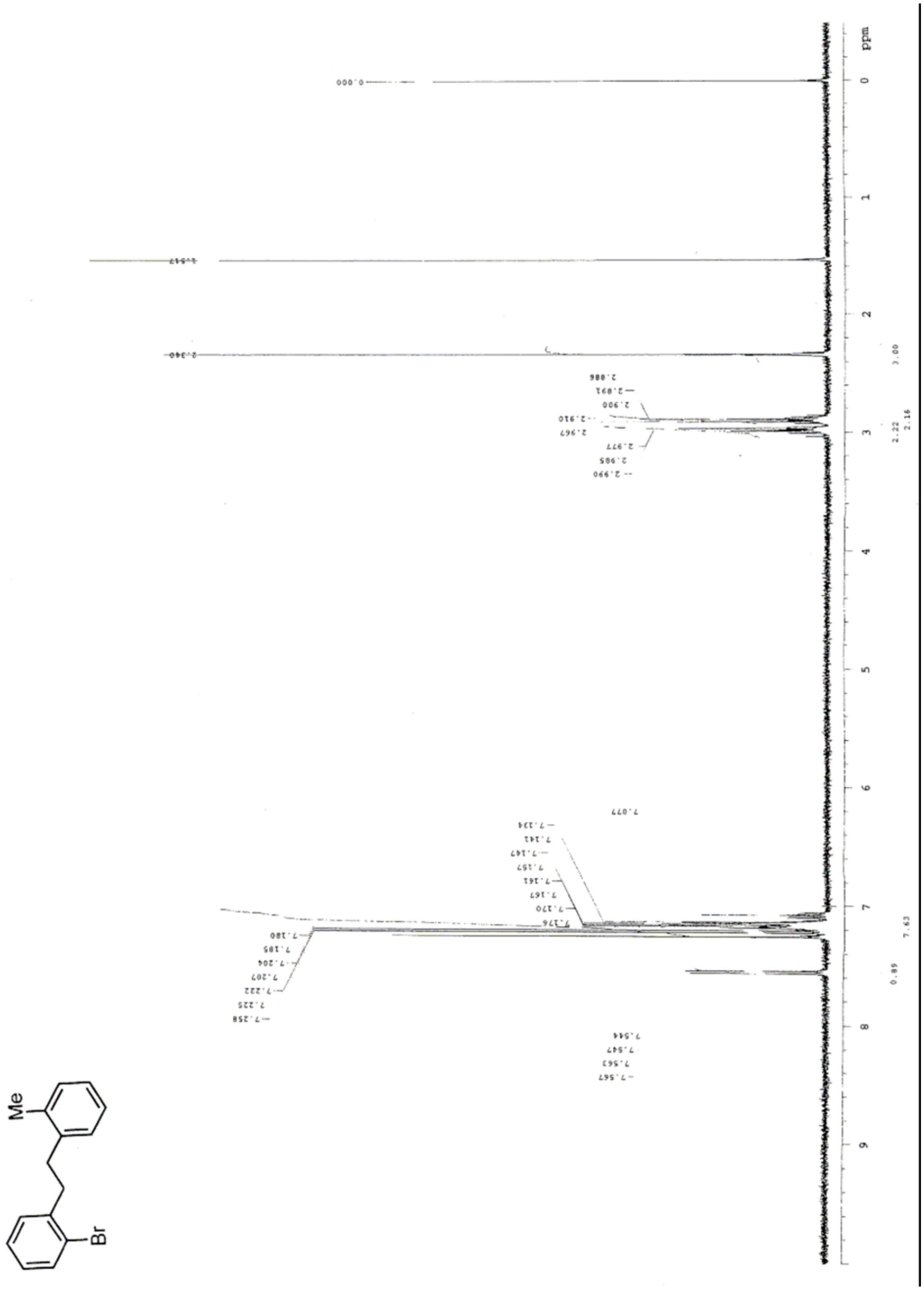

^{1}H NMR spectrum of 2-bromo-2’-methylbibenzyl (46)

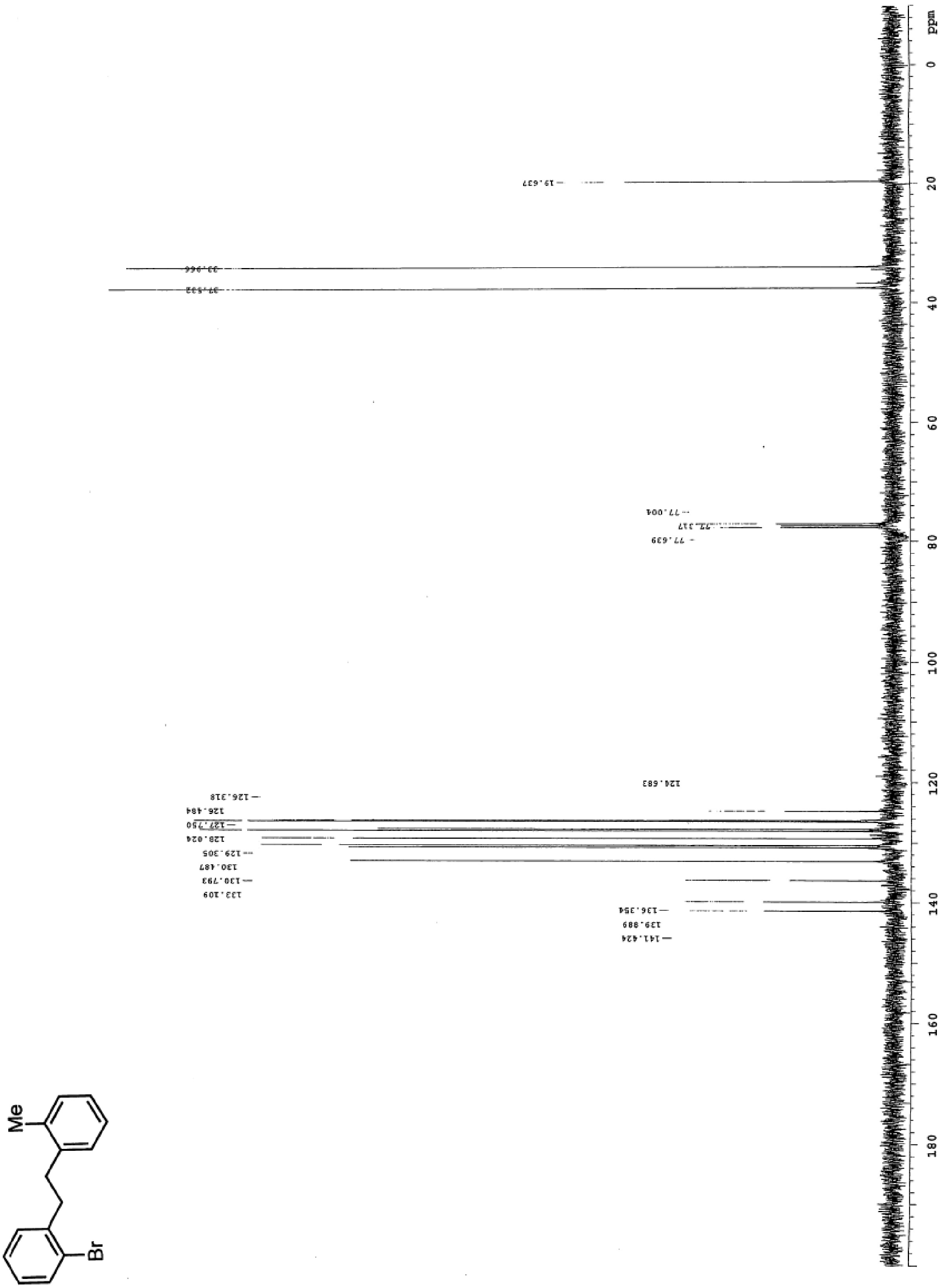

^{13}C NMR spectrum of 2-bromo-2’-methylbibenzyl (46)

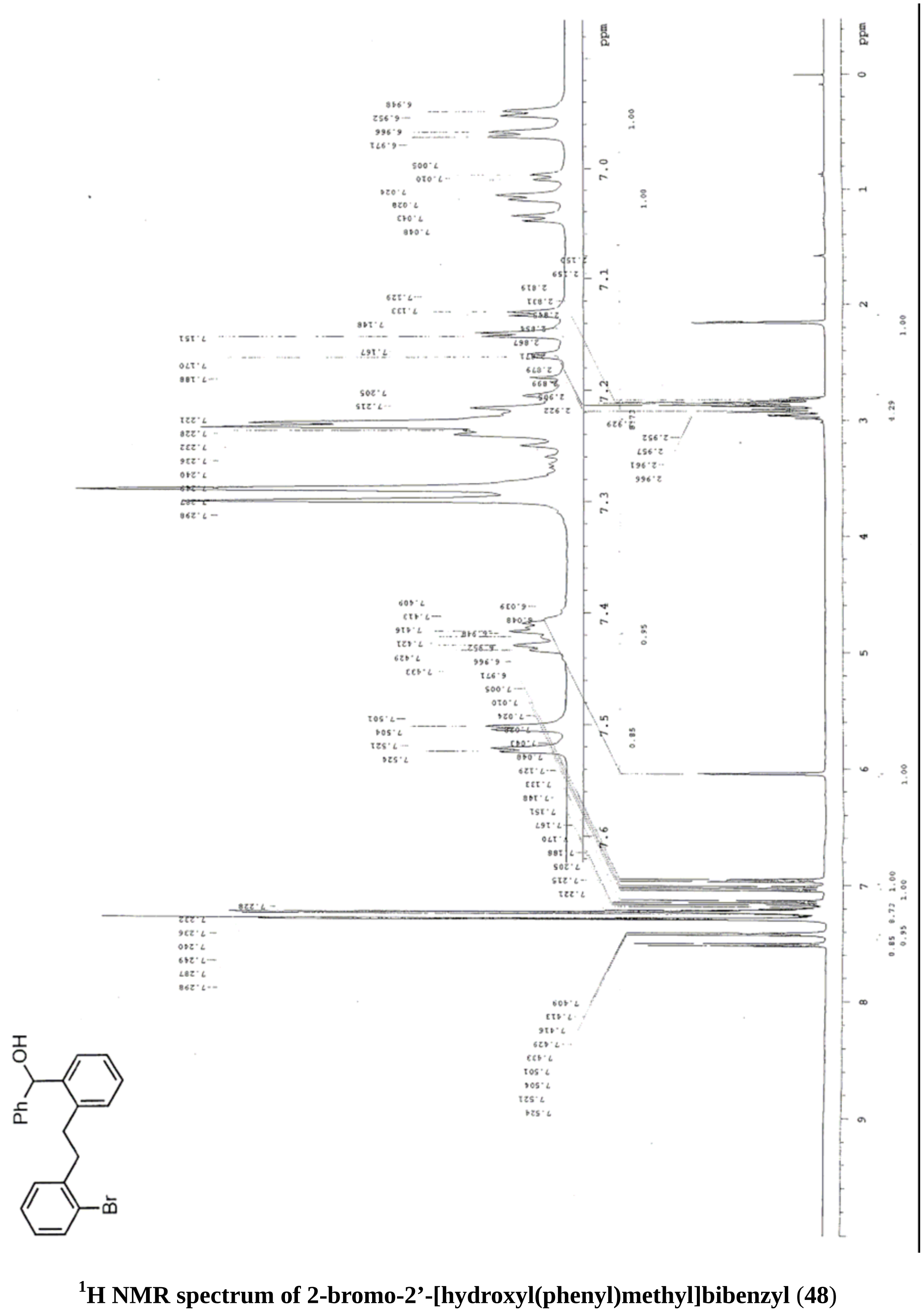

^{1}H NMR spectrum of 2-bromo-2’-[hydroxyl(phenyl)methyl]bibenzyl (48)

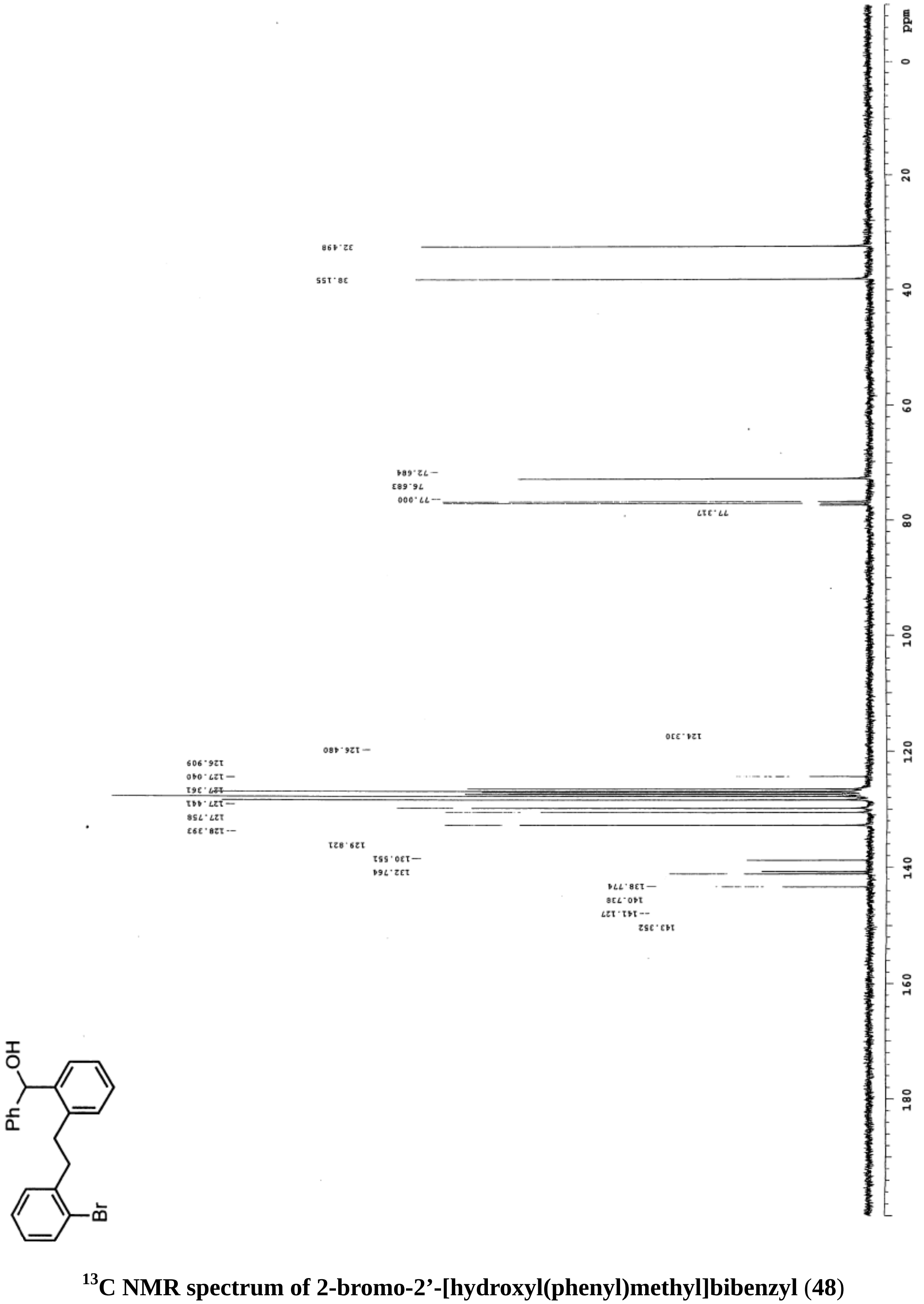

^{13}C NMR spectrum of 2-bromo-2’-[hydroxyl(phenyl)methyl]bibenzyl (48)

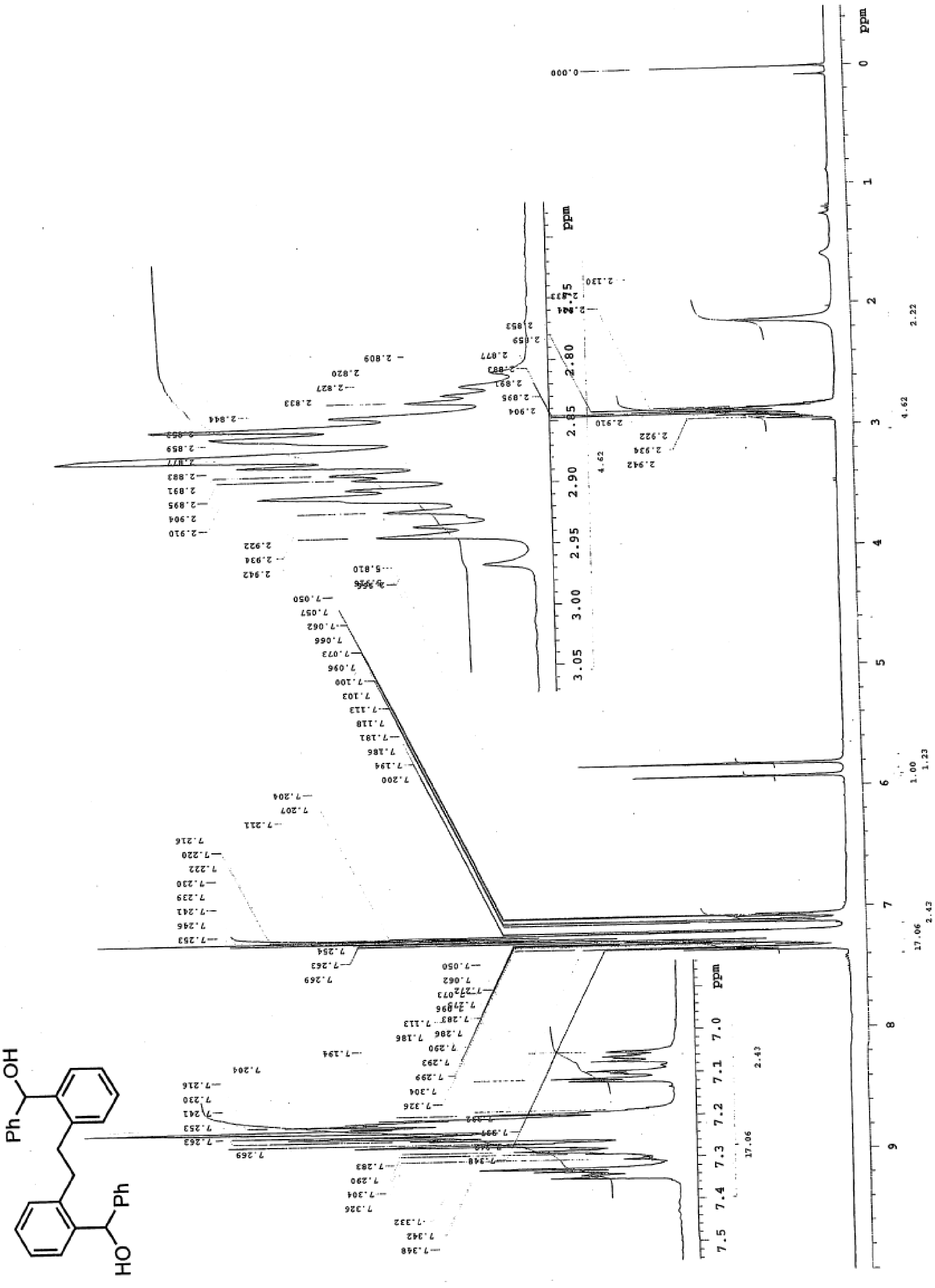

^{1}H NMR spectrum of 2,2’-di[hydroxyl(phenyl)methyl]bibenzyl (49)

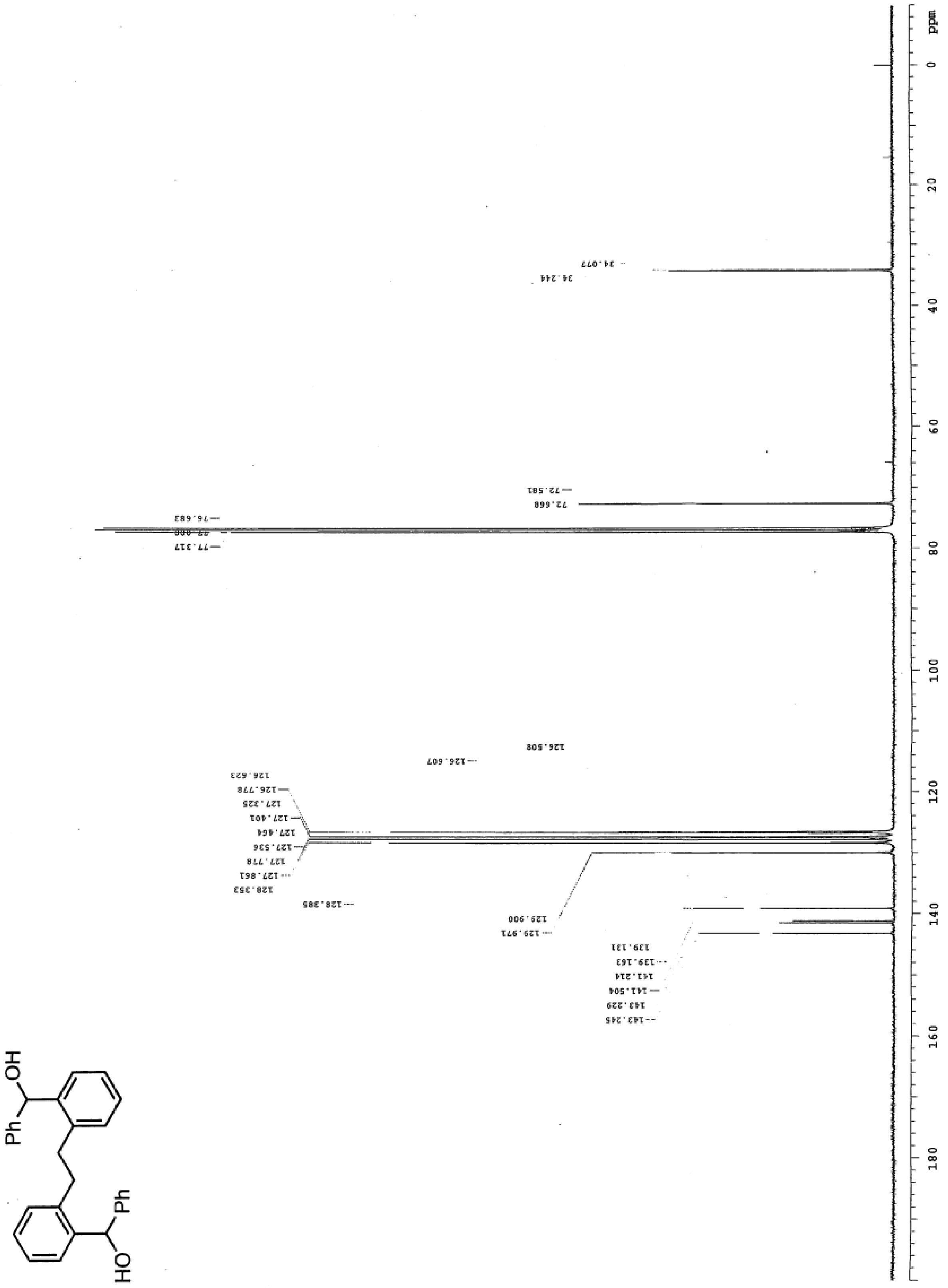

^{13}C NMR spectrum of 2,2'-di[hydroxyl(phenyl)methyl]bibenzyl (49)

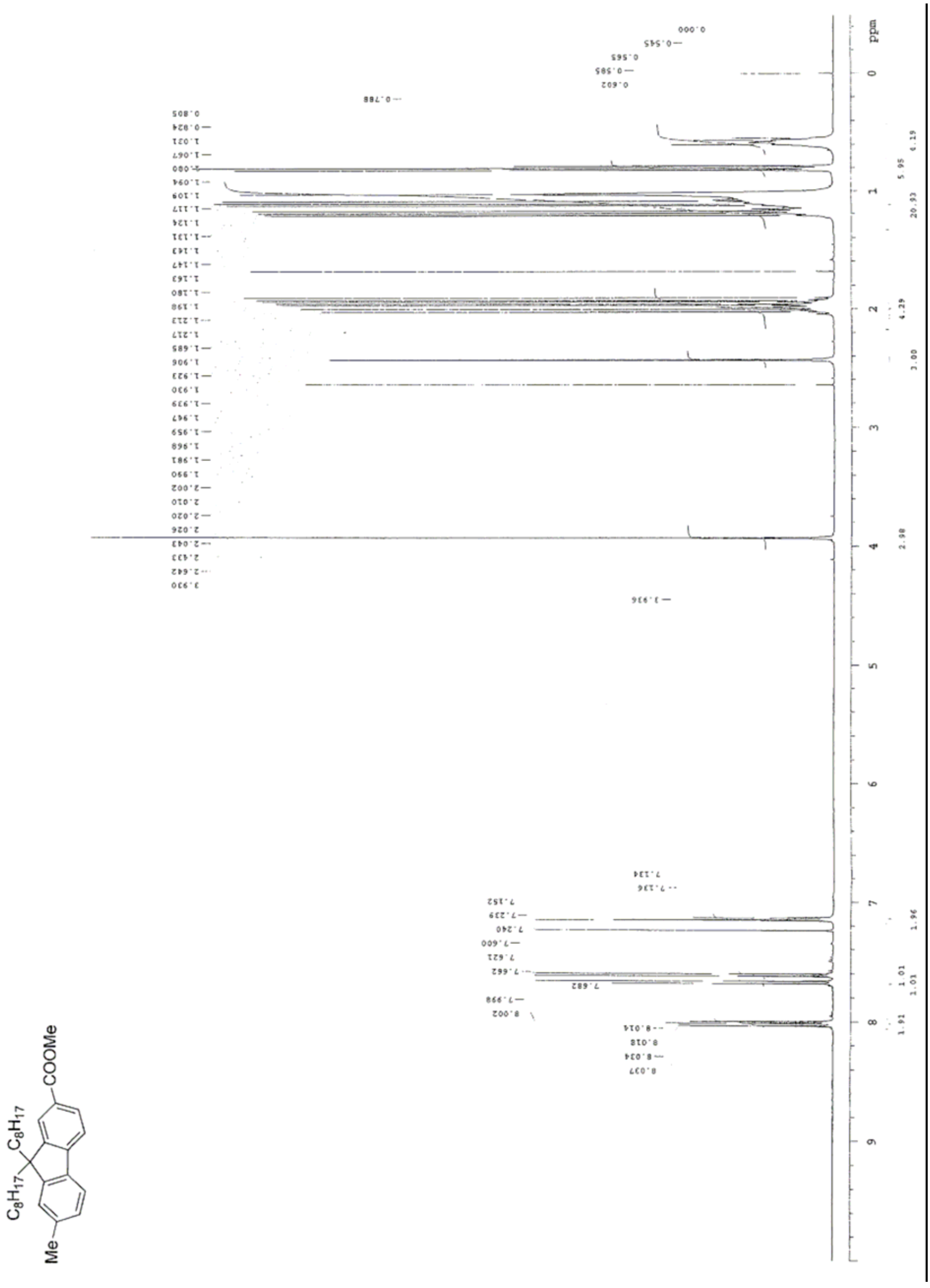

^{1}H NMR spectrum of 2-methyl-7-methoxycarbonyl-9,9-dioctylfluorene (50)

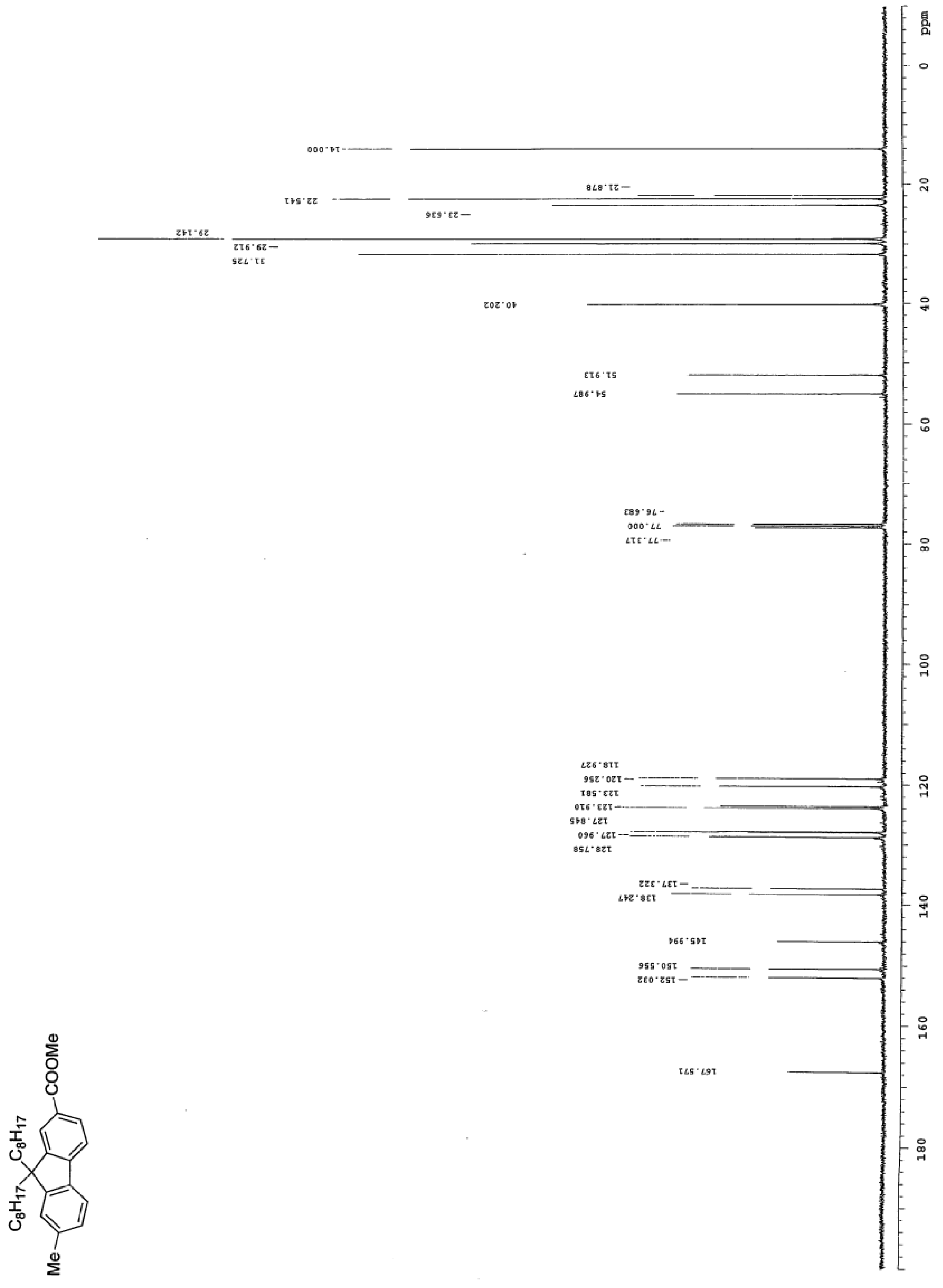

^{13}C NMR spectrum of 2-methyl-7-methoxycarbonyl-9,9-dioctylfluorene (50)

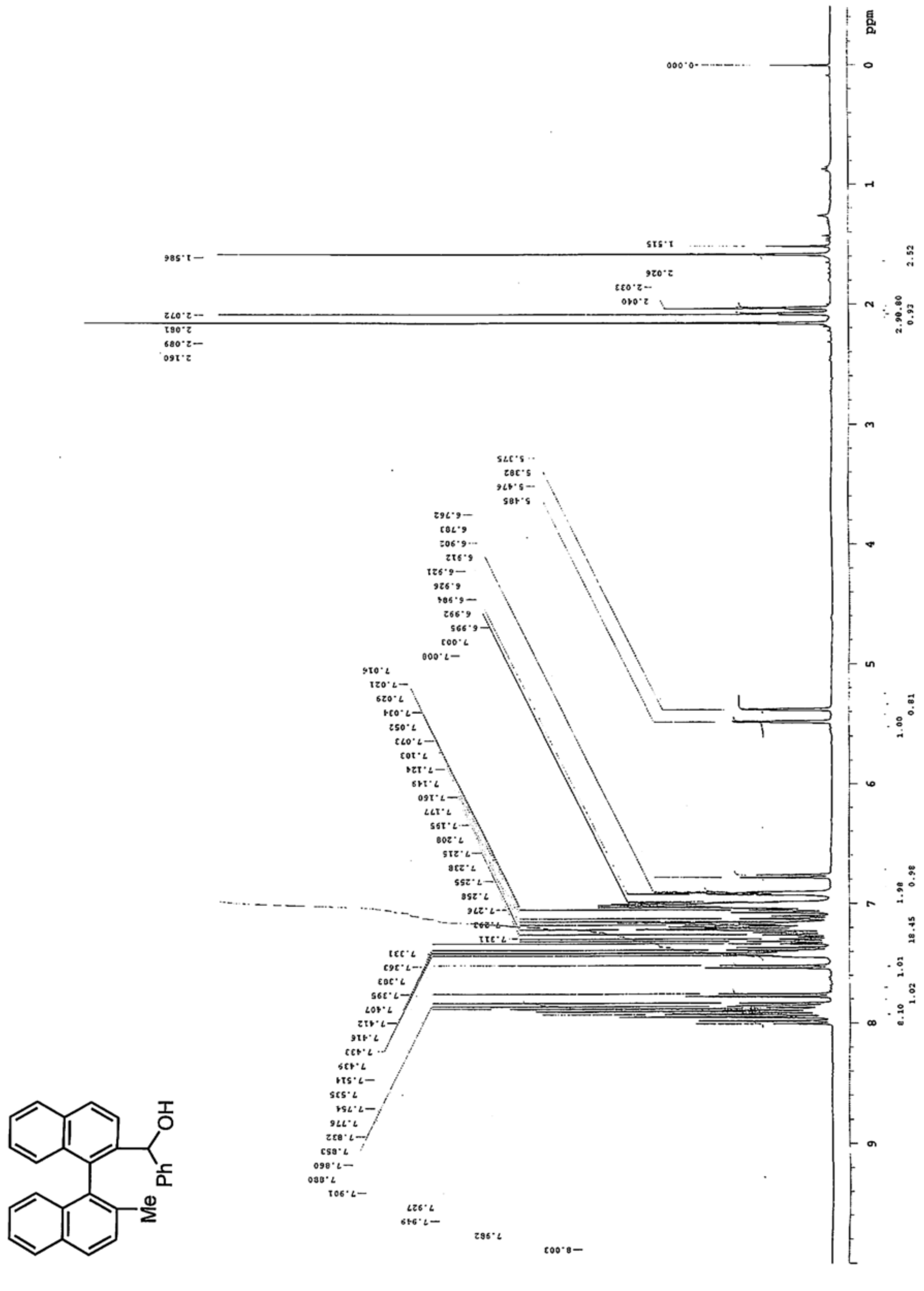

^{1}H NMR spectrum of 2-methyl-2’-[hydroxyl(phenyl)methyl]binaphthyl (51)
(diastereomer ratio = 56/44)

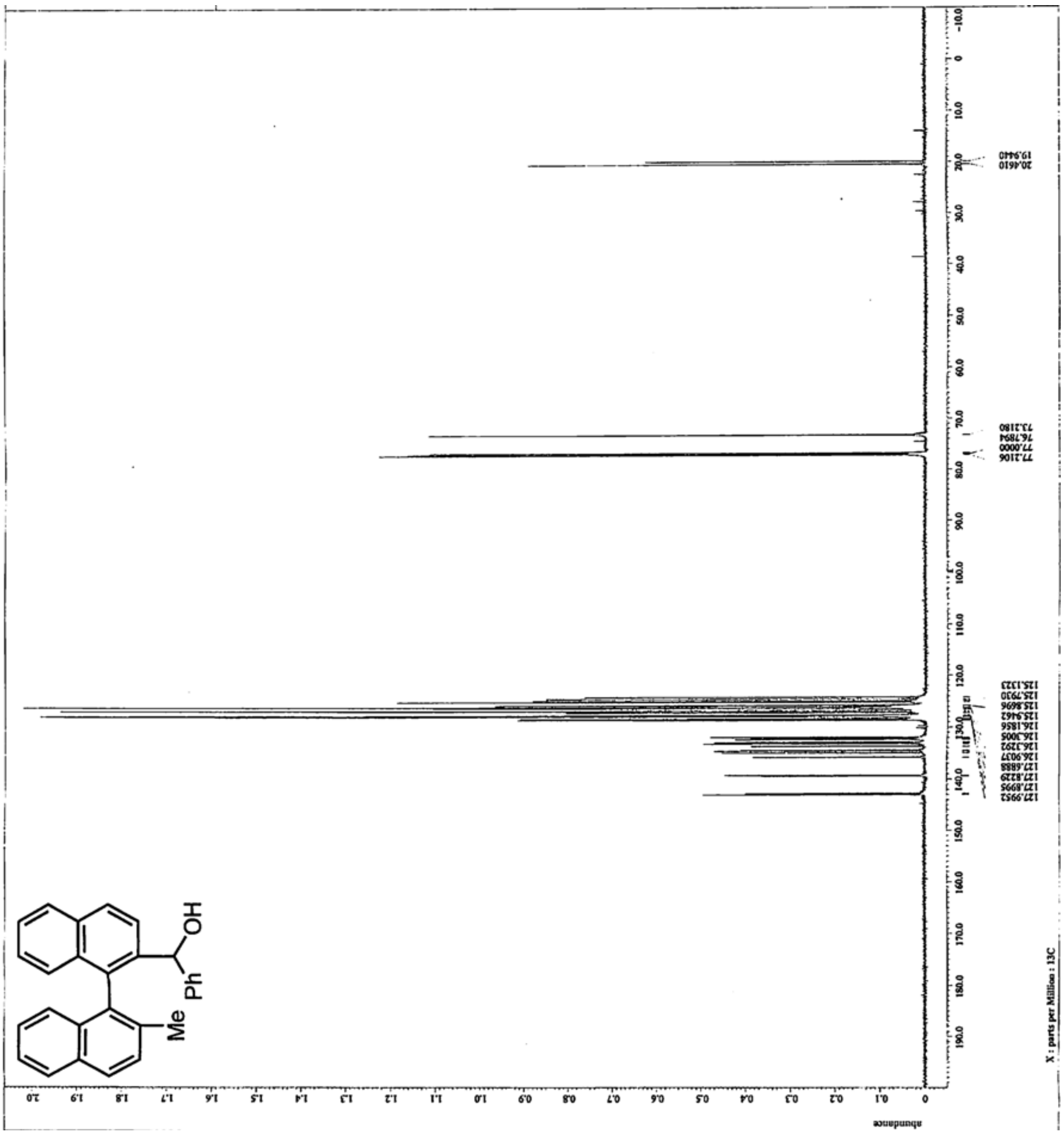

^{13}C NMR spectrum of 2-methyl-2'-[hydroxyl(phenyl)methyl]binaphthyl (51)
(diastereomer ratio = 56/44)

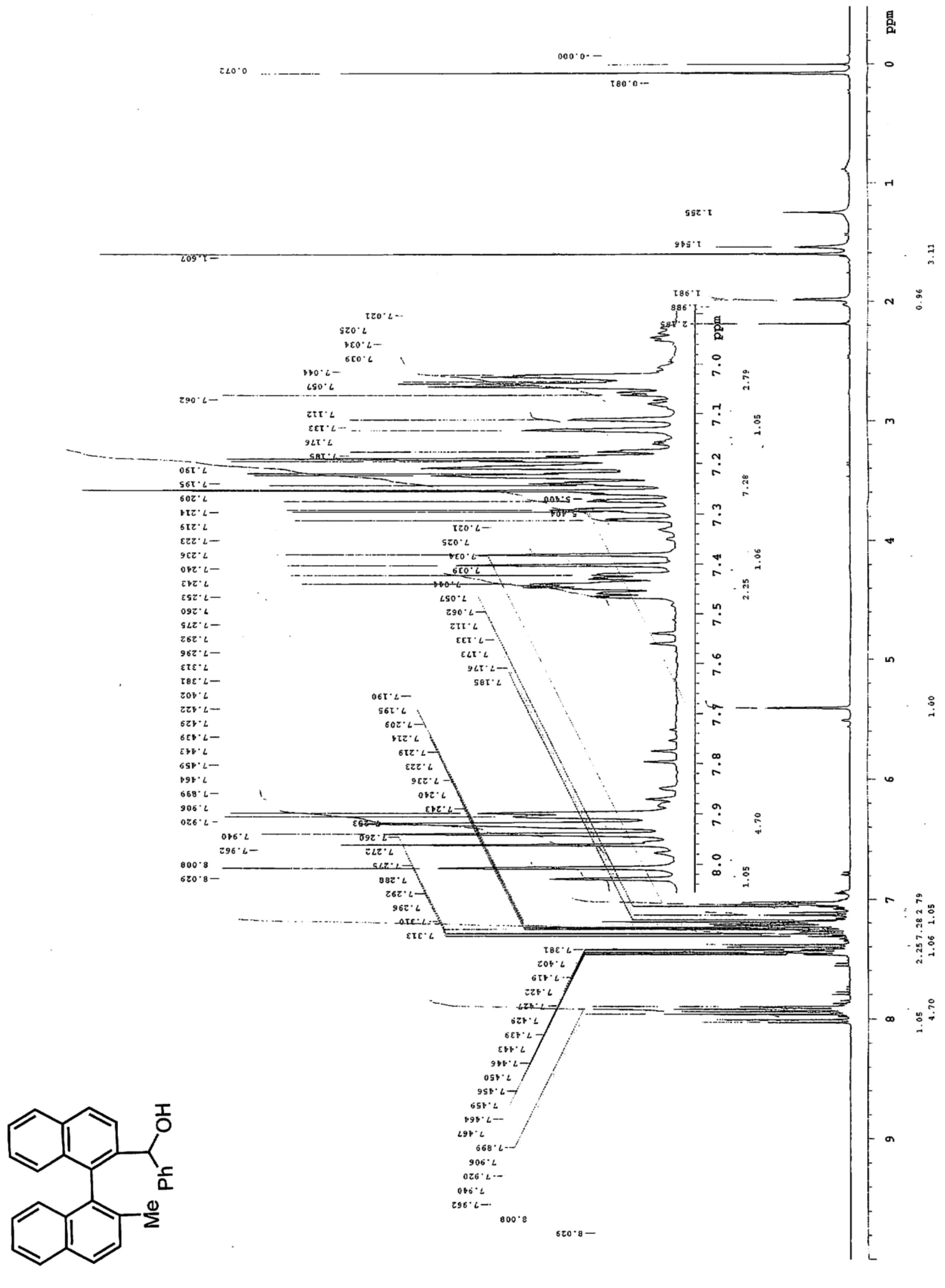

^{1}H NMR spectrum of 2-methyl-2'-[hydroxyl(phenyl)methyl]binaphthyl (**51**)
(diastereomer ratio = 88/12)

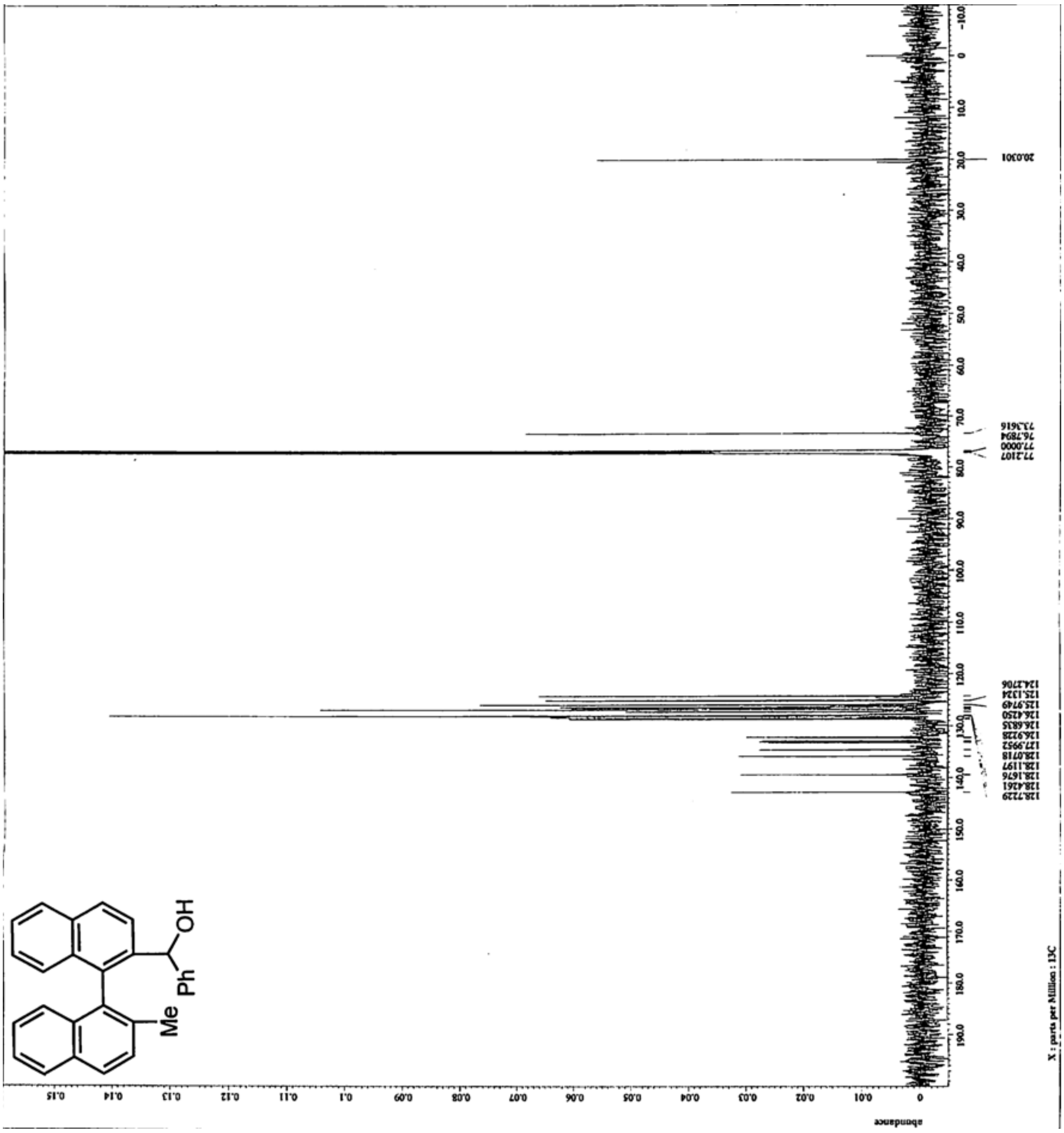

^{13}C NMR spectrum of 2-methyl-2’-[hydroxyl(phenyl)methyl]binaphthyl (51)
(diastereomer ratio = 88/12)

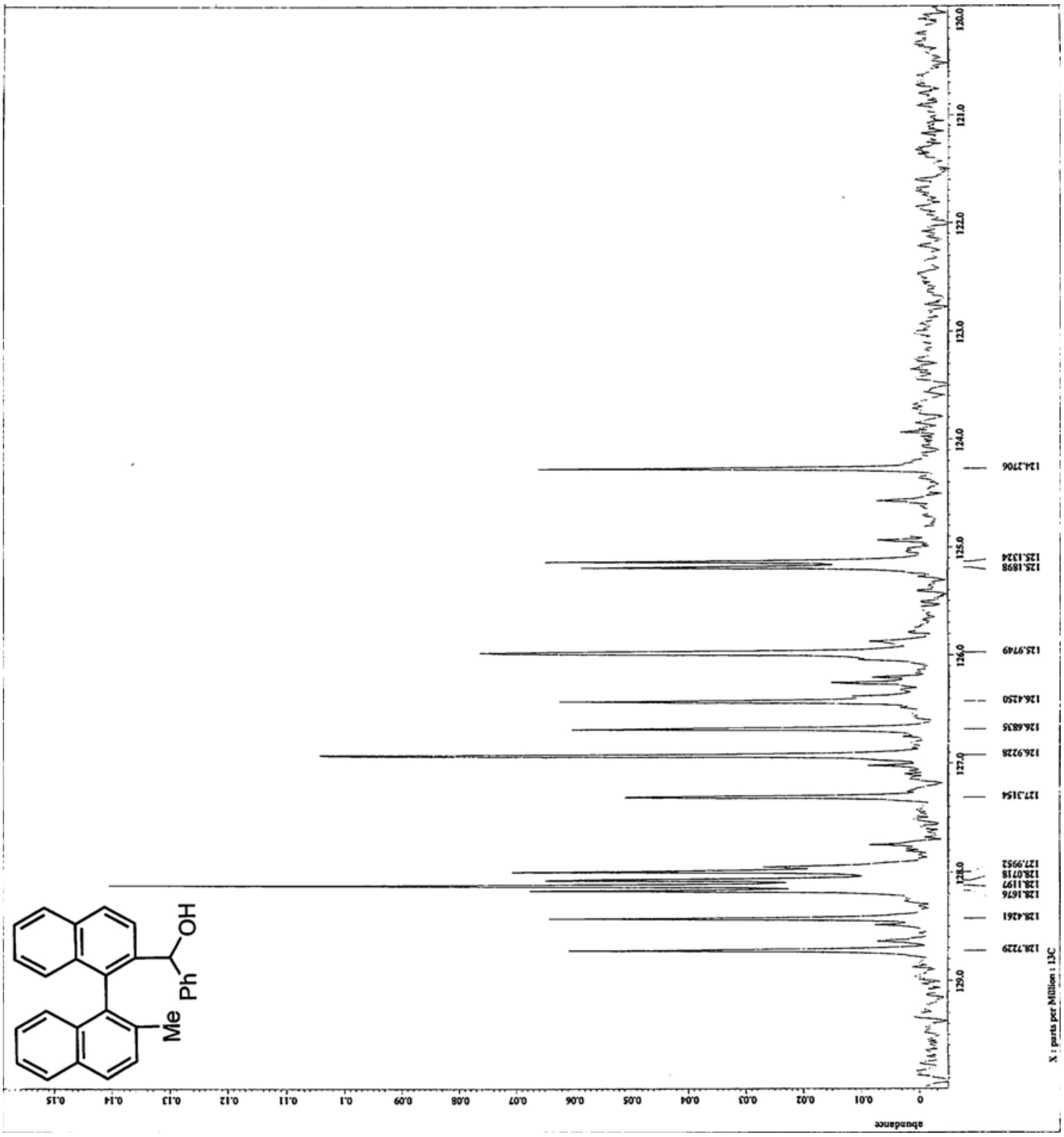

^{13}C NMR spectrum of 2-methyl-2'-[hydroxyl(phenyl)methyl]binaphthyl (51) **(diastereomer ratio = 88/12)**

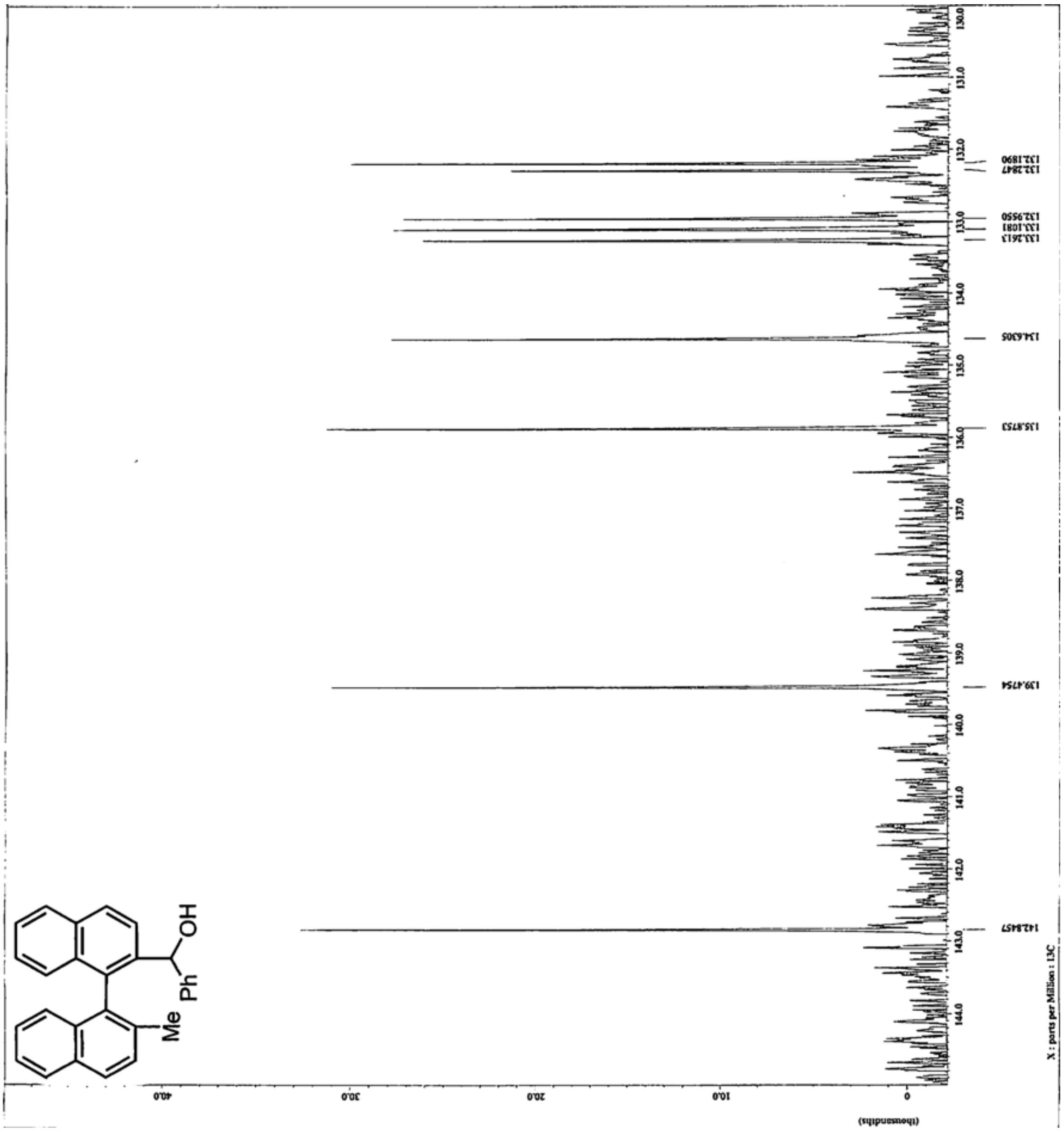

^{13}C NMR spectrum of 2-methyl-2'-[hydroxyl(phenyl)methyl]binaphthyl (51)
(diastereomer ratio = 88/12)

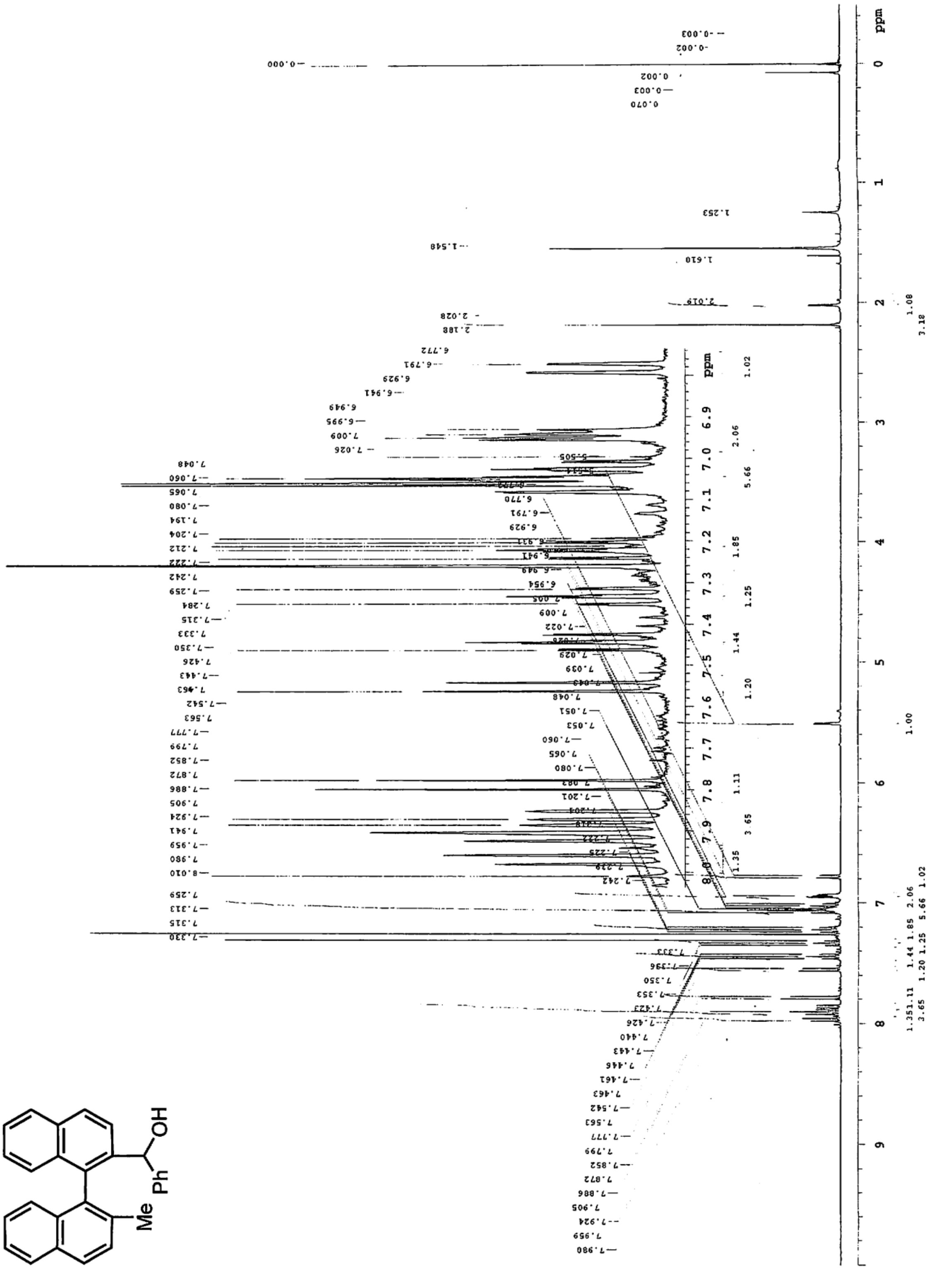

^{1}H NMR spectrum of 2-methyl-2’-[hydroxyl(phenyl)methyl]binaphthyl (51)
(diastereomer ratio = 12/88)

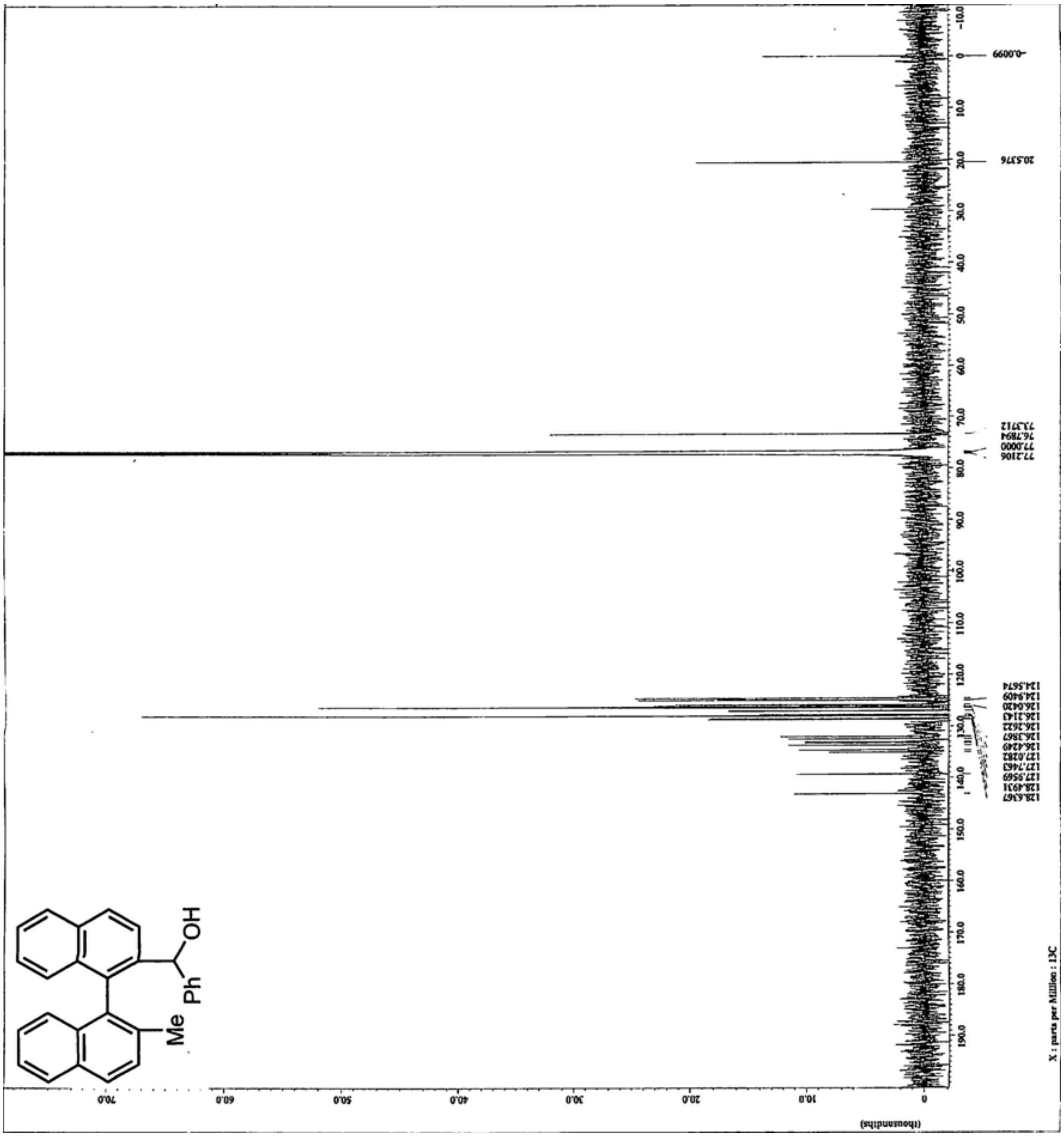

^{13}C NMR spectrum of 2-methyl-2’-[hydroxyl(phenyl)methyl]binaphthyl (51)
(diastereomer ratio = 12/88)

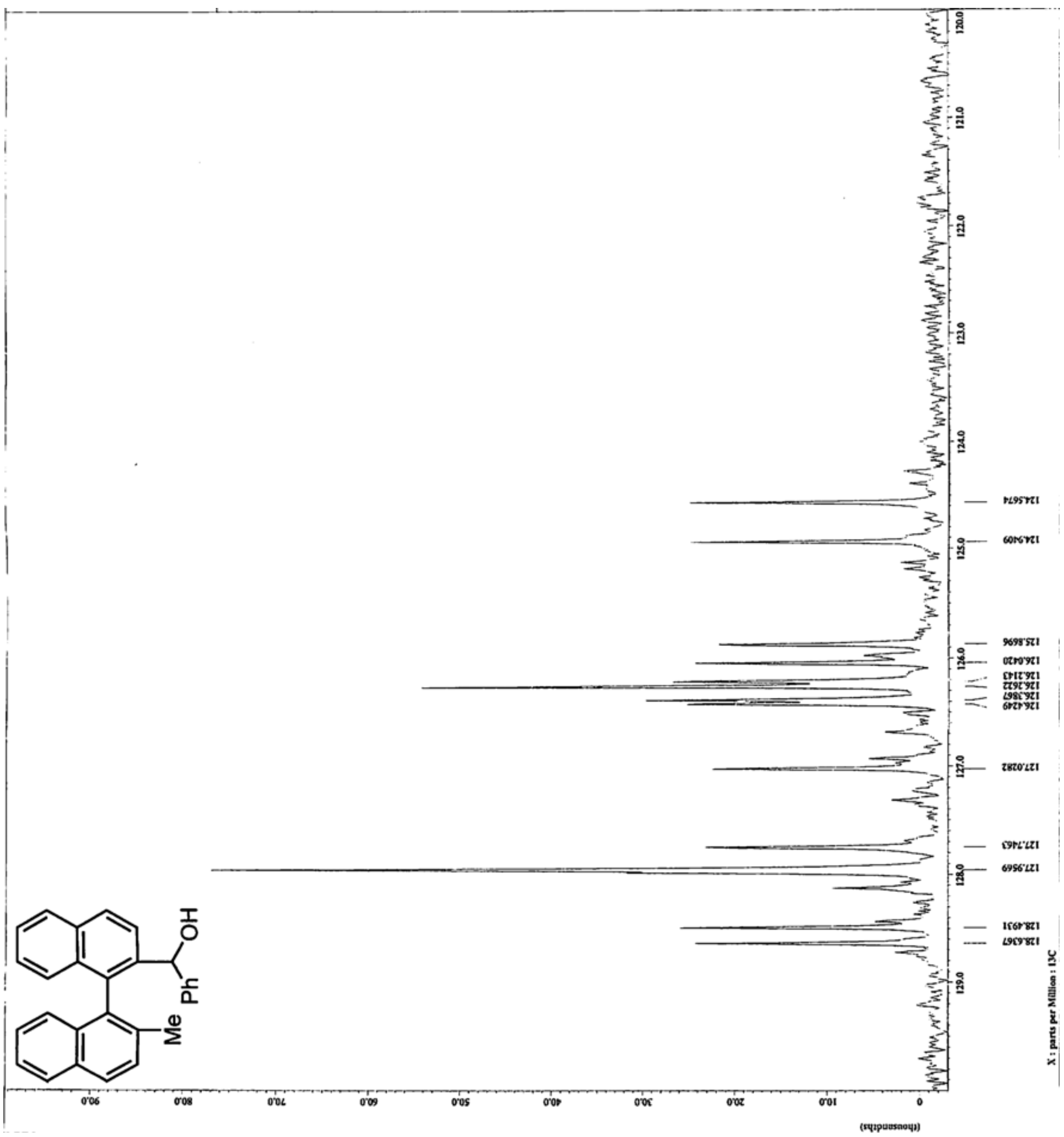

^{13}C NMR spectrum of 2-methyl-2’-[hydroxyl(phenyl)methyl]binaphthyl (**51**)
(diastereomer ratio = 12/88)

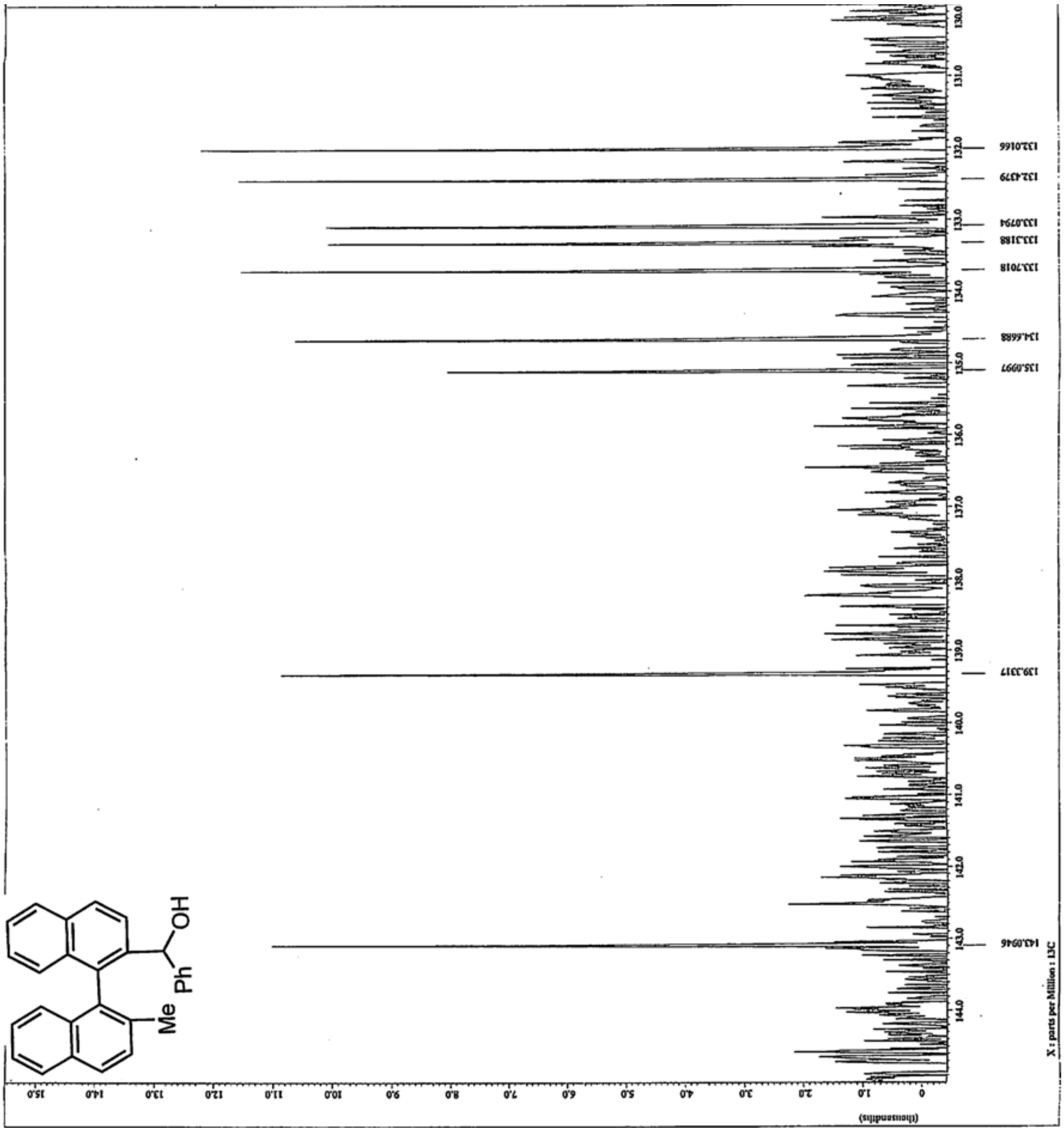

^{13}C NMR spectrum of 2-methyl-2'-[hydroxyl(phenyl)methyl]binaphthyl (**51**)
(diastereomer ratio = 12/88)

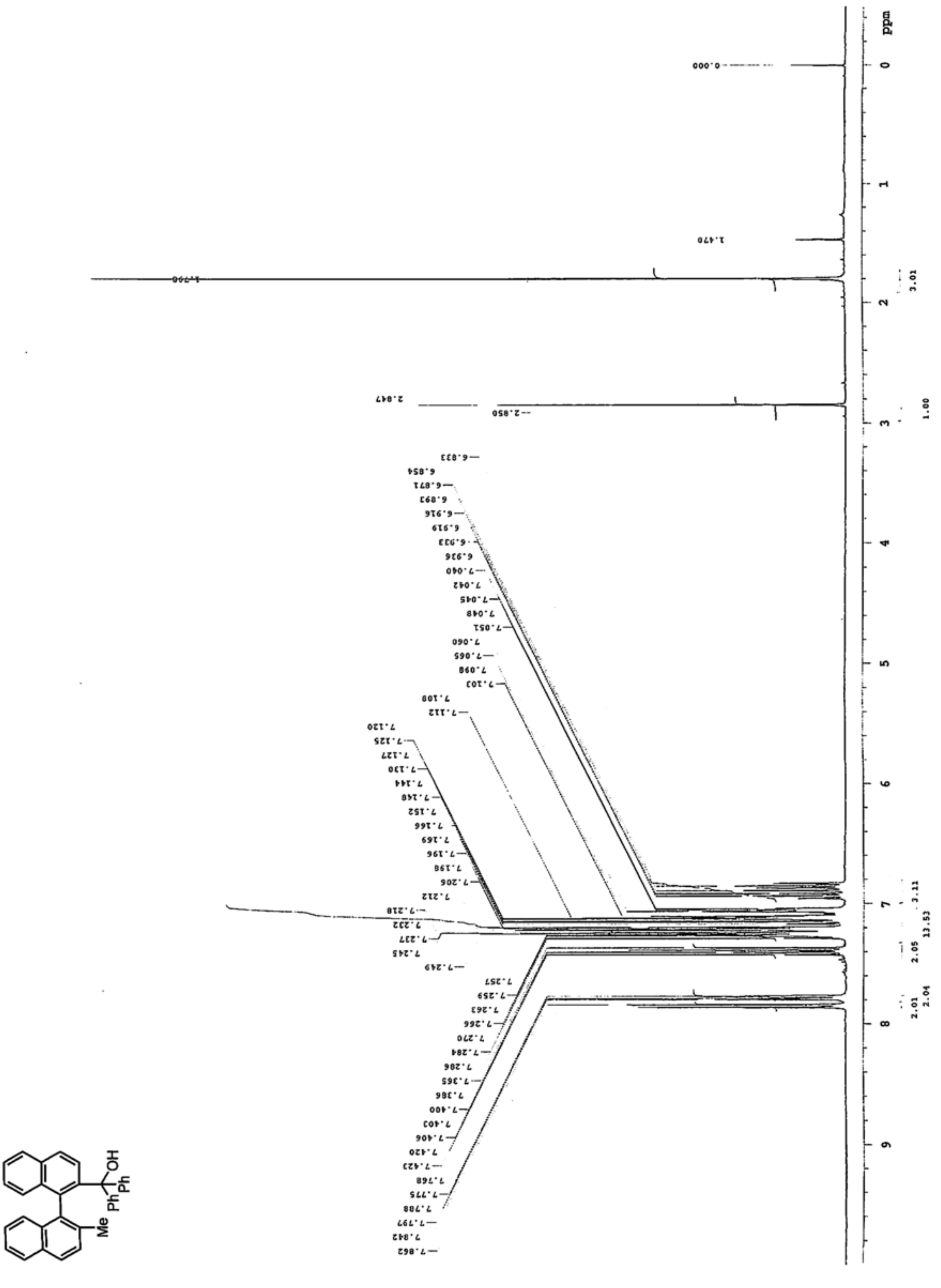

^{1}H NMR spectrum of 2-methyl-2’-[hydroxyl(diphenyl)methyl]binaphthyl (**52**)

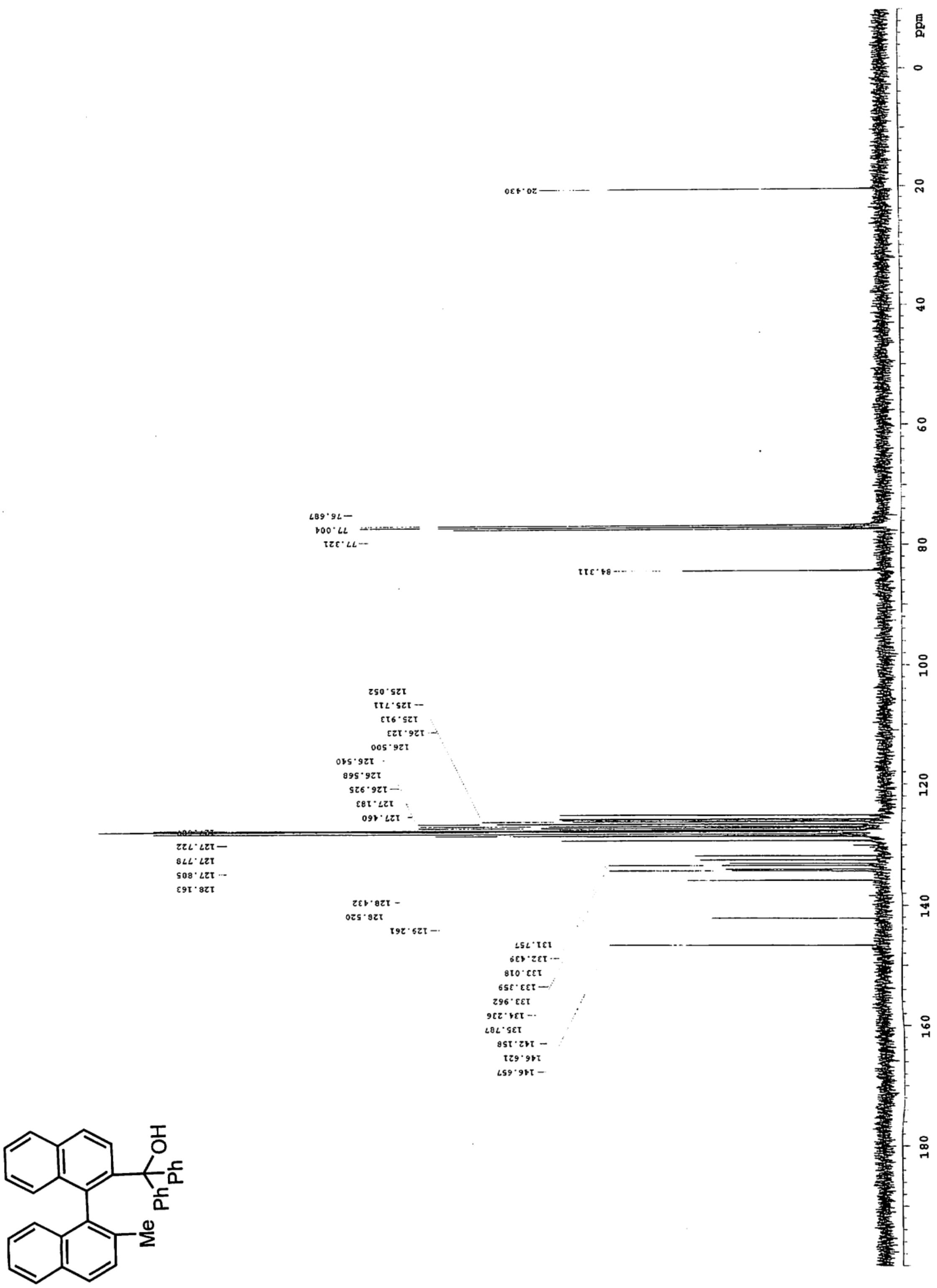

^{13}C NMR spectrum of 2-methyl-2’-[hydroxyl(dipheny)lmethyl]binaphthyl (52)
